# Design of a Multiuse Photoreactor To Enable Visible‐Light Photocatalytic Chemical Transformations and Labeling in Live Cells

**DOI:** 10.1002/cbic.202000392

**Published:** 2020-09-16

**Authors:** Noah B. Bissonnette, Keun Ah Ryu, Tamara Reyes‐Robles, Sharon Wilhelm, Jake H. Tomlinson, Kelly A. Crotty, Erik C. Hett, Lee R. Roberts, Daria J. Hazuda, M. Jared Willis, Rob C. Oslund, Olugbeminiyi O. Fadeyi

**Affiliations:** ^1^ Exploratory Science Center Merck & Co. Inc. 320 Bent Street Cambridge MA 02141 USA; ^2^ Infectious Diseases and Vaccine Research Merck & Co. Inc. Merck Research Laboratories West Point PA 19486 USA; ^3^ Efficieny Aggregators, 1207 FM 359 Richmond TX 77406 USA

**Keywords:** live-cell labeling, multiplex capabilities, photochemistry, photoredox, photoreactors, proteins

## Abstract

Despite the growing use of visible‐light photochemistry in both chemistry and biology, no general low‐heat photoreactor for use across these different disciplines exists. Herein, we describe the design and use of a standardized photoreactor for visible‐light‐driven activation and photocatalytic chemical transformations. Using this single benchtop photoreactor, we performed photoredox reactions across multiple visible light wavelengths, a high‐throughput photocatalytic cross‐coupling reaction, and *in vitro* labeling of proteins and live cells. Given the success of this reactor in all tested applications, we envision that this multi‐use photoreactor will be widely used in biology, chemical biology, and medicinal chemistry settings.

## Graphical abstract

**The light between fields**: Visible‐light chemistry is increasingly being applied to probe biology, but no photoreactor exists to enable crossover from visible‐light‐mediated reactions in the flask to complex biological environments. A single multi‐use photoreactor has been developed to enable visible‐light‐driven reactions in medicinal chemistry, chemical biology, and biology settings.

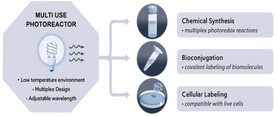

## Introduction

Photochemistry has long been used by chemists to generate reactive species to achieve unique transformations otherwise inaccessible through thermodynamic means. Molecules are electronically excited (to singlet or triplet excited states) by light absorption, promoting the formation of species with new reactivity patterns relative to the ground state.^[1]^ Small‐molecule‐based, photoactivation chemistry has a rich history in organic synthesis particularly in driving complex small‐molecule rearrangements such as isomerizations and cyclizations.[^2^, ^3^] The true power of photoactivation chemistry is best illustrated in Eschenmoser's classic total synthesis of cobyric acid, the key organic component of vitamin B_12_, where sunlight was used to catalyze a macrocyclization of the A and D rings.^[4]^

Photoactivation has also enabled the use of photoaffinity labeling within chemical biology. For these applications, substrates including aryl azides, diazirines and benzophenones are activated by UV light (∼300 nm) for covalent crosslinking to biomolecules.[^5^, ^6^] Probes based on these substrates have found applications in radiolabeling,^[5]^ drug‐antibody conjugation,^[7]^ chemoproteomics,^[8]^ target ID^[9]^ and protein crosslinking.^[10]^ In some cases, the reactive intermediate used for these applications forms from the T_1_ excited state.^[11]^ This state is formed in two steps: 1) direct excitation to S_1_ excited state using UV light, then 2) Intersystem crossing (ISC) to form the reactive T_1_ state. Many times, there is a significant energy gap between the S_1_ and T_1_ energy states, therefore there is a large energy loss due to ISC.^[12]^ If direct excitation to the T_1_ state was possible, a significantly lower energy light source could be used. However, quantum mechanics forbids direct excitation to the T_1_ state because spin is not conserved. Recognizing this inefficient use of energy, as well as the constraints due to quantum mechanics, organic chemists have made significant progress in using photosensitization and photoredox chemistry to access reactive intermediates, particularly free radicals, using lower energy excitation sources.^[13]^ Over the last decade, an unprecedented number of highly valuable synthetic transformations have been developed by using photoredox chemistry.^[14]^

Photoredox catalysis reactions[^15^, ^16^, ^17^, ^18^] have gone from obscure to commonplace in both industry and academia. This upheaval can largely be attributed to the development of a uniform tool set used to perform photoredox chemistry. This toolset includes both photocatalysts as well as a uniform photoredox reactor with efficient cooling and modular light intensity.^[19]^ Commercial reactors have standardized and enabled the fine tuning of reaction conditions and more recently have become multiplex in design. Multiplex or high‐throughput synthesis (HTS) enables speedy reaction optimization through screening multiple reaction conditions at once. Additionally, multiplex synthesis can empower medicinal chemists to generate multiple analogs in parallel, allowing efficient collection of structure‐activity relationship (SAR) data. Considering current reactor capabilities, chemists looking to use multiplex photoredox synthesis must choose between buying multiple singleton reactors, which can be expensive, or designing their own, which can introduce variabilities in light exposure and heating between devices. Although some HTS photoredox reactors have been developed, they are solely engineered for small‐molecule transformations.^[20]^ Thus, an ideal all‐purpose photoreactor is currently lacking. Furthermore, safety concerns can arise with DIY systems as strong blue LED light can be particularly harmful to the eye.^[21]^ Although tinted shields and glasses are available to block blue wavelengths, engineering a self‐contained system would aid in limiting exposure to potentially harmful wavelengths.

**Figure 1:**
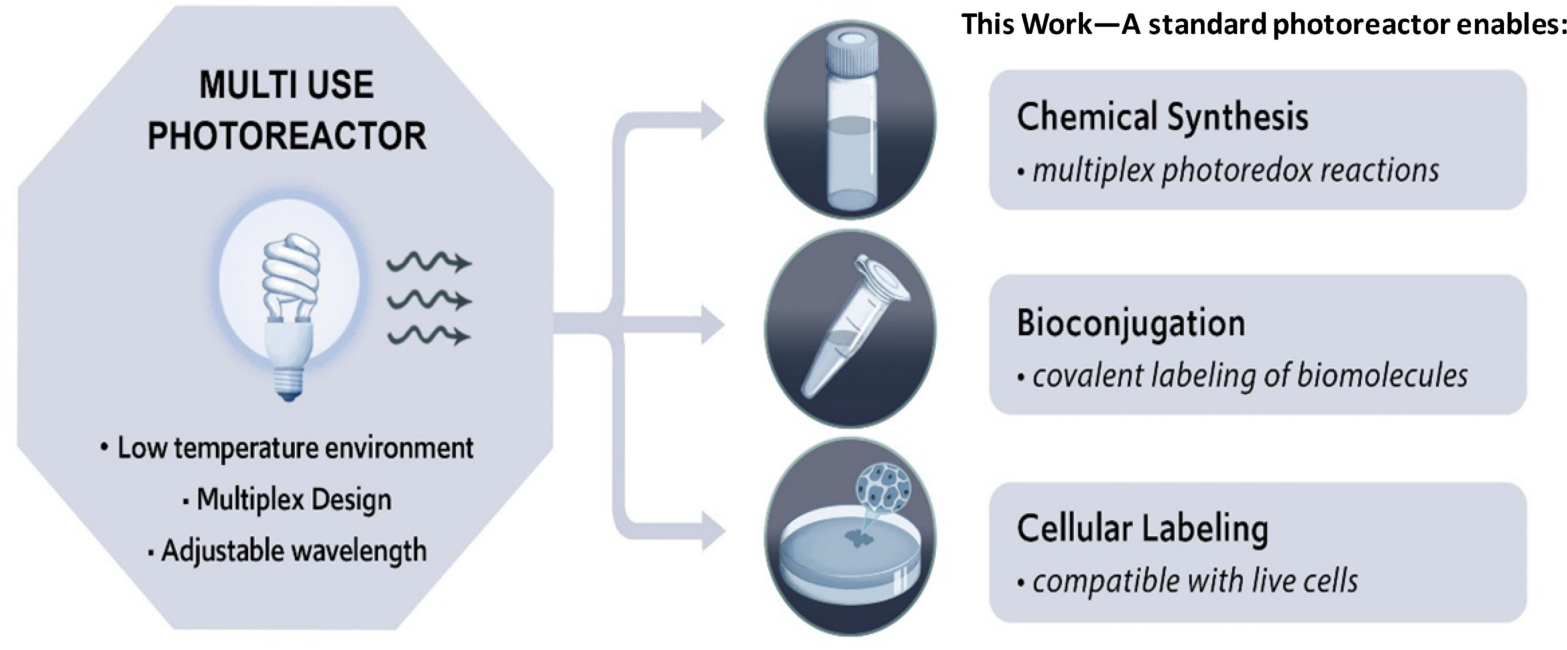
Features desired in an ideal photoreactor that would enable chemical synthesis, bioconjugation, and cellular labeling reactions.

The boundaries of visible light photochemistry have been pushed into protein and live systems by emerging methodologies for peptide and biomolecule functionalization,[^22^, ^23^, ^24^, ^25^] bioconjugation,^[26]^ protein labeling,[^27^, ^28^] and crosslinking.^[29]^ Recently, we reported the development of a photocatalytic‐based proximity labeling platform for mapping protein microenvironments on live mammalian cell surfaces.^[30]^ The successful applications of photocatalysis within biological systems will continue to increase the utility of this approach to explore biology. However, photocatalytic conditions can limit live‐cell applications leading to a need for suitable biocompatible tools and reagents. Surprisingly, a uniform visible‐light photoreactor for probing biological systems is nonexistent. Relative to small molecules, proteins and living cells are significantly more sensitive to heat which can cause dramatic perturbations to a protein structure or physiological environment within the cell. Therefore, an ideal device would have efficient heat dissipation that can maintain samples at a low temperature. Furthermore, the optimal device would be compatible with multiple samples in order to ensure uniformity across a data set or multiple reactions. A final important feature of this new device would be the ability to function at different wavelengths. Although blue light is used in most photoredox methodologies, new methodologies that use lower energy green^[31]^ and red light[^32^, ^33^] have emerged. In addition to the chemical transformations achieved by these lower energy light sources, longer wavelengths have significantly better penetration into living tissue.^[34]^ As such, a uniform device integrating all of these features would be widely employed within the medicinal chemistry, chemical biology, and biology communities. Herein we, describe the design and development of a multi‐use, low heat photoreactor to enable multiplex photoredox reactions, covalent labeling of biomolecules, and labeling within live cells (Figure 1). We showcase a proof of concept framework for known light‐driven chemical transformations at different wavelengths, a model photoredox HTS Buchwald coupling on drug‐like scaffolds, protein biotinylation, and live‐cell labeling for confocal microscopy imaging analysis.

## Results and Discussion

### Photoreactor design and development

**Figure 2:**
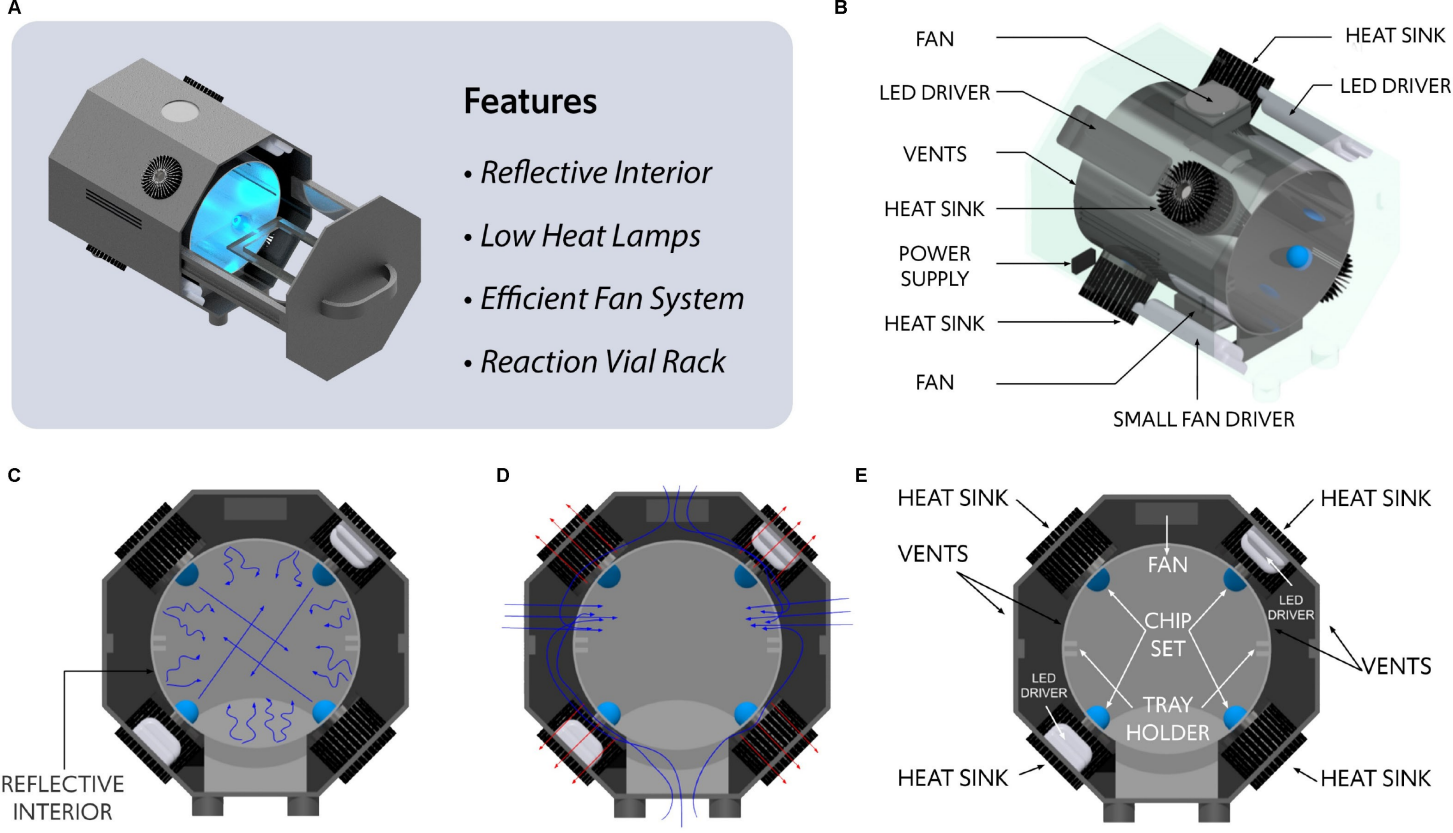
Bio‐photoreactor schematic diagrams. A) Bio‐photoreactor with highlighted features. B) Cutaway view of inner chamber with external components marked. C) Internal view highlighting the reflective interior of the inner chamber for efficient light distribution. D) Internal view of inner and outer chambers showing heat flow. Red and blue arrows indicate hot and cool air flow, respectively. E) Internal cutaway view showing the inner and outer chambers with components marked.

To develop a photoreactor that enables multifunctional application in biology and chemistry settings, the reactor size needs to accommodate multiple reaction vials of different types and sizes (e. g., dram vials, microcentrifuge tubes, cell culture plates) with even light distribution across the entire reactor space. To achieve this goal, we designed an oven‐styled photoreactor consisting of two chambers. The outer chamber contains a pull‐out lid for sample insertion onto interchangeable, translucent reaction trays into a cylindrical inner chamber containing low heat light chips (Figure 2a). To maximize light coverage, the inner chamber cylinder (Figure 2b) was designed using 6063 T5 aluminum polished to a mirror finish to minimize light absorbance and optimize the light scattering effect (Figure 2c). Four LED‐based chips (455 nm, 555 nm, or 660 nm wavelengths) were arrayed in a staggered X position onto the inner chamber to create a consistent light distribution across the horizontal midline of the inner chamber and eliminate any shadowing effect from reaction samples (Figure 2b). To dissipate heat generated from the LEDs, heat sinks were physically attached to each light chip that extend into the outer chamber of the reactor (Figure 2d). This heat sink technology functions through absorbing heat away from the LED chips into the heat sink core and fins located in the outer chamber. Although the device relies primarily on passive cooling within the heat sinks, two fans (80 mm, 2200 rpm at 100 % output) were mounted to the inner wall of the outer chamber on the top and bottom center of the device (Figure 2e). The fans pull air from outside of the device to displace radiant heat within the inner and outer chambers and cool the heat sink fins. In order to facilitate easy removal and replacement of the four LED chips, each LED/heat sink assembly connects to the inner chamber via wireless magnetic contact to electrical contact pins. Finally, the device was designed to be controlled wirelessly through a mobile app via a Bluetooth (BLE) antenna connected inside of the outer chamber to control photoreactor light intensity (0–100 %), light duration (h/min/s), and cooling fan percent output (0–100 %).

### Multiplex of photocatalytic chemical transformations

**Figure 3:**
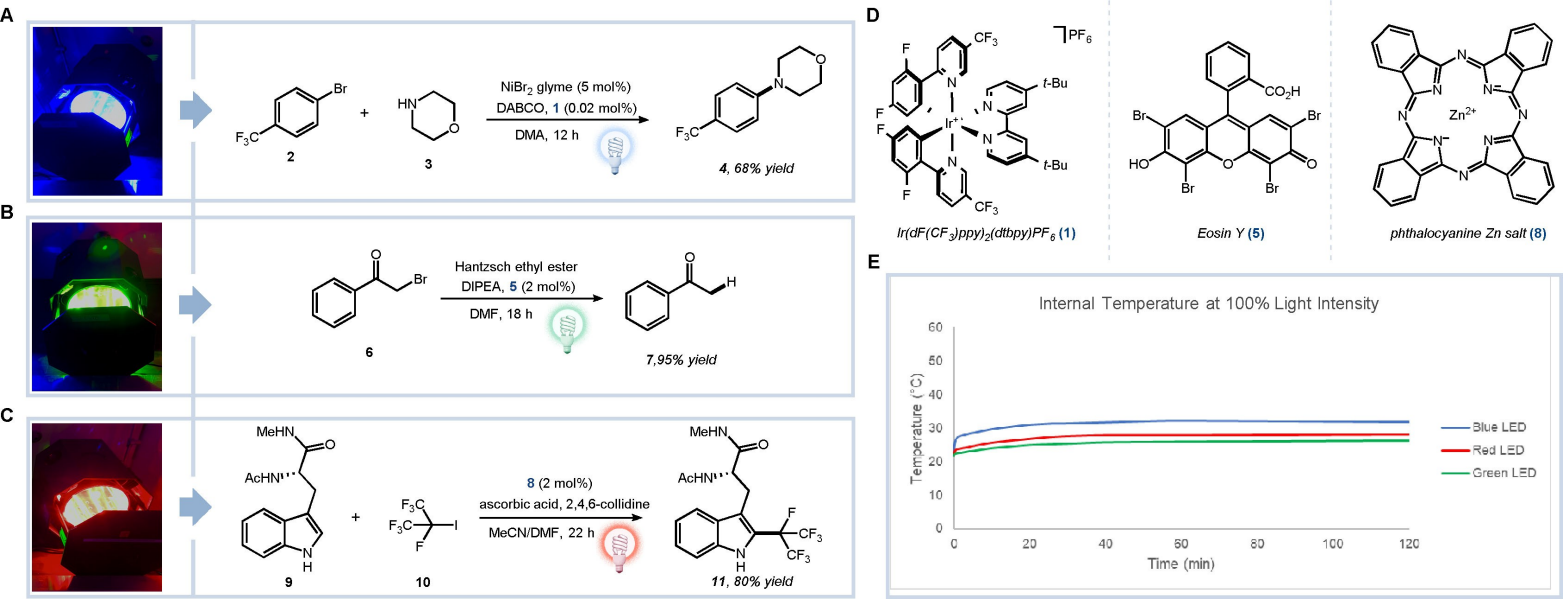
Magnetically interchangeable LED lights allow light irradiation at A) blue (455 nm), B) green (555 nm), and C) red (660 nm) wavelengths. D) Photocatalysts used across the different wavelengths for the indicated chemical transformations in A–C, E) Recorded temperature over time for blue, green and red LED lights run at 100 % intensity.

Given the historical impact of photoredox catalysis in advancing the field of synthetic organic chemistry, we explored the utility of the photoreactor by first investigating three photocatalytic synthetic transformations at different visible light wavelengths. We began our photoreactor evaluation studies utilizing the protocol for the elegant photoredox‐mediated iridium (**1**)**/**nickel dual catalyzed C−N coupling of trifluoromethyl bromobenzene (**2**) and morpholine (**3**) reported by Macmillan et al.^[35]^ Using a two‐dram vial that easily fits into a single spot of the 24‐hole array tray of this new reactor, the reaction was performed under blue light (455 nm) irradiation using nickel(II) bromide glyme and {Ir[dF(CF_3_)ppy]_2_(dtbbpy)}**⋅**PF_6_ (**1**) to successfully give the desired coupled product (**4**) in 68 % yield (Figures 3a and 3d).^[35]^ When this same reaction was performed simultaneously across all 24 vial spots of the reaction tray, similar reaction conversions were achieved (Figure S1 in the Supporting Information). While this experiment does not indicate the light intensity variability at different spots of the reactor, the similar conversions at each reaction spot clearly shows the suitability of this device for photoredox‐mediated library synthesis. To highlight the readily interchangeable LED chips feature of the reactor, we next performed a photocatalytic dehalogenation reaction reported by Zeitler et al. In the presence of organic photocatalyst Eosin Y (**5**), under irradiation with lower energy green light in the photoreactor (555 nm), dehalogenation of an α‐bromoacetophenone (**6**) proceeded quantitatively (Figure 3b and d).^[31]^ Inspired by the red light‐based photocatalytic fluoroalkylation of heteroarenes reported by Postigo et al.,^[32]^ we set out to functionalize capped‐tryptophan (**9**) using a interchangeable red light (660 nm) LED chip. In the presence of 1,1,1,2,3,3,3‐heptafluoro‐2‐iodopropane (**10**), tryptophan easily underwent C−H functionalization to give excellent yield (80 %) of the fluoroalkylated‐tryptophan product using a red‐light‐activated zinc phthalocyanine photocatalyst (**8**; Figure 3c and d). The efficient coupling yield of the tryptophan amino acid under red light illumination showcases the potential opportunity to utilize this transformation in an *in vivo* or tissue setting for tryptophan labeling. Finally, for all three wavelengths the internal heat temperatures were measured and observed to not exceed ∼30 °C when run at 100 % light intensity over time indicating the ability to maintain low temperature within the device (Figure 3e). The three model reactions tested within the integrated photoreactor at multiple LED wavelengths and the low temperatures maintained will permit the development and application of novel chemical transformations for profiling biological environments, particularly where higher temperatures can have adverse effects on protein stability or cell viability.

**Figure 4:**
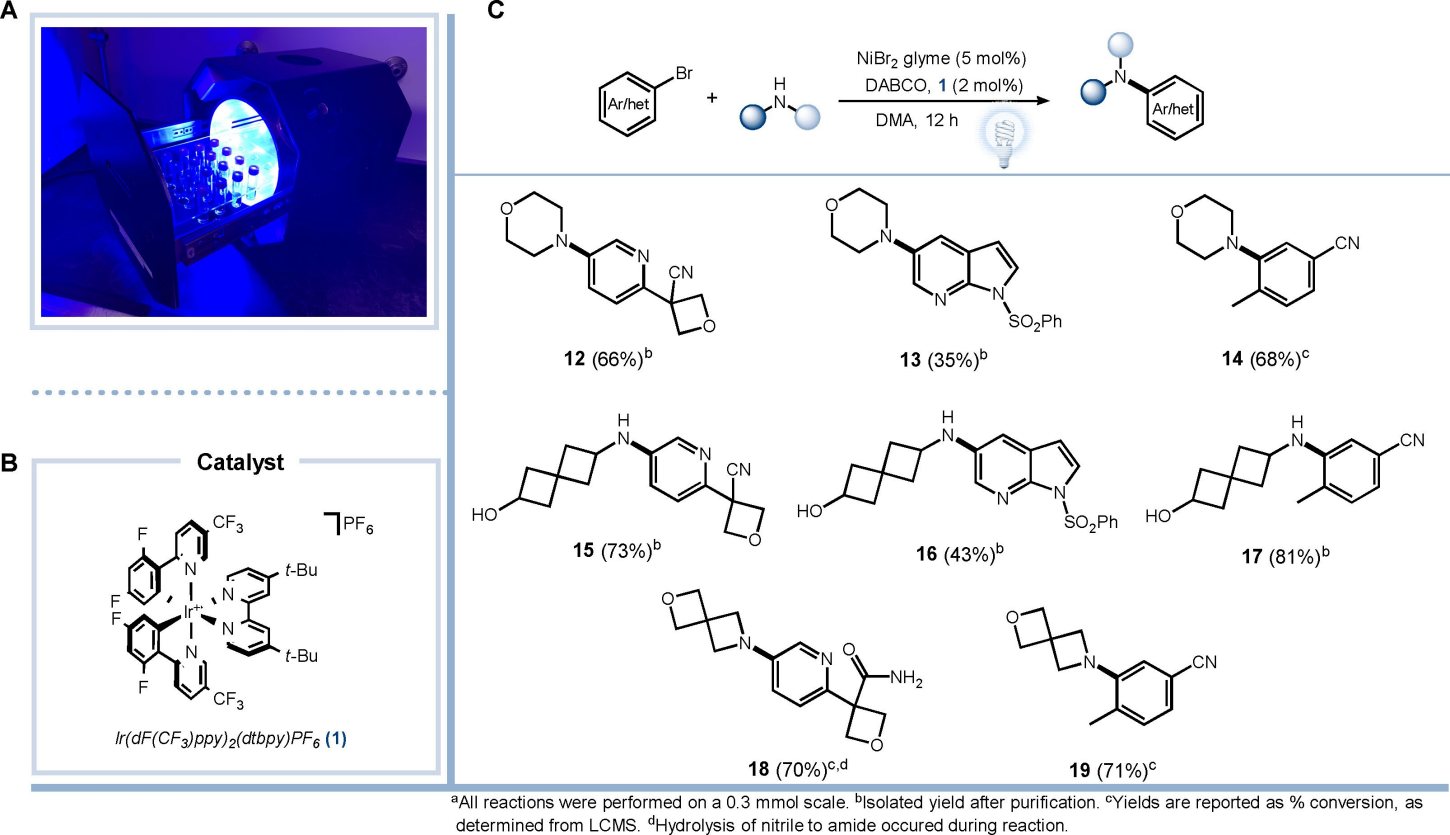
Photocatalytic reaction multiplexing. A) Photoreactor with reaction tray loaded with dram vials for parallel synthesis. B) Iridium catalysts used for library synthesis. C) Parallel‐scope photoredox Buchwald C−N coupling reaction.

HTS capabilities have served a vital role in medicinal chemistry efforts to perform SAR studies for drug design. Therefore, the ease to quickly access various analogs of a bioactive compound is critical for the cycle time of synthesis to biological testing. To explore the ability of this new integrated photoreactor to enable parallel library synthesis (Figure 4a), we selected photocatalytic Buchwald C−N coupling reactions, one of the most frequently utilized reactions in drug discovery. Using an Iridium photocatalyst (**1**; Figure 4b), the photoreactor enabled the concurrent synthesis of functionalized bioactive molecules (Figure 4c). All the coupled monomers feature elements commonly found in medicinal chemistry such as high fraction sp^3^ containing monomers, basic amines, and highly functionalized heterocyclic cores. The ability to rapidly screen multiple photoredox‐based transformations involving different functional groups would be of large interest to medicinal chemistry groups to quickly access useful compound targets for SAR studies using a benchtop reactor. These results further highlight the utility of the photoreactor's multiplex design for overcoming issues associated with concurrent running of multiple photoredox reactions that currently rely on the use of multiple singleton reactors, or the building of custom reactors by using Kessil lamps or LED strips.

### Visible‐light‐induced protein labeling

**Figure 5:**
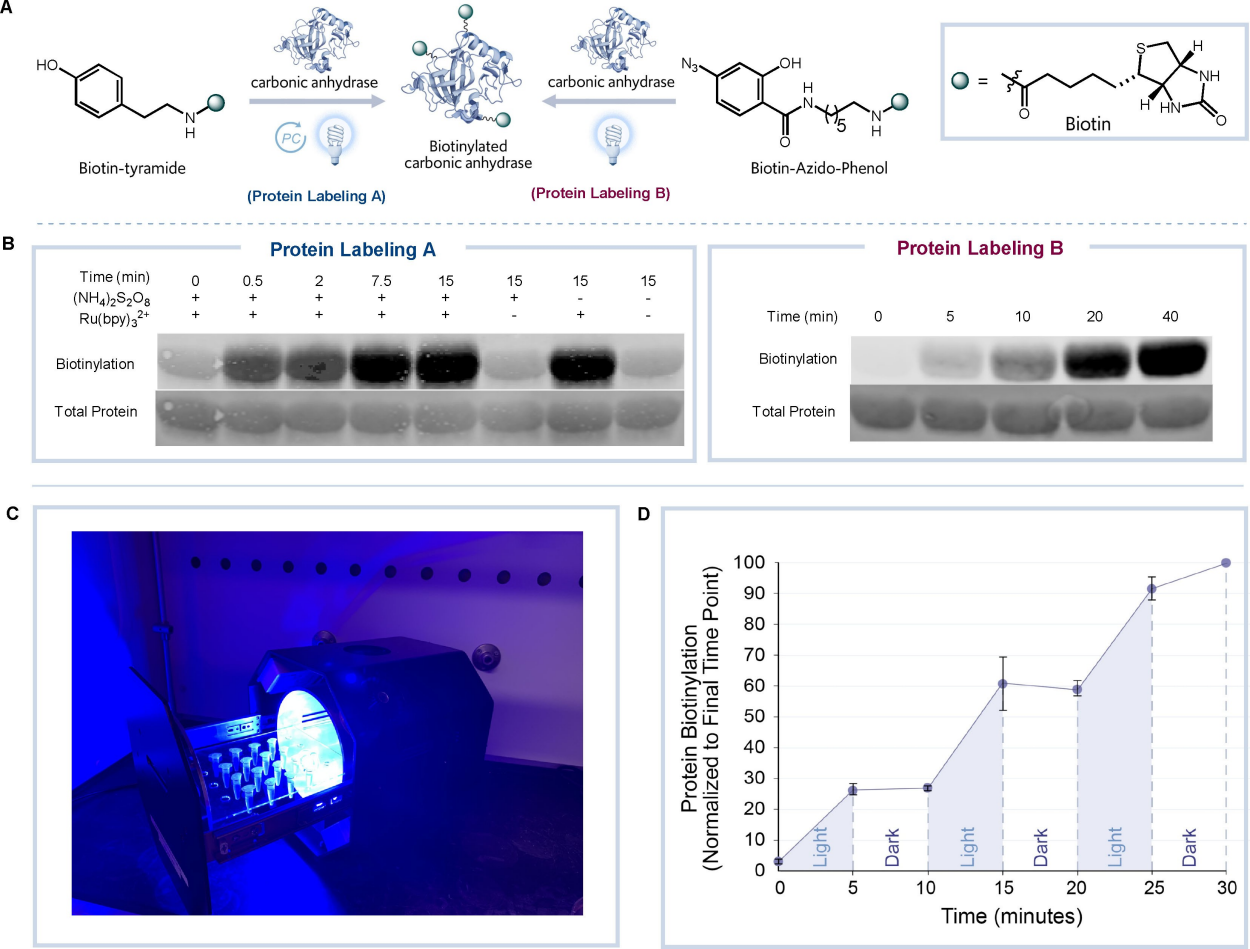
Visible‐light‐mediated protein labeling. A) General reaction scheme for the photocatalytic and photoactivatable biotinylation of CA. B) Western blot analysis shows time‐dependent biotinylation of CA. C) Bio‐photoreactor loaded with microcentrifuge tubes for reaction multiplexing. D) Biotin‐azido‐phenol on/off light experiment showcasing the light dependency of the protein labeling reaction (average of 3 independent experiments, error bars represent ± S.D.).

Successful application of the photoreactor for small‐molecule reactions at or near room temperature conditions (Figure 3e) led us to next explore protein labeling *in vitro*. A photocatalytic and photoactivatable visible‐light induced protein labeling reaction were each selected to install biotin onto carbonic anhydrase (CA) over time (Figure 5a). For the photocatalytic protein labeling system we turned to Ru(bpy)_3_^2+^ that is known to oxidize tyrosine residues to form tyrosyl radicals in the presence of blue light and ammonium persulfate ((NH_4_)_2_S_2_O_8_).^[36]^ This system has been utilized to develop protein crosslinking,^[29]^ and ligand‐directed protein labeling techniques.[^27^, ^28^, ^37^, ^38^] Inspired by these applications, we utilized this ruthenium‐based system to generate phenoxy radicals from biotin tyramide for protein biotinylation (Figure 5a, protein labeling A). We observed CA protein biotinylation upon blue light irradiation in the photoreactor that was dependent on the light exposure time (Figure 5b). The multiplex feature of the photoreactor (Figure 5c) enables the ability to irradiate multiple labeling conditions simultaneously. Accordingly, altering the reaction conditions to remove the photocatalyst and/or visible light did not lead to protein labeling (Figure 5b).

Azido phenol probes have recently been used for protein proximity labeling through a technique called enzyme‐mediated activation of radical sources (EMARs) where a peroxidase is used to generate a radical labeling species.^[39]^ As phenyl azides are known to undergo UV‐activated protein labeling,[^5^, ^7^] we wondered whether the presence of a hydroxy group on the ring system would make the probe sensitive to visible‐light activation for protein labeling as the hydroxy group has been shown to induce a bathochromic shift in absorption spectra when present on a benzene ring (Figure 5a, protein labeling B).^[40]^ CA protein was mixed with the azido phenol biotin probe and irradiated with visible light to yield time‐dependent protein labeling (Figure 5b). Additionally, a light on/off protein labeling experiment shows that biotinylation using the azido phenol biotin probe only occurs upon addition of visible light, highlighting the utility of the photoreactor to maintain exquisite control over the protein labeling reaction (Figure 5d). As a final demonstration of the light box to facilitate protein labeling, visible‐light activation of Eosin Y was used to induce protein oxidation that could be trapped by biotin hydroxylamine (Figure S2). Collectively, these results highlight the ability to achieve temporal control over protein labeling using the photoreactor and open the possibility to explore other light controlled protein labeling reactions.

### Live‐cell photochemical labeling

**Figure 6:**
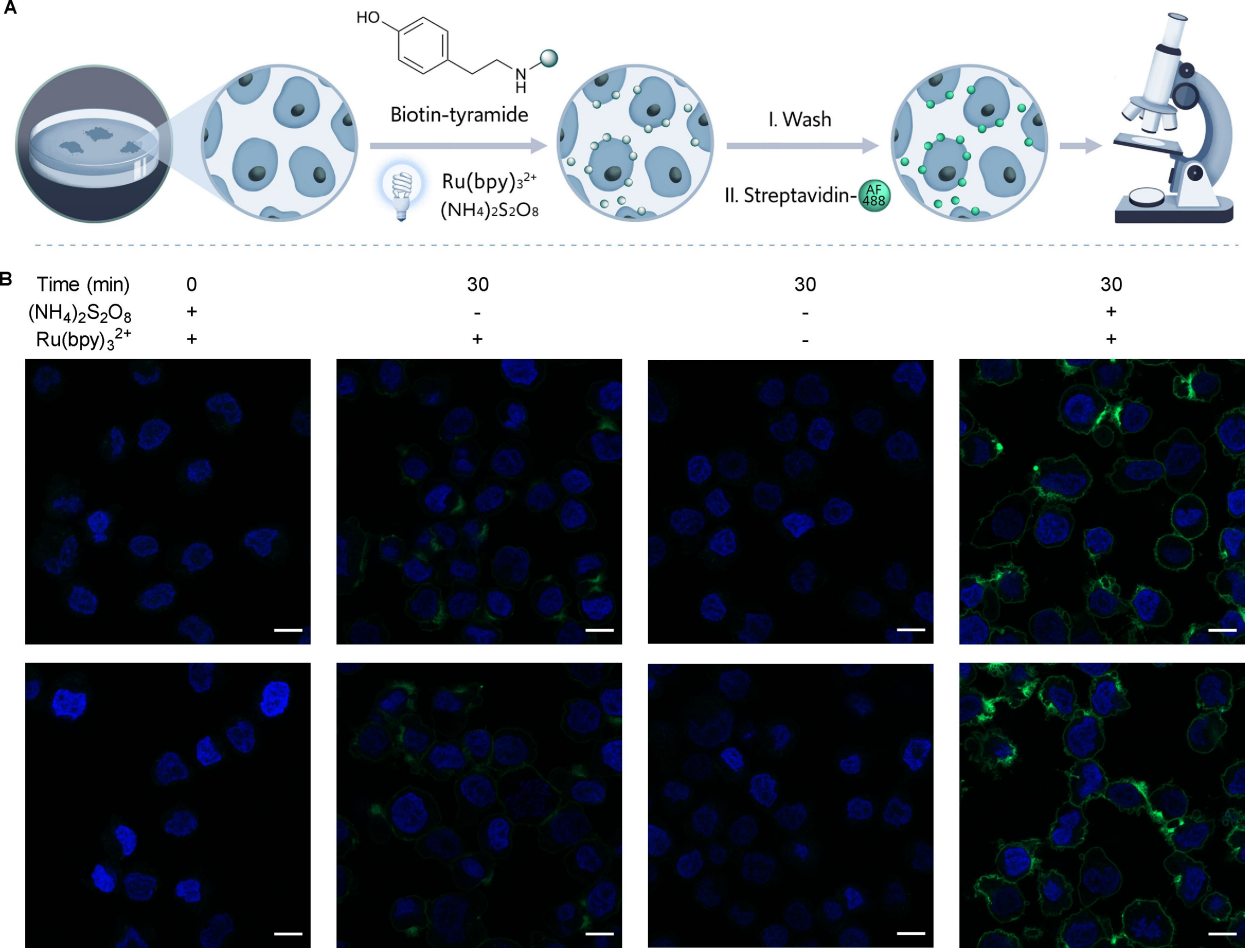
Live‐cell labeling in the multi‐use photoreactor. A) General reaction scheme for the biotinylation of A375 cells using Ru(bpy)_3_^2+^, (NH_4_)_2_S_2_O_8_, and biotin‐tyramide. B) Confocal microscopy images showing nuclei (blue) and biotinylation (green). Scale bars: 10 μm. Duplicate images are shown below each condition.

To test the feasibility of using the photoreactor for labeling of live cells, we initially determined viability of the A375 melanoma cell line upon extended exposure to full intensity visible light and observed no reduction in viability (Figure S3). Encouraged by these results, we next investigated the ability to induce protein labeling by photoredox on live A375 cells. Given the successful protein labeling results observed with the Ru(bpy)_3_^2+^ photocatalyst in Figure 5b, we selected this system for live‐cell labeling (Figure 6a). Cellular biotinylation was monitored by confocal imaging using streptavidin Alexa Fluor 488 (AF 488) conjugate dye that binds biotinylated cells after washing unreacted biotin tyramide (Figure 6a). Gratifyingly, cellular biotinylation only occurred in the presence of Ru(bpy)_3_^2+^, (NH_4_)_2_S_2_O_8_, and visible‐light irradiation in the photoreactor (Figure 6b). In contrast, in the absence of visible‐light irradiation no cellular labeling was detected (Figure 6b). Similarly, removing (NH_4_)_2_S_2_O_8_ or both (NH_4_)_2_S_2_O_8_ and Ru(bpy)_3_^2+^ also led to minimal cellular labeling (Figure 6b). These results clearly show that the photoreactor is also capable of inducing labeling of live cells for downstream analysis and can allow for the screening of other light‐activated chemistry reactions in live‐cell systems. To our knowledge, this photoreactor represents the first uniform reactor for protein or cellular labeling.

## Conclusion

The use of visible light as a reagent in photocatalysis to generate reactive species in a controlled manner provides unique advantages for profiling biological environments due to the highly tunable temporal and spatial precision of light. Thus, for photocatalysis to bridge the gap from chemistry‐centric methods towards enabling biological investigation, a uniform photoreactor that is biocompatible and permits the use of cell culture plates and microcentrifuge tubes with efficient light distribution and low heat generation is a required piece of the photochemistry toolbox.

Herein, we have disclosed the design and development of a multifunctional photoreactor that can be broadly used for visible‐light‐driven activation and catalytic reactions for proteins and live cells. The photoreactor LED was designed with heat dissipation sinks to reduce heat while efficiently and uniformly illuminating the reflective reaction chamber. Photocatalytic reactions at multiple visible light wavelengths (blue, green, and red) were easily achieved in the modular photoreactor through interchangeable light chips. The multiplexing capability of the photoreactor enabled high‐throughput library synthesis, as shown through a relevant medicinal chemistry coupling reaction.

In addition to the demonstrated utility for chemical transformations, we showcased protein‐labeling methods as well as photocatalytic biotinylation of live A375 cells for confocal microscopy imaging. To our knowledge, this photoreactor represents the first uniform reactor for protein and live‐cell labeling in biological environments. We anticipate that this will enable the transfer of light‐mediated chemical transformations, and the development of new ones, into cellular environments and tissues.

## Author Contributions

Project was conceived and initiated by M.J.W., R.C.O., and O.O.F. Experiments were designed by N.B.B., M.J.W., R.C.O., and O.O.F. N.B.B., K.A.R., T.R.R., S.W., J.H.T., and K.A.C. performed experiments. E.C.H., L.R.R., and D.J.H. provided insight and direction for experimental design The manuscript was written by N.B.B., R.C.O., and O.O.F. and proofread by all authors.

## Conflict of interest

N.B.B., K.A.R., T.R.R., J.H.T., K.A.C., S. W., E.C.H., L.R.R., D.J.H., R.C.O., O.O.F. are employees of Merck Sharp & Dohme Corp., a subsidiary of Merck & Co., Inc., Kenilworth, NJ, USA. M.J.W. is an employee of Efficiency Aggregators. A patent on the photoreactor has been filed by Efficiency Aggregators.

## Supporting information

As a service to our authors and readers, this journal provides supporting information supplied by the authors. Such materials are peer reviewed and may be re‐organized for online delivery, but are not copy‐edited or typeset. Technical support issues arising from supporting information (other than missing files) should be addressed to the authors.

Supplementary
