# Supplementary material for: Design of a Multiuse Photoreactor To Enable Visible‐Light Photocatalytic Chemical Transformations and Labeling in Live Cells
Source: Chembiochem. 2020 Sep 16;21(24):3555–62. doi: 10.1002/cbic.202000392 (PMC7756335; doi:10.1002/cbic.202000392)
Supplement: Supplementary file 1 — Supplementary [file CBIC-21-3555-s001.pdf]

ChemBioChem

Supporting Information

# Design of a Multiuse Photoreactor To Enable Visible-Light Photocatalytic Chemical Transformations and Labeling in Live Cells

Noah B. Bissonnette, Keun Ah Ryu, Tamara Reyes-Robles, Sharon Wilhelm, Jake H. Tomlinson, Kelly A. Crotty, Erik C. Hett, Lee R. Roberts, Daria J. Hazuda, M. Jared Willis, Rob C. Oslund,* and Olugbeminiyi O. Fadeyi*

## Table of Contents

Supplementary Figures ........ 3

General Information ........ 6

High Throughput Screen ........ 6

General Western Blot Procedure ........ 6

Photoreactor Reaction Position Uniformity Test ........ 7

Internal Temperature Measurements ........ 7

Irradiance Measurements ........ 8

Spectral Output Measurements ........ 8

Light On/Off Experiment ........ 8

Effect of Visible Light Irradiation on Cell Viability ........ 9

Eosin Y Based Biotinylation of Carbonic Anhydrase ........ 9

Ruthenium Based Biotinylation of Carbonic Anhydrase ........ 9

Photoactivated Biotinylation of Carbonic Anhydrase ........ 9

General Cell Culture Methods ........ 10

Confocal Microscopy Imaging of A375 cells ........ 10

Photoreactor ........ 10

Synthesized Compounds ........ 16

Synthesized Compounds (Spectra) ........ 20

References ........ 40

## Supplementary Figures

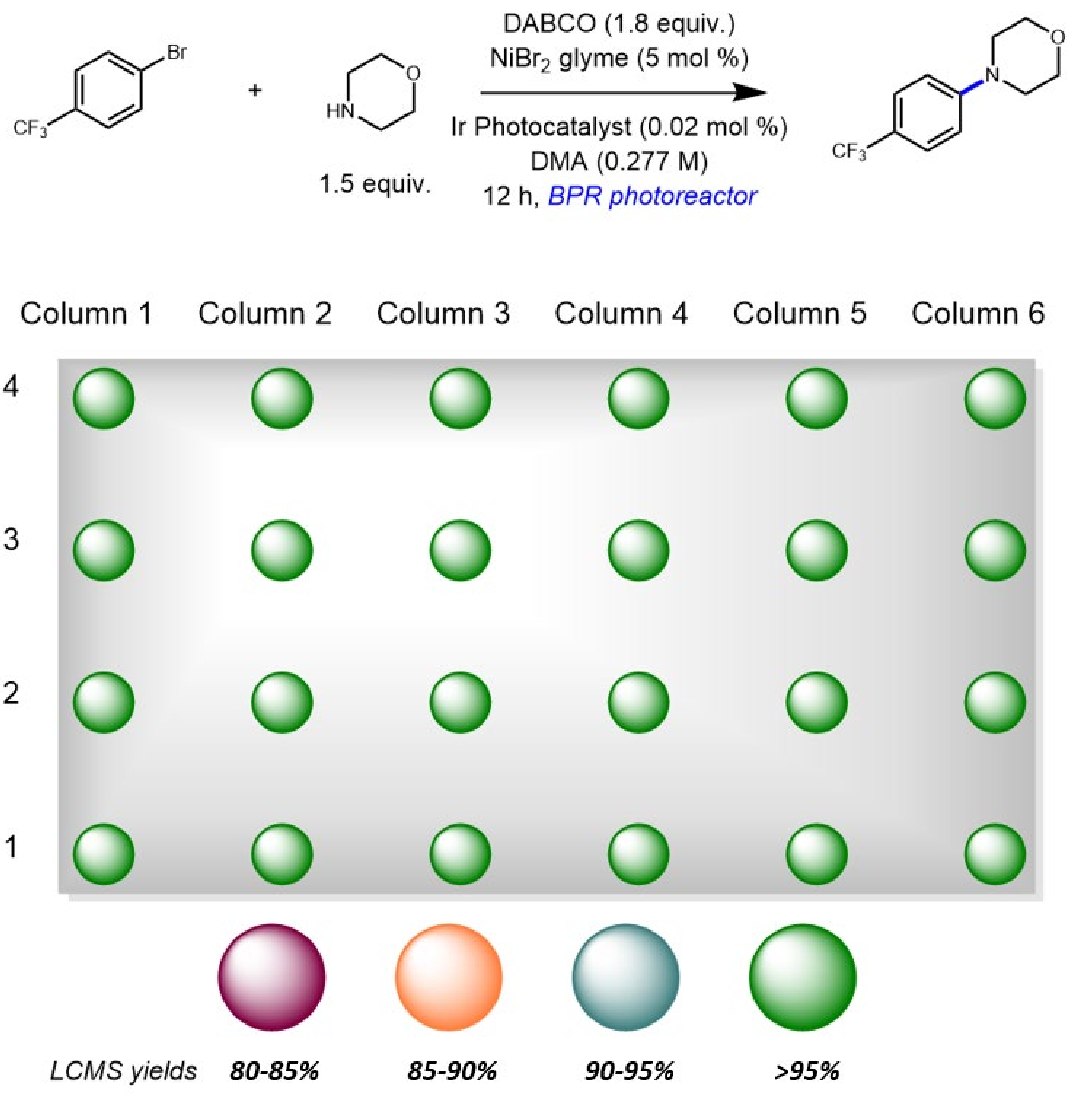


**Figure S1**: Light distribution across multiplex set up. All reactions reached full conversion.

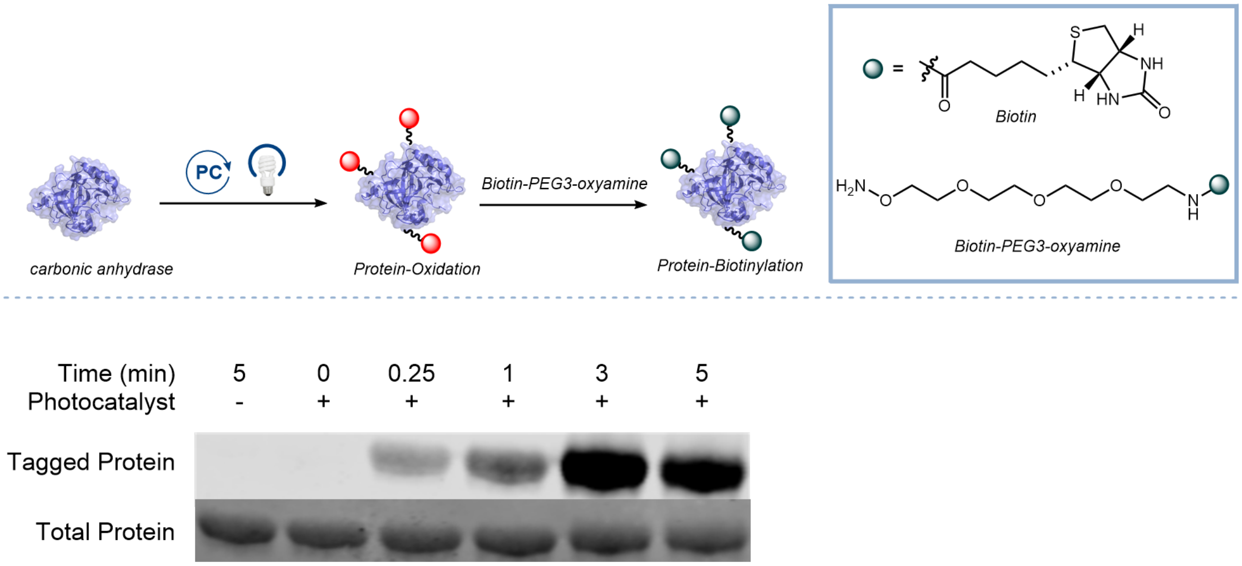


**Figure S2**: Labeling of Carbonic Anhydrase using an Eosin Y photocatalyst and an oxyamine biotin probe.

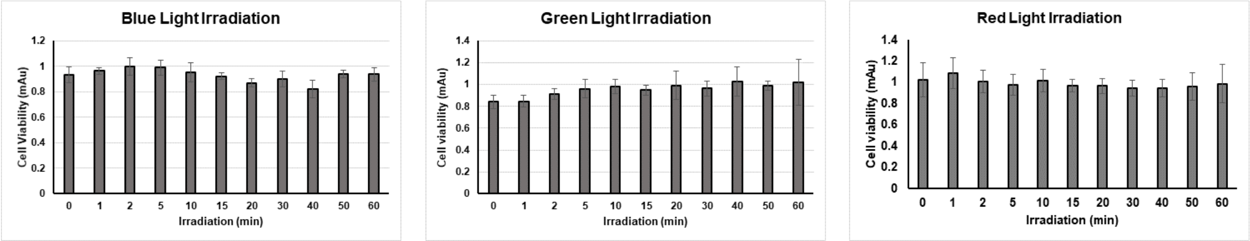


**Figure S3**: A375 cell viability measurements during visible light irradiation over 60 minutes. Cell viability is not altered during light irradiation at any of the tested wavelengths.

## General Information

All reagents were purchased from commercial suppliers and stored per the manufacturer's guidelines. Biotin tyramide (LS-3500.1000) was purchased from Iris Biotech GMBH. BIOTIN-PEG3-OXYAMINE HCL was purchased from Fisher Scientific. Azido-phenol biotin probe ((4-azido-2-hydroxy-N-(6-(6-((3aS,4S,6aR)-2-oxohexahydro-1Hthieno[3,4-d]imidazol-4-yl)hexanamido)hexyl)benzamide)) was synthesized as described in the literature.[1] An Agilent Technologies 1290 Infinity II HPLC attached to an Agilent Technologies 6130 Quadrupole LC/MS using a Supleco Column: Ascentis Express $C_{18}$ HPLC column (5 cm x 2.1 mm x 2.7 μm) was used to collect LCMS data for small molecule compounds. The column was heated to 50°C; the gradient used was 0 min (2% B), 0.2 min (2% B), 1 min (50% B), 1.5 min (98% B), 2 min (98% B). Solvent A was Water and B was MeCN. 0.1% v/v acid modifier (TFA) was added to each solvent. NMR data was acquired using a Bruker 400 Ultrashield NMR. Spectra are internally referenced to residual solvent signals ($CDCl_3$ δ 7.26 ($^1$H) and 77.16 ($^{13}$C) or DMSO δ 2.50 ($^1$H) and 39.52 ($^{13}$C)). Data for $^1$H NMR are reported according to the following conventions: chemical shift (ppm), multiplicity (s = singlet, d = doublet, t = triplet, m = multiplet, q = quintet, br = broad, and combinations thereof), coupling constant J (Hz), integration. Magnetic stirring was induced when specified by placing a V&P Scientific Inc. Magnetic Tumble Stirrer VP 710 Series inside of the photoreactor.

## High Throughput Screen

Three unique amine reactant stock solutions were prepared. Amine (4.5 equiv., 1.247 mmol), DABCO (5.4 equiv, 1.496 mmol) and DMA (2 mL) was added to a vial. A solution of $(Ir[dF(CF_3)ppy]_2(dtbpy))PF_6$ (.0006 equiv) in DMA (4.5 μL) was added to the vial. A solution of NiBr2•glyme (.15 equiv) in DMA (1 mL) was also added to the vial. The vial was placed under an atmosphere of nitrogen, cooled to –78 °C, degassed via vacuum evacuation (3 min), backfilled with nitrogen, and warmed to room temperature. This process was repeated twice. To the vials was then added aryl bromide (0.277 mmol, 1 equiv). The vials were sealed, placed under vacuum for 30 seconds and backfilled with nitrogen 2x. Next 1mL of each amine reactant stock solution was added across the three unique aryl bromide vials under nitrogen. The 8 unique vials were sealed with parafilm, placed in the photoreactor and irradiated at 100% intensity with blue light. After 12 h, each reaction was analyzed by LCMS. Based on LCMS data reactions were either purified via mass directed reversed phase chromatography or discarded. Pure fractions were combined, dried and analyzed using NMR and LCMS.

## General Western Blot Procedure

50 μL of Biorad 4x Laemmli loading buffer was added to each sample and incubated at 95°C for 10 min. During this time, a 12% Criterion TGX precast gel, (18 well, 30 μL, 1 mm. ) was loaded into a gel box and filled with tris-glycine buffer. 10 μL of sample or molecular weight marker was added to each lane (Invitrogen iBright prestained protein ladder). The gel was run at 180V for 45 minutes. Afterwards, the gel was transferred onto a membrane (Invitrogen iBlot 2 PVDF) using the iBlot 2 device according to manufacturer's instructions. The membrane was incubated with 50 mL of blocking solution (TBS-Tween containing 3% BSA) for one hour then 10 μL of IR Dye 800 CW Streptavidin antibody (LI-COR) was added, and the membrane was incubated overnight. The membrane was then washed with TBS-Tween 3x, followed by one wash with Milli-Q water. The membrane was imaged using the LI-COR Odyssey CLx. Afterwards, the membrane was stained with LI-COR Revert Total Protein Stain and incubated for 5-10 minutes. The membrane was then imaged again on the LI-COR.

## Photoreactor Reaction Position Uniformity Test

To a solution of 4-bromobenzotrifluoride (460 µL, 3.328 mmol, 1.0 equiv), morpholine (460 µL, 4.928 mmol, 1.5 equiv), and DABCO (664 mg, 5.936 mmol, 1.8 equiv) in DMA (6 mL) was added $(Ir[dF(CF_3)ppy]_2(dtbpy))PF_6$ (0.732 mg, 0.0002 equiv) as a solution in DMA (26.4 µL). A solution of NiBr2•glyme (36 mg, 0.164 mmol, 0.05 equiv) in DMA (6 mL), which had been sonicated for 1 min, was then added. The vial was placed under an atmosphere of nitrogen, cooled to –78 °C, degassed via vacuum evacuation (3 min), backfilled with nitrogen, and warmed to room temperature. This process was repeated two times. Next a 1-dram vial was placed under vacuum for 30 seconds and backfilled with nitrogen. This process was repeated once. 0.45 mL of the stock solution was added to the vial and sealed with parafilm. This was repeated 23 more times. All the vials were placed in the photoreactor and irradiated at 100% intensity using blue light. After 12 h, LCMS was used to analyze % conversion (at 254.4 nm) of each vial and a map of light coverage was generated based on conversion.

| | Column 1 | Column 2 | Column 3 | Column 4 |
|---|---|---|---|---|
| Row 6 | 100% Conversion | 100% Conversion | 100% Conversion | 100% Conversion |
| Row 5 | 100% Conversion | 100% Conversion | 100% Conversion | 100% Conversion |
| Row 4 | 100% Conversion | 100% Conversion | 100% Conversion | 100% Conversion |
| Row 3 | 100% Conversion | 100% Conversion | 100% Conversion | 100% Conversion |
| Row 2 | 100% Conversion | 100% Conversion | 100% Conversion | 100% Conversion |
| Row 1 | 100% Conversion | 100% Conversion | 100% Conversion | 100% Conversion |

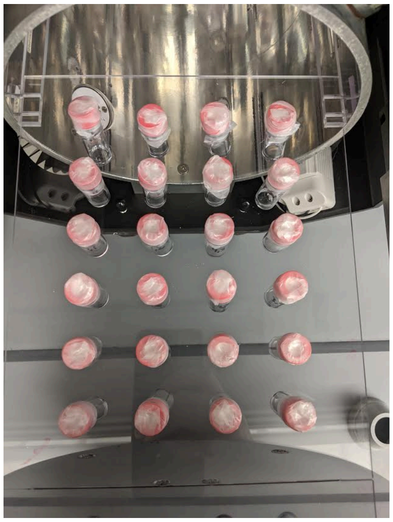

**Figure S4**: Left, table showing percent conversion at each position in the photoreactor. Right, image of reaction tray to highlight positioning of each vial.

## Internal Temperature Measurements

Thermal data was collected using the OM-CP-RHTemp101A Humidity and Temperature Data Logger with a thermocouple. Data was processed using the Omega Software. Temperature was recorded every 15 seconds for 18 hours for the Biophotoreactor with Blue, Green and Red LEDs. Operating temperature after heating period was determined by averaging temperature data points from 2 to 18 hours. Data was exported to MS Excel and plotted (see Figure 3e).

## Irradiance Measurements

Irradiance measurements were obtained using an Ocean Optics Flame-S-UV-VIS-spectrometer (Ocean Optics Inc., Largo, FL) outfitted with an INTSMA-005 slit, a QP400-1-UV-VIS fiber cable and a CC-3-UV-S cosine corrector that was pre-calibrated at the manufacturing site. Briefly, one LED was connected to the photoreactor and the remaining 3 LEDs were removed and each LED port was covered to inhibit excess background light into the reactor. The cosine corrector probe was positioned 10.5 cm from the LED. The irradiance was measured by increasing the photoreactor light power % gradually (10, 20, 40, 60, 80, 100%) with the fan on (100%). The LED was turned on at each power and equilibrated for 5 minutes before recording the irradiance. Measurement readings were taken using the Oceanview software following the manufacturer's instructions. The background spectrum was measured with the LED turned off and the door of the photoreactor closed. The calibration file specific to the instrument (provided by the manufacturer) was used for measurements.

Acquisition parameters include:

a. Integration time: 100 ms

b. Scans to Average: 10

c. Boxcar Width: 5

d. Electric Dark: enabled

## Spectral Output Measurements

Emission spectra of LED were measured with the UPRtek MK350N Premium handheld spectrometer. First, dark calibration was performed following spectrometer "dark calibration" setting. Measurement of spectra was performed by selecting the "spectrum" setting (see Detailed settings below). The spectrometer was directed towards the center of the photoreactor operating at 100% light intensity. The measurement was taken 6 inches from the front of the inner chamber with the lid open. Data was recorded and transferred to Microsoft Excel for processing (see Figure S5).

**Detailed settings:**
Default spectrum setting:
integration mode: 100 us
long time exposure: off
sampling time: 64K
flicker resolution: 2.0 Hz
frequency domain (start): 1Hz
Frequency domain (end): 300 Hz
time domain start: 0 s
time domain end: 0.25 s

## Light On/Off Experiment

600 µL of Carbonic Anhydrase (1 mg/mL) in PBS and 6 µL of biotin azide tag (25 mM) in DMSO were added to a microcentrifuge tube. The sample was irradiated at 100% intensity in the photoreacto with blue LED light for 5 minutes and afterwards left in the dark for 5 minutes. This process was repeated 4x. After each light or dark period, 60 µL aliquots were taken. 15 µL of loading buffer was added to each aliquot. The

samples were then analyzed through western blot analysis. Densitometry was used to quantify biotinylated protein levels. Figure 5d shows average data from three independent experiments with error bars representing ± S.D.

## Effect of Visible Light Irradiation on Cell Viability

Cell viability was measured with the CyQUANT MTT Cell proliferation Assay kit (V13154) following the manufacturer's guidelines. Briefly, A375 cells were plated in 96 well plates at a density of 50k cells/well and grown overnight. The cells were irradiated in the corresponding wavelength of light for periods between 0 to 60 min in the photoreactor at 100% intensity. Following irradiation, the culture medium was removed and replaced with 100 µL of fresh PBS + 10% HI FBS. 10 µL of 12 mM MTT stock solution was then added to each well. The plate was incubated at 37°C for 4 hours. Following incubation, all but 25 µL of medium was removed from the wells. 50 µL of DMSO was added to each well and mixed thoroughly with a pipette, avoiding bubbles. The plate was incubated at 37°C for 10 minutes. Each well was mixed thoroughly again, and the absorbance was measured at 540 nm.

## Eosin Y Based Biotinylation of Carbonic Anhydrase

200 µL of Carbonic Anhydrase (1 mg/mL) in PBS, 2 µL of Eosin Y (500 µM) in PBS and 2 µL of biotin tag (25 mM) in DMSO were added to a microcentrifuge tube. This was repeated 4 times. One sample was prepared without Eosin Y as a control. Samples were irradiated at 100% intensity with blue light in the photoreactor with max fan for 0 sec, 15 sec, 1 min, 3 min and 5 min, respectively. The sample without photocatalyst was irradiated for 5 min. The samples were then analyzed through western blot using the General Western Blot Procedure.

## Ruthenium Based Biotinylation of Carbonic Anhydrase

200 µL of Carbonic Anhydrase (1 mg/mL) in PBS, 2 µL of $Ru(bpy)_3(PF_6)_2$ (100 µM) in DMSO, 2 µL of ammonium persulfate (25 mM) in PBS and 2 µL of biotin phenol tag (25 mM) in DMSO were added to a microcentrifuge tube. Samples were irradiated at 100% intensity with blue light in the photoreactor with max fan for 0 sec, 30 sec, 2 min, 7.5 min and 15 min respectively. The samples were then analyzed through western blot using the General Western Blot Procedure.

## Photoactivated Biotinylation of Carbonic Anhydrase

200 µL of Carbonic Anhydrase (1 mg/mL) in PBS and 2 µL of biotin azide tag (25 mM) in DMSO were added to a microcentrifuge tube. Samples were irradiated at 100% intensity with blue light in the photoreactor with max fan for 0 sec, 5 min, 10 min, 20 min and 40 min respectively. The samples were then analyzed through western blot using the General Western Blot Procedure.

## General Cell Culture Methods

A375 cells were purchased from Sigma-Aldrich (88113005-1VL) and cultured in 1x DMEM with GlutaMAX-I (Thermo Fisher Scientific: 10569-010) containing 10% HI FBS (Thermo Fisher Scientific: 10082-139), and 100 IU Penicillin/100µg/mL Streptomycin (Thermo Fisher Scientific: 15140-148). Cells were grown at 37°C with 5% $CO_2$ in tissue culture dishes. For passaging, cells were washed once with 1x DPBS and suspended using TrypLE Express Enzyme (1x) (Gibco: 12604021).

## Confocal Microscopy Imaging of A375 cells

µ-Dish 35 mm, glass bottom dishes (ibidi: 81158) were rinsed 1x with 1 mL of 1x DPBS (Gibco: 14190144) and 1 mL of poly-L-lysine solution (Sigma: P4707-50ML) was added per dish and incubated for 30 min at room temperature. Dishes were washed 2x with 1 mL of 1x DPBS and 500,000 A375 cells were seeded in 400 µL of A375 culture media (see General Cell Culture Methods section) and incubated overnight at 37°C with 5% $CO_2$.

Reaction solutions were prepared in 1 mL of 1x DPBS at the following concentrations: 10 µM $Ru(ppy)_3$, 250 µM biotin phenol, 250 µM ammonium persulfate. Cell culture media was removed using a pipette and reaction solution was added. Samples were irradiated at 100% intensity in the photoreactor (or stored under tinfoil for the no light control) for 30 minutes. Afterwards the reaction mixture was removed and 1 mL of 1x DPBS was added using a pipette.

Cells were washed 1x with 1x DPBS and staining procedure for imaging was modified as reported previously.[1] Briefly, 6% paraformaldehyde (PFA, Electron Microscopy Sciences: 15710) and 0.2% glutaraldehyde (Sigma-Aldrich: G5882-10X10ML) were prepared in 1x DPBS and added gently at equal ratios per dish (final concentration of 3% PFA and 0.1% glutaraldehyde in a total volume of 400 µL) and incubated for 10 min at 4°C. The dishes were washed 3x in Stain Buffer (BD Biosciences: 554656) and incubated overnight in 1 mL of Stain Buffer at 4°C. The following day, samples were stained with Alexa Fluor 488 Streptavidin (BioLegend: 405235) at a 1:200 dilution in 400 µL of Stain Buffer and incubated overnight at 4°C. The samples were washed 1x with 1 mL of Stain Buffer and Hoechst DNA dye (Cayman Chemical Company: 600332) was added at a 1:10,000 dilution in 400 µL of Stain Buffer per dish and incubated while protected from light for 10 min at room temperature. The dishes were washed 2x in Stain Buffer and fixed with 400 µL of a 3% PFA and 0.1% Glutaraldehyde solution in 1x DPBS for 5 min at room temperature, washed 2x in 1 mL of Stain Buffer, and imaged using a Zeiss LSM800 inverted, confocal microscope using a 63X oil immersion objective.

## Photoreactor

The photoreactor (BPR200, Fisher, Product number: NC1558343 or available through Sigma-Aldrich) was designed, developed, and manufactured by Efficiency Aggregators (Richmond, TX, USA). Caution: protective eyewear (i.e. orange UV protective eyewear) should be worn during light box operation.

The figures below highlight components and features of the photoreactor described in the manuscript.

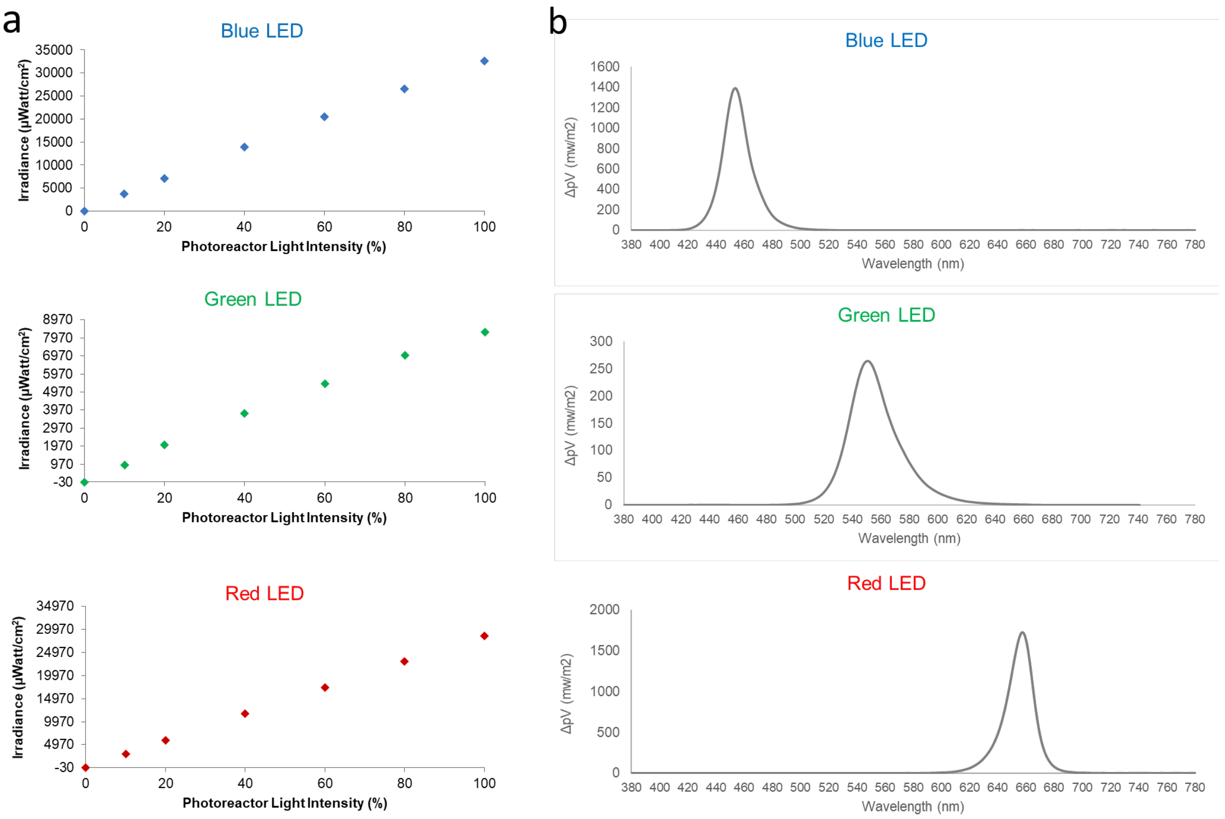


**Figure S5:** Panel a, irradiance measurements (µWatt/cm$^2$) for the blue, green, and red LED light chips used in the photoreactor. Irradiance was measured 10.5 cm from a single photoreactor LED chip at 0, 10, 20, 40, 60, 80, or 100% photoreactor intensity. Panel b, spectral output measurements of the blue, green, and red LED light chips used in the photoreactor.

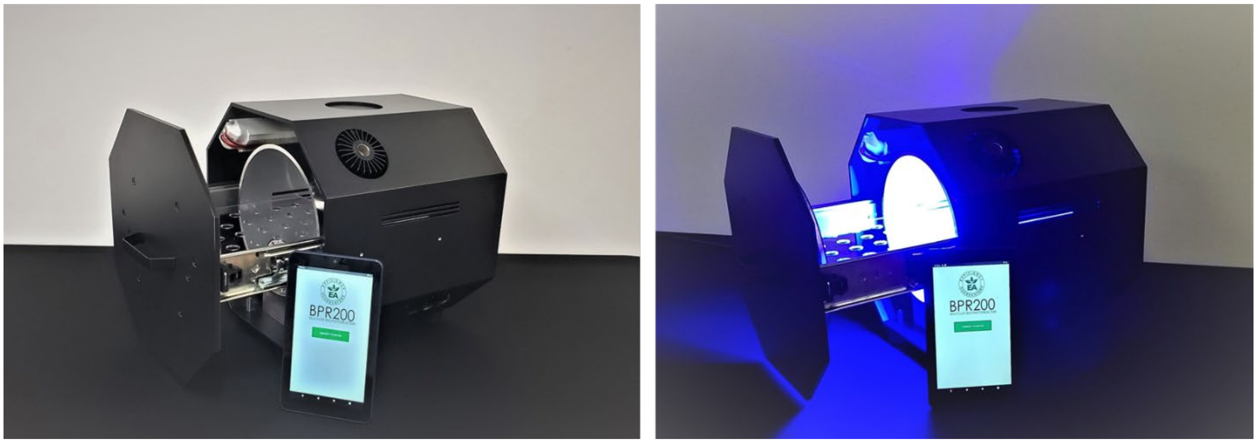


**Figure S6:** Left, side view of photoreactor with control tablet (light off). Right, side view of photoreactor with control tablet (light on).

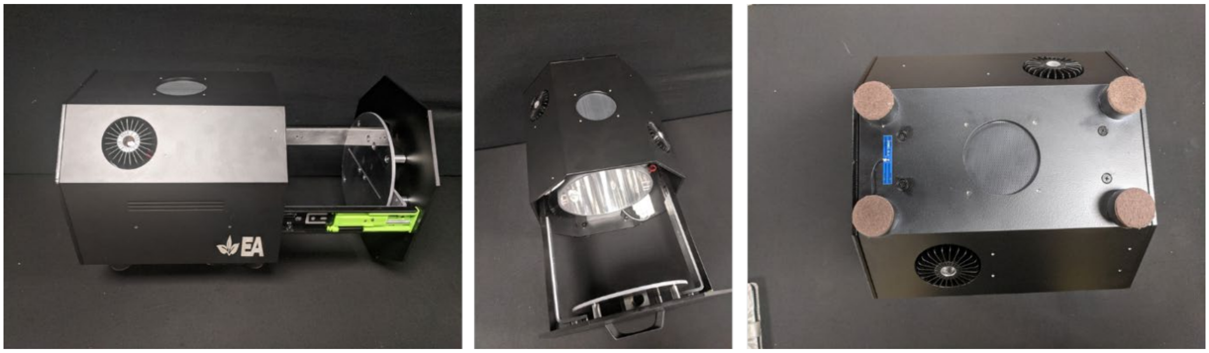


**Figure S7:** Left, side view of photoreactor outer chamber with lid open. Middle, top view of photoreactor with lid open. Right, bottom view of photoreactor.

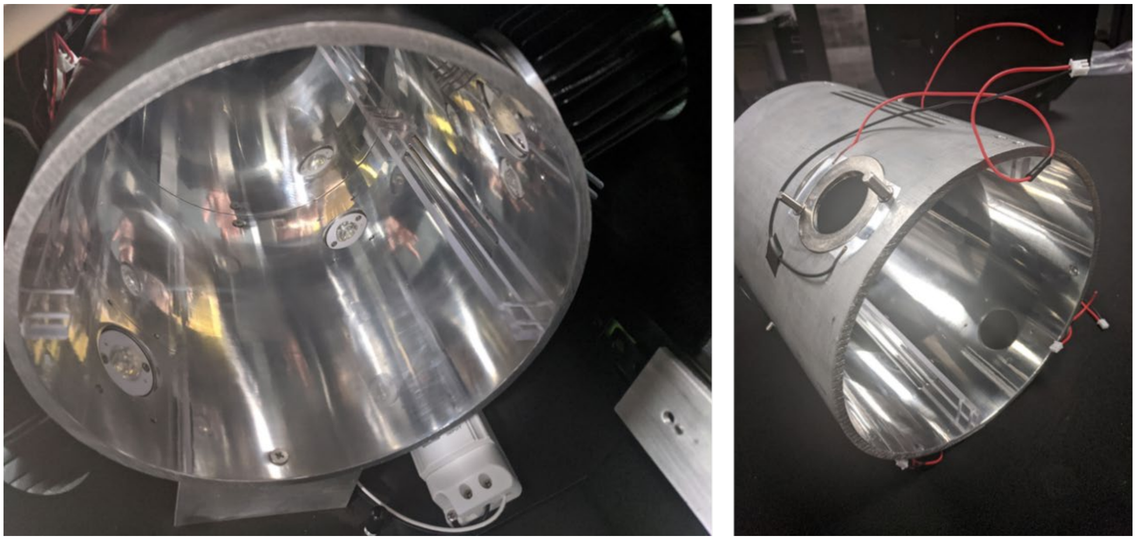

**Figure S8:** Left, photoreactor inner chamber with reflective interior. Right, outside of inner chamber showing contact pins where LED chips connect.

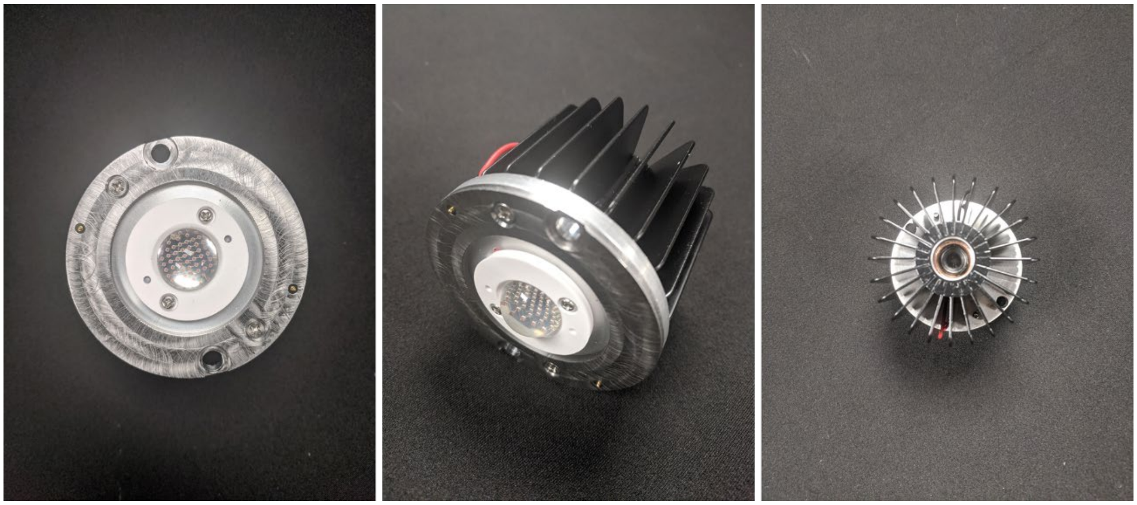

**Figure S9:** Left, front view of LED Chip. Middle, side view of LED chip showing attached heat sink. Right, back view of LED chip showing heat sink core and fin. Each chip was fabricated at 20W/chip, 29.7 lm/W at 455nm, 99.5 lm/W at 555 nm, and 24.5 lm/W at 660 nm.

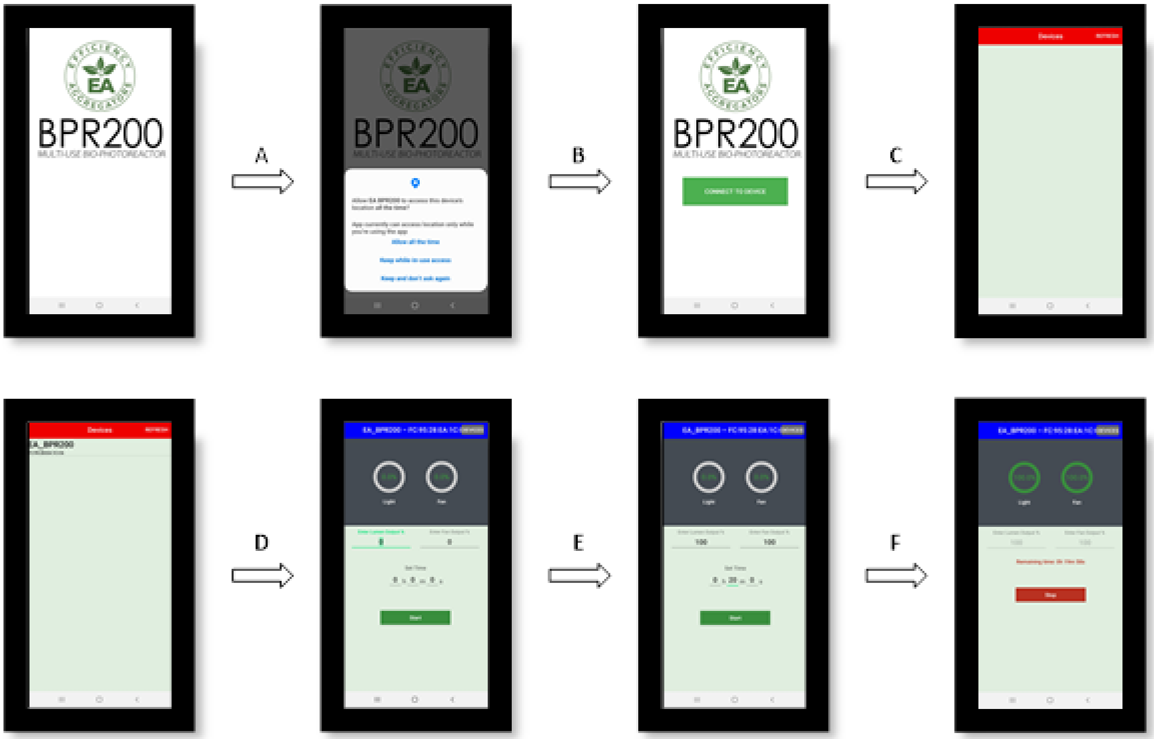


**Figure S10.** Photoreactor control tablet and instructions on how to turn on the photoreactor. A) Opening the BPR app for the first time will launch a screen asking for various permissions. B) Upon making a choice, the main screen will be displayed. C) Click connect device and the device management screen will pop up. If the photoreactor is plugged in and on, its name should appear on screen after hitting the refresh button. D) Selecting the photoreactor will launch the control screen. E) Input the chosen fan and light intensity and select a reaction time. F) Hit start and irradiation will begin.

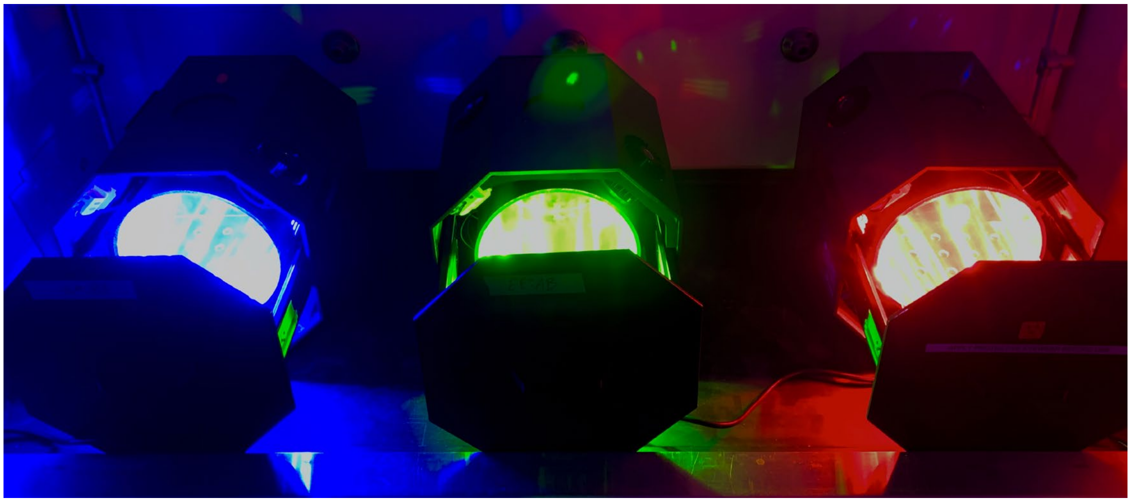

**Figure S11:** Illuminated photoreactor displaying blue (455nm), green (555nm), and red (660nm) LED light chips at 100% intensity.

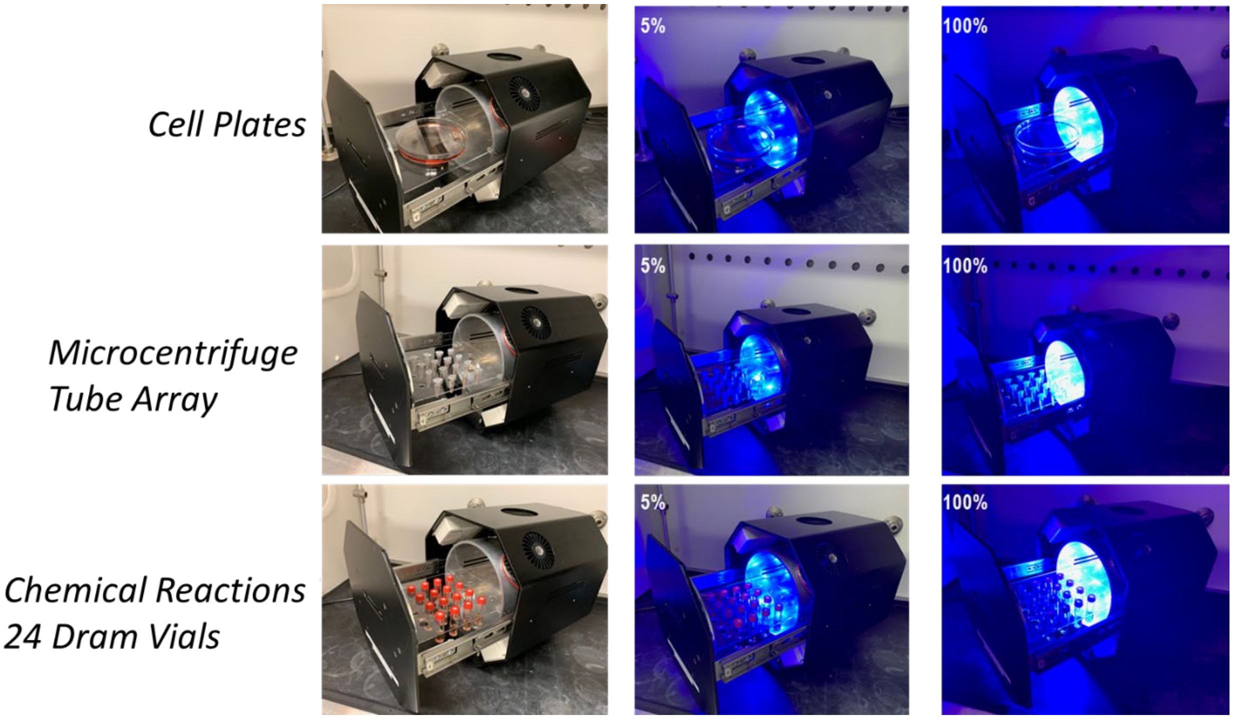


**Figure S12:** Open Photoreactor loaded with vials, microcentrifuge tubes and cell plates at various light intensities.

## Synthesized Compounds

**4-(4-(trifluoromethyl)phenyl)morpholine (4)**

To a solution of 4-bromobenzotrifluoride (115 µL, 0.832 mmol, 1.0 equiv), morpholine (115 µL, 1.232 mmol, 1.5 equiv), and DABCO (166 mg, 1.484 mmol, 1.8 equiv) in DMA (1.5 mL) was added (Ir[dF($CF_3$)ppy]$_2$(dtbpy))$PF_6$ (0.183 mg, 0.0002 equiv) as a solution in DMA (6.6 µL). A solution of NiBr2•glyme (9 mg, 0.041 mmol, 0.05 equiv) in DMA (1.5 mL), which had been sonicated for 1 min, was then added. The vial was placed under an atmosphere of nitrogen, cooled to –78 °C, degassed via vacuum evacuation (3 min), backfilled with nitrogen, and warmed to room temperature. This process was repeated two times, and the reaction vial was then sealed with parafilm, placed in the photoreactor, and irradiated at 100% intensity. After 12 h, the reaction mixture was analyzed using LCMS (100% conversion). The mixture was then purified through reversed phase chromatography furnishing product in a 68% yield. Spectra matches known compound.[2] **$^1$H NMR (400 MHz, $CDCl_3$):** δ 7.53 (d, *J* = 8.7 Hz, 2H), 6.97 (d, *J* = 8.7 Hz, 2H), 3.90 (t, *J* = 4.9 Hz, 4H), 3.27 (t, *J* = 4.9 Hz, 4H). **LCMS:** Expected mass for $C_{11}H_{13}F_3NO$ [M+H]=232.1. Found=232.1.

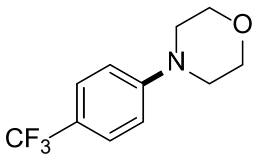


**Acetophenone (7)**

To a vial charged with a stir bar was added 2-bromo-1-phenylethan-1-one (100 mg, 0.5 mmol, 1 equiv), diethyl 2,6-dimethyl-1,4-dihydropyridine-3,5-dicarboxylate (139.9 mg, 0.55 mmol, 1.1 equiv), and Eosin Y (8.7 mg, 0.0125 mmol, 0.025 equiv). The vial was placed under vacuum and backfilled with nitrogen. This was repeated 2x. In a separate vial, DMF (2 mL, 0.25 M) was degassed via bubbling nitrogen through for 10 min. The degassed DMF was added to the other vial under nitrogen. Additionally, DIPEA (175 µL, 1 mmol, 2 equiv) was added. The top of the vial was sealed with parafilm, placed in the photoreactor, and irradiated at 100% intensity with green light. After 18 h, the reaction mixture was analyzed through TLC (Eulent Hex/EtOAc 85:15). TLC indicated 100% conversion of starting material to product. The reaction mixture was then transferred to a separatory funnel and extracted from water with DCM 3x. The combined organic layers were dried with magnesium sulfate and concentrated under reduced pressure. The crude residue was purified through normal phase chromatography (Hex/EtOAc 100:0 to 80:20). Pure fractions were identified through TLC, combined and concentrated under reduced pressure yielding acetophenone.[3] **$^1$H NMR (400 MHz, Chloroform-*d*)** δ 7.98 – 7.94 (m, 2H), 7.60 – 7.53 (m, 1H), 7.50 – 7.43 (m, 2H), 2.61 (s, 3H). Spectra matches known compound.[4]

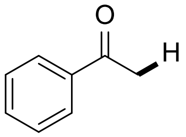

**(S)-2-acetamido-N-methyl-3-(2-(perfluoropropan-2-yl)-1H-indol-3-yl)propenamide (11)**

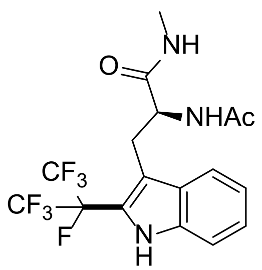


To a vial charged with a stir bar was added (S)-2-acetamido-3-(1H-indol-3-yl)-N-methylpropanamide (63.1 mg, 0.3 mmol, 1 equiv), ascorbic acid (79.3 mg, 0.45 mmol, 1.5 equiv), 2,4,6-collidine (59 µL, 0.45 mmol, 1.5 equiv), Zinc Phthalocyanine (1.73 mg, 0.003 mmol, 1 mol%) and MeCN/DMF (1:1, 0.12 M). Nitrogen was bubbled through the mixture for 15 min. Next, heptafluoro-2-iodopropane (128 µL, 0.9 mmol, 3 equiv) was added and nitrogen was bubble through the mixture for 3 min. Afterwards, the septum of the reaction vial was wrapped in parafilm. The mixture was then irradiated at 100% intensity with red LED light in the photoreactor and stirred for 22 hours. The mixture was extracted from brine using DCM (3X). The combined organic fractions were dried over magnesium sulfate and concentrated under reduced pressure.[5] The crude residue was purified through normal phase chromatography (DCM/MeOH, 100:0 to 90:10 gradient). The pure fractions were combined and concentrated under reduced pressure furnishing (S)-2-acetamido-N-methyl-3-(2-(perfluoropropan-2-yl)-1H-indol-3-yl)propenamide (102.0 mg, 80% yield). **$^1$H NMR (400 MHz, Acetonitrile-*d*$_3$)** δ 9.84 (s, 1H), 7.82 (d, *J* = 8.1 Hz, 1H), 7.50 (d, *J* = 8.3 Hz, 1H), 7.29 (ddd, *J* = 8.2, 7.1, 1.1 Hz, 1H), 7.17 (td, *J* = 7.6, 7.1, 0.9 Hz, 1H), 6.85 (d, *J* = 8.4 Hz, 1H), 6.57 – 6.51 (m, 1H), 4.56 (q, *J* = 7.3 Hz, 1H), 3.39 (ddd, *J* = 14.4, 6.5, 2.5 Hz, 1H), 3.20 – 3.10 (m, 1H), 2.56 (d, *J* = 4.7 Hz, 3H), 1.78 (s, 3H). **$^{13}$C NMR (101 MHz, $CD_3CN$)** δ 172.32, 170.66, 138.05, 128.64, 125.46, 121.31, 120.94, 118.33, 117.30, 112.90, 93.75-92.73 (m), 92.09-91.07 (m), 55.41, 39.94 (dq, *J* = 39.7, 20.7, 19.4 Hz), 28.33 (d, 7.3 Hz), 26.22, 22.95. **$^{19}$F NMR (376 MHz, Acetonitrile-*d*$_3$)** δ -76.52 (p, *J* = 9.1 Hz, $CF_3$), -76.75 (p, *J* = 9.0 Hz, $CF_3$), -184.11 (hept, *J* = 8.5 Hz, $CR_2F$). **LCMS:** Expected mass for $C_{17}H_{17}F_7N_3O_2$ [M+H]=428.1. Found=428.1.

**3-(5-morpholinopyridin-2-yl)oxetane-3-carbonitrile (12)**

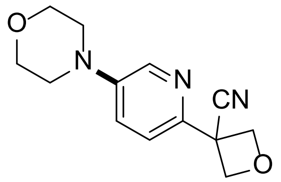


Synthesized using the High Throughput Screen Procedure. 97% conversion, 66% yield. **$^1$H NMR (400 MHz, DMSO-*d*$_6$):** δ 8.39 (d, *J* = 2.8 Hz, 1H), 7.51 (d, *J* = 8.7 Hz, 1H), 7.44 (dd, *J* = 8.8, 3.0 Hz, 1H), 5.07 (d, *J* = 6.3 Hz, 2H), 4.98 (d, *J* = 6.3 Hz, 2H), 3.79 – 3.72 (m, 4H), 3.25 – 3.18 (m, 4H). **$^{13}$C NMR (101 MHz, DMSO):** δ 146.45, 143.64, 136.88, 122.41, 121.15, 120.78, 78.33 (2C), 65.80 (2C), 47.37 (2C), 41.80. **LCMS:** Expected mass for $C_{13}H_{16}N_3O_2$ [M+H]=246.1. Found= 246.2.

**4-(1-(phenylsulfonyl)-1H-pyrrolo[2,3-b]pyridin-5-yl)morpholine (13)**

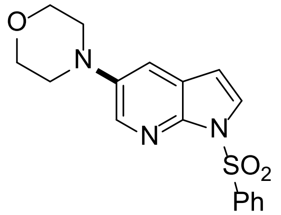


Synthesized using the High Throughput Screen Procedure. 52% conversion, 35% yield. **$^1$H NMR (400 MHz, Chloroform-*d*)** δ 8.23 (d, *J* = 2.6 Hz, 1H), 8.16 – 8.12 (m, 2H), 7.66 (d, *J* = 4.0 Hz, 1H), 7.60 – 7.51 (m, 1H), 7.46 (td, *J* = 7.1, 1.5 Hz, 2H), 7.39 (d, *J* = 2.2 Hz, 1H), 6.51 (d, *J* = 4.0 Hz, 1H), 3.93 – 3.86 (m, 4H), 3.18 – 3.11 (m, 4H). **$^{13}$C NMR (101 MHz, $CDCl_3$)** δ 143.88, 142.85, 138.54, 137.06, 134.06, 129.13 (2C), 127.94 (2C), 127.48, 123.28, 117.02, 105.61, 66.63 (2C), 51.20 (2C). **LCMS:** Expected mass for $C_{17}H_{18}N_3O_3S$ [M+H]=344.1. Found= 344.1

**4-methyl-3-morpholinobenzonitrile (14)**

Synthesized using the High Throughput Screen Procedure. 68% conversion, 9% yield. **$^{1}$H NMR (400 MHz, Chloroform-*d*)** δ 7.32 (dd, *J* = 7.8, 1.4 Hz, 1H), 7.30 – 7.26 (m, 2H), 3.96 – 3.89 (m, 4H), 3.00 – 2.93 (m, 4H), 2.39 (s, 3H). **$^{13}$C NMR (101 MHz, $CDCl_3$)** δ 150.86, 138.69, 132.31, 127.63, 122.71, 118.85, 110.47, 66.87 (2C), 52.10 (2C), 18.52. **LCMS:** Expected mass for $C_{12}H_{15}N_2O$ [M+H]=203.1. Found=203.1.

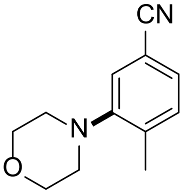

**3-(5-((6-hydroxyspiro[3.3]heptan-2-yl)amino)pyridin-2-yl)oxetane-3-carbonitrile (15)**

Synthesized using the High Throughput Screen Procedure. 86% conversion, 73% yield. **$^{1}$H NMR (400 MHz, Chloroform-*d*)** δ 8.29 (d, *J* = 2.8 Hz, 1H), 7.44 (d, *J* = 8.7 Hz, 1H), 7.02 (dd, *J* = 8.7, 2.8 Hz, 1H), 5.20 (d, *J* = 6.3 Hz, 2H), 5.12 (d, *J* = 6.3 Hz, 2H), 4.31 – 4.20 (m, 1H), 3.85 (p, *J* = 7.6 Hz, 1H), 2.52 (tq, *J* = 11.6, 5.8, 4.9 Hz, 3H), 2.38 (dt, *J* = 12.0, 6.1 Hz, 1H), 2.07 – 1.93 (m, 4H). **$^{13}$C NMR (101 MHz, $CDCl_3$)** δ 143.86, 139.59, 134.48, 122.55, 121.16, 119.98, 79.14 (2C), 69.25, 63.38, 45.71, 45.50, 44.70, 42.70, 42.29, 29.16. **LCMS:** Expected mass for $C_{16}H_{20}N_3O_2$ [M+H]=286.1. Found=286.1

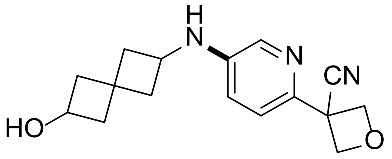

**6-((1-(phenylsulfonyl)-1H-pyrrolo[2,3-b]pyridin-5-yl)amino)spiro[3.3]heptan-2-ol (16)**

Synthesized using the High Throughput Screen Procedure. 43% conversion, 8% yield. **$^{1}$H NMR (400 MHz, DMSO-*d*$_6$)** δ 8.04 – 7.98 (m, 2H), 7.74 (d, *J* = 2.5 Hz, 1H), 7.71 – 7.65 (m, 2H), 7.58 (t, *J* = 7.7 Hz, 2H), 6.96 (d, *J* = 2.2 Hz, 1H), 6.61 (d, *J* = 4.0 Hz, 1H), 3.95 (p, *J* = 7.5 Hz, 2H), 3.67 (p, *J* = 7.5 Hz, 1H), 2.45 – 2.25 (m, 3H), 2.15 (dt, *J* = 11.7, 6.2 Hz, 1H), 1.88 – 1.73 (m, 4H). **$^{13}$C NMR (101 MHz, DMSO)** δ 141.08, 139.68, 137.81, 134.35, 133.23, 129.45 (2C), 127.18 (2C), 126.75, 123.17, 110.01, 106.21, 61.42, 45.73, 45.44, 44.23, 42.23, 41.71, 28.35. **LCMS:** Expected mass for $C_{20}H_{22}N_3O_3S$ [M+H]=384.1. Found= 384.2

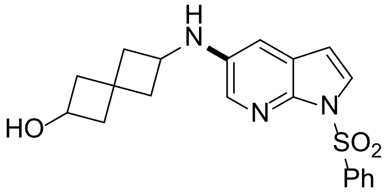

**3-((6-hydroxyspiro[3.3]heptan-2-yl)amino)-4-methylbenzonitrile (17)**

Synthesized using the High Throughput Screen Procedure. 99% conversion, 81% yield. **$^{1}$H NMR (400 MHz, Chloroform-*d*)** δ 7.10 (d, *J* = 7.6 Hz, 1H), 6.97 (dd, *J* = 7.6, 1.4 Hz, 1H), 6.72 – 6.67 (m, 1H), 4.30 – 4.24 (m, 1H), 4.22 (s, 1H), 3.81 (p, *J* = 7.5 Hz, 1H), 2.59 – 2.44 (m, 3H), 2.36 (dt, *J* = 11.4, 5.9 Hz, 1H), 2.17 (s, 3H), 2.08 – 1.88 (m, 4H). **$^{13}$C NMR (101 MHz, $CDCl_3$)** δ 144.71, 130.83, 128.18, 121.80, 119.84, 113.59, 110.58, 63.42, 45.71, 45.42, 45.10, 42.90, 42.45, 29.15, 17.91. **LCMS:** Expected mass for $C_{15}H_{19}N_2O$ [M+H]=243.1. Found=243.1

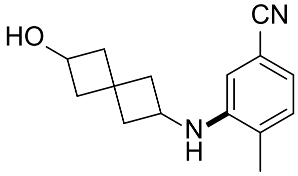

**3-(5-(2-oxa-6-azaspiro[3.3]heptan-6-yl)pyridin-2-yl)oxetane-3-carboxamide (18)**

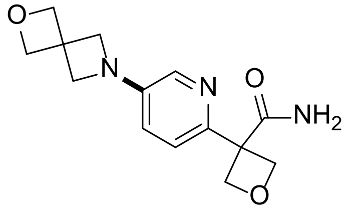

Synthesized using the High Throughput Screen Procedure. Nitrile was hydrolyzed to amide. 70% conversion, 32% yield. **$^1$H NMR (400 MHz, DMSO-*d*$_6$)** δ 7.87 (d, *J* = 2.7 Hz, 1H), 7.42 (d, *J* = 8.5 Hz, 1H), 6.91 (dd, *J* = 8.5, 2.9 Hz, 1H), 5.05 (d, *J* = 6.2 Hz, 2H), 4.96 (d, *J* = 6.2 Hz, 2H), 3.64 (s, 4H), 3.55 (s, 4H). **$^{13}$C NMR (101 MHz, DMSO)** δ 147.08, 141.20, 133.25, 121.32, 120.73, 118.52, 78.43 (2C), 62.91 (2C), 55.40 (2C), 42.09, 41.79. **LCMS:** Expected mass for $C_{14}H_{18}N_3O_3$ [M+H]=276.1. Found=276.1

**4-methyl-3-(2-oxa-6-azaspiro[3.3]heptan-6-yl)benzonitrile (19)**

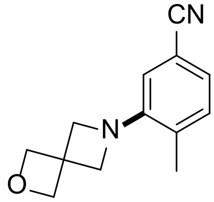

Synthesized using the High Throughput Screen Procedure. 71% conversion, 7% yield**. $^1$H NMR (400 MHz, Chloroform-*d*)** δ 7.12 – 7.06 (m, 2H), 6.70 (s, 1H), 4.86 (s, 4H), 4.13 (s, 4H), 2.28 (s, 3H). **$^{13}$C NMR (101 MHz, CDCl$_3$)** δ 132.21, 127.60 (weak signal), 123.62, 121.74 (weak signal), 119.53, 116.08, 110.35, 81.12 (2C), 62.92 (2C), 38.87, 19.92. **LCMS:** Expected mass for $C_{13}H_{15}N_2O$ [M+H]=215.1. Found= 215.1

Synthesized Compounds (Spectra)

**4-(4-(trifluoromethyl)phenyl)morpholine (4)**

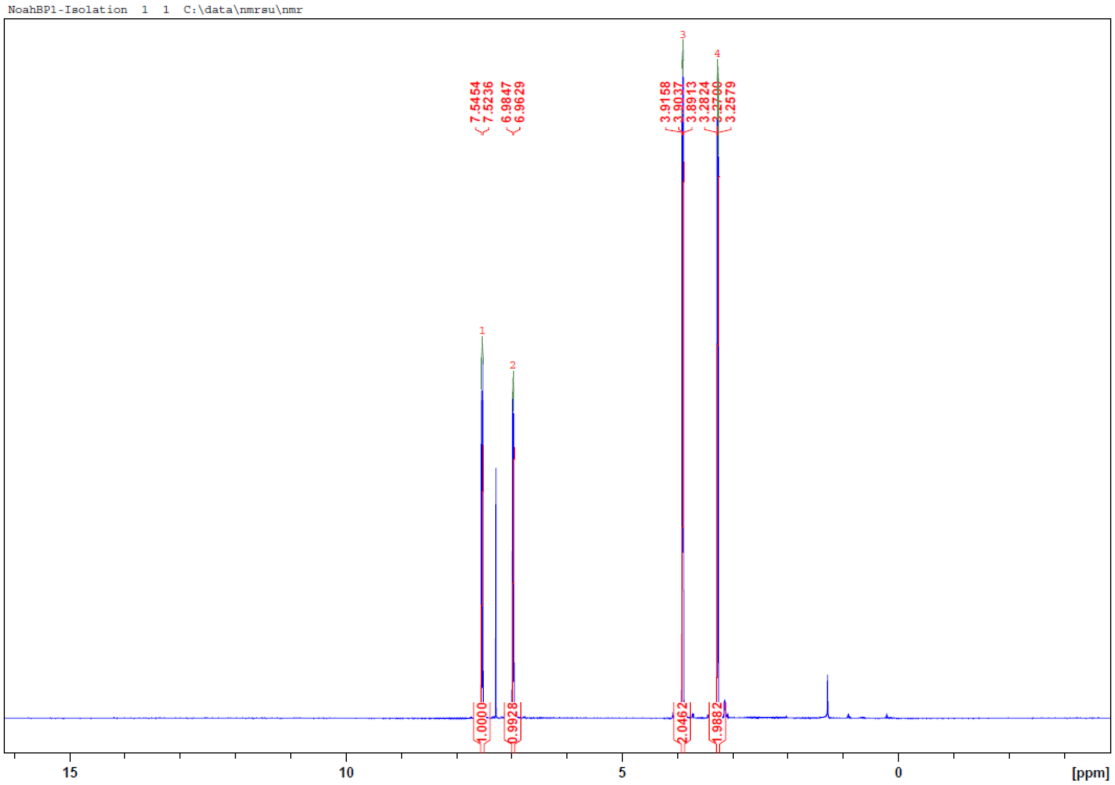


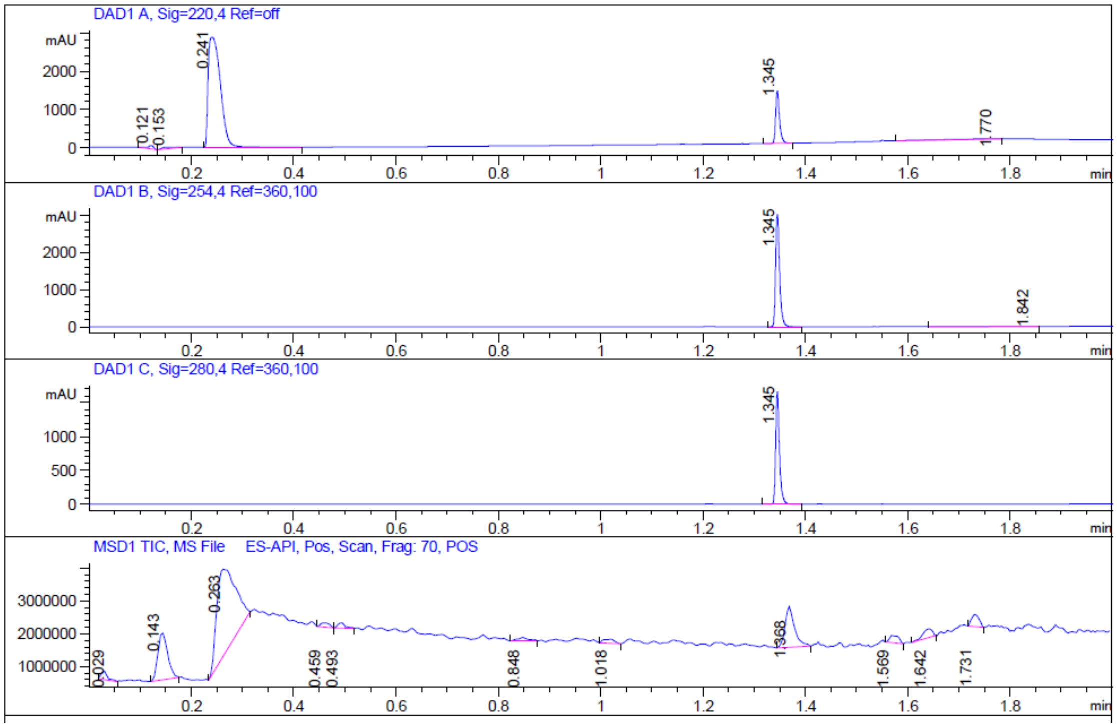

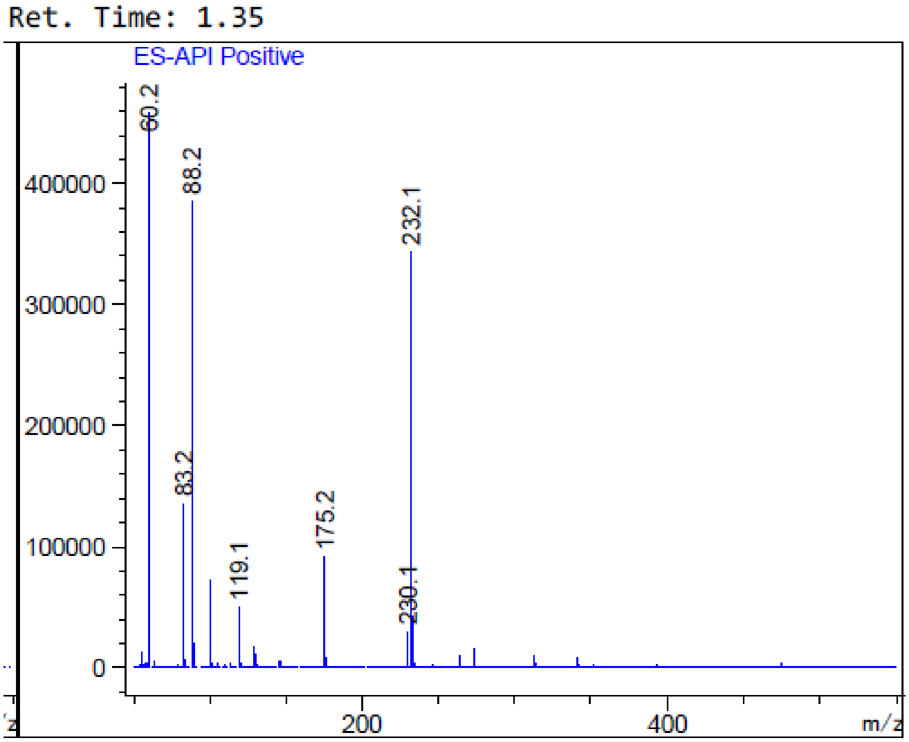


**Acetophenone (7)**

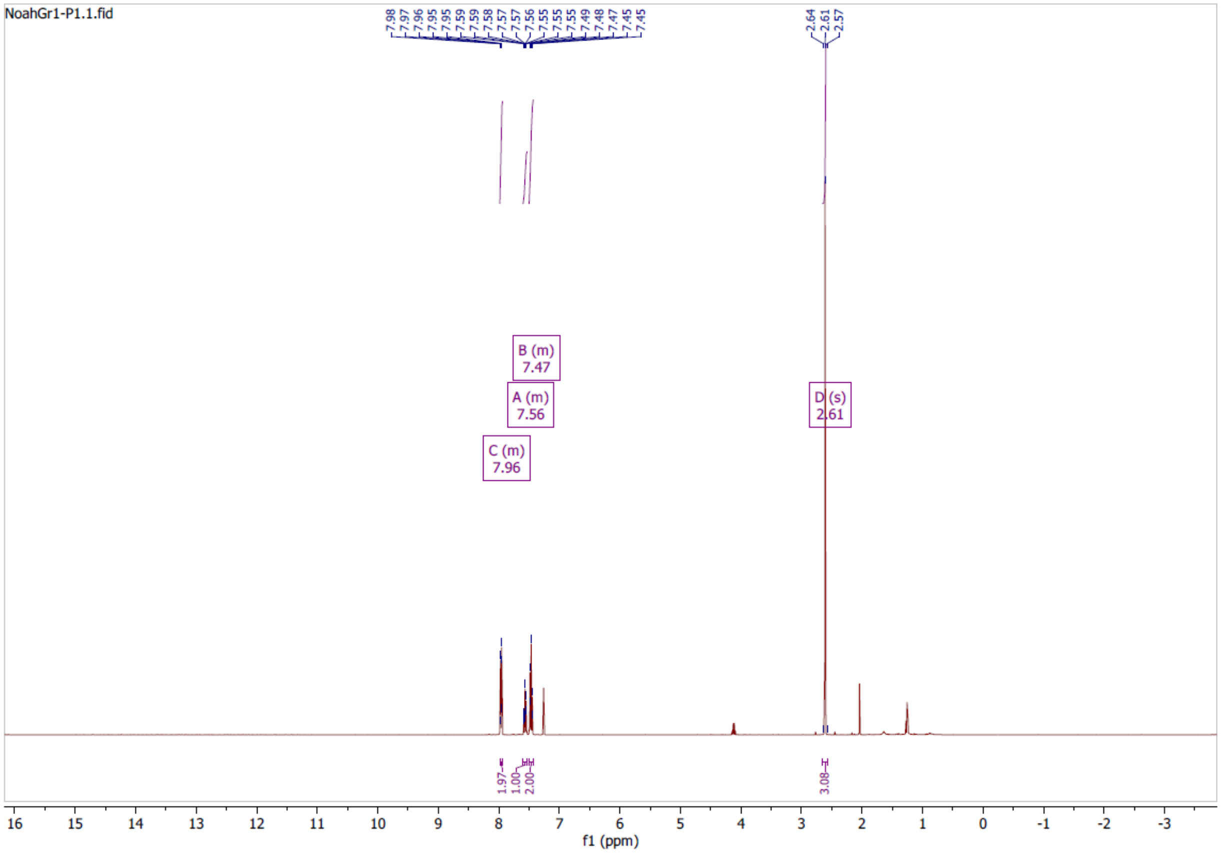

**(S)-2-acetamido-N-methyl-3-(2-(perfluoropropan-2-yl)-1H-indol-3-yl)propenamide (11)**

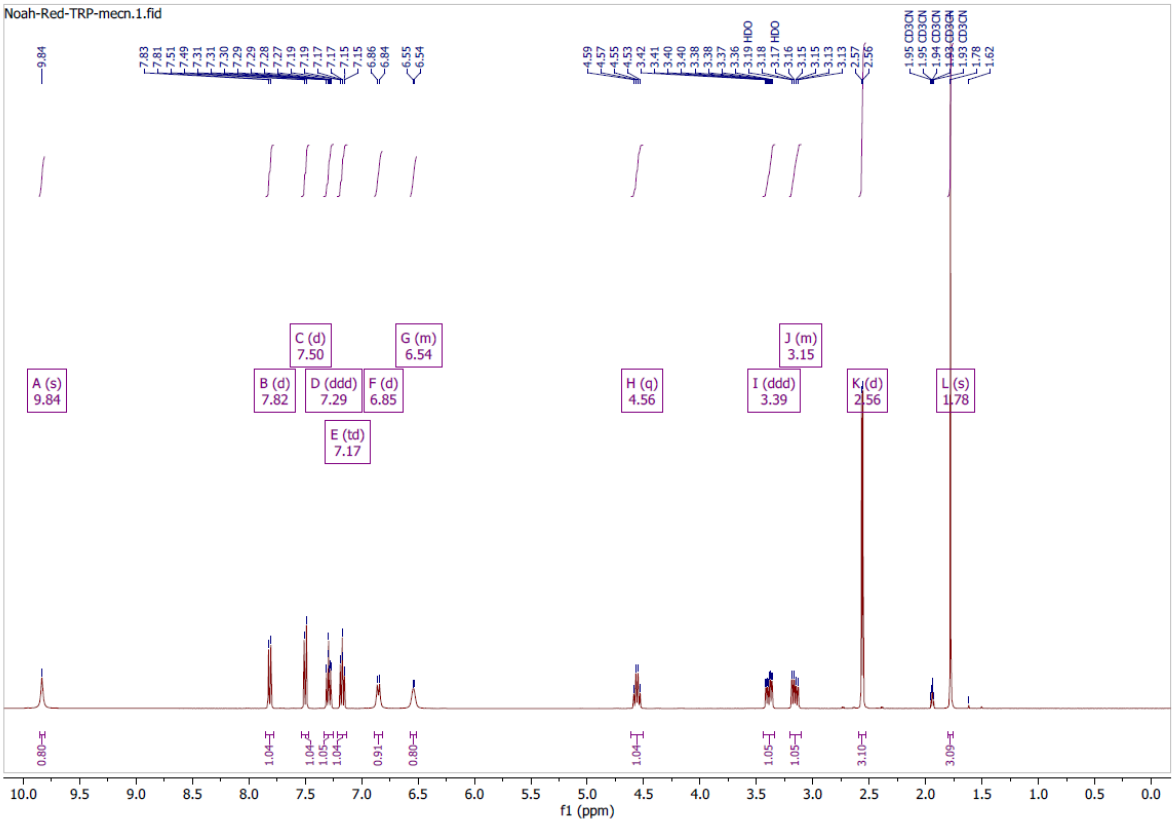

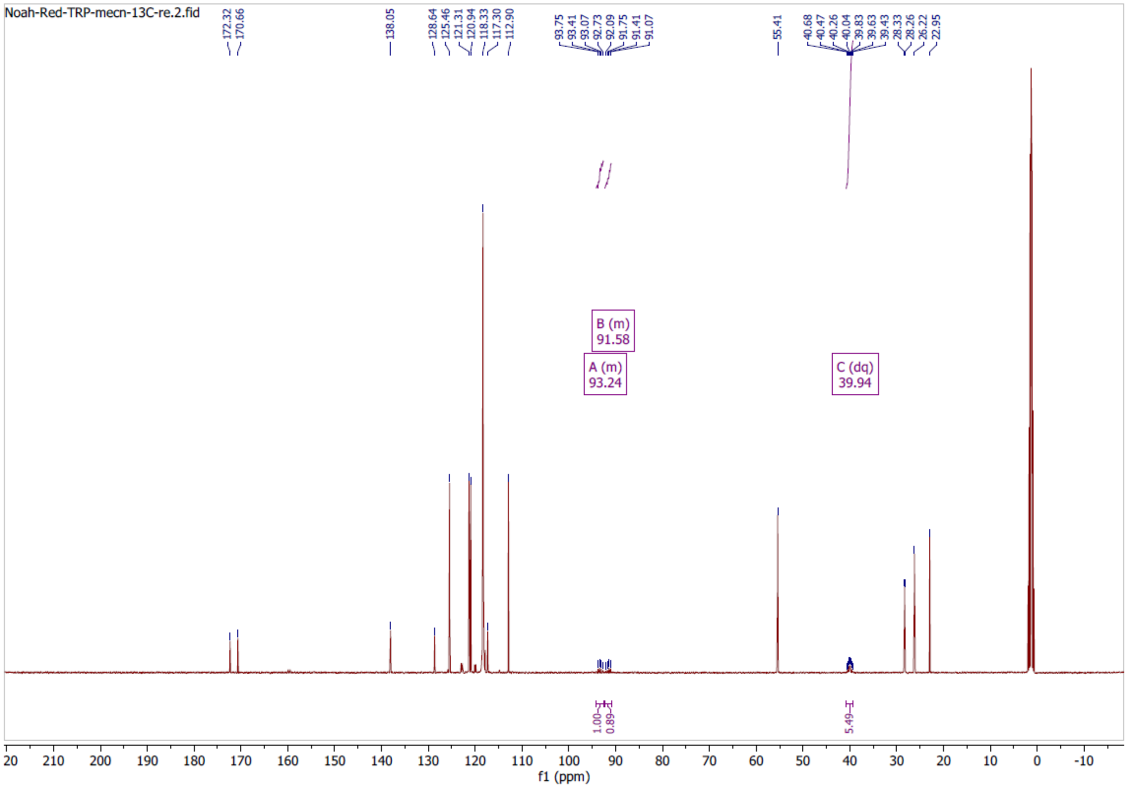

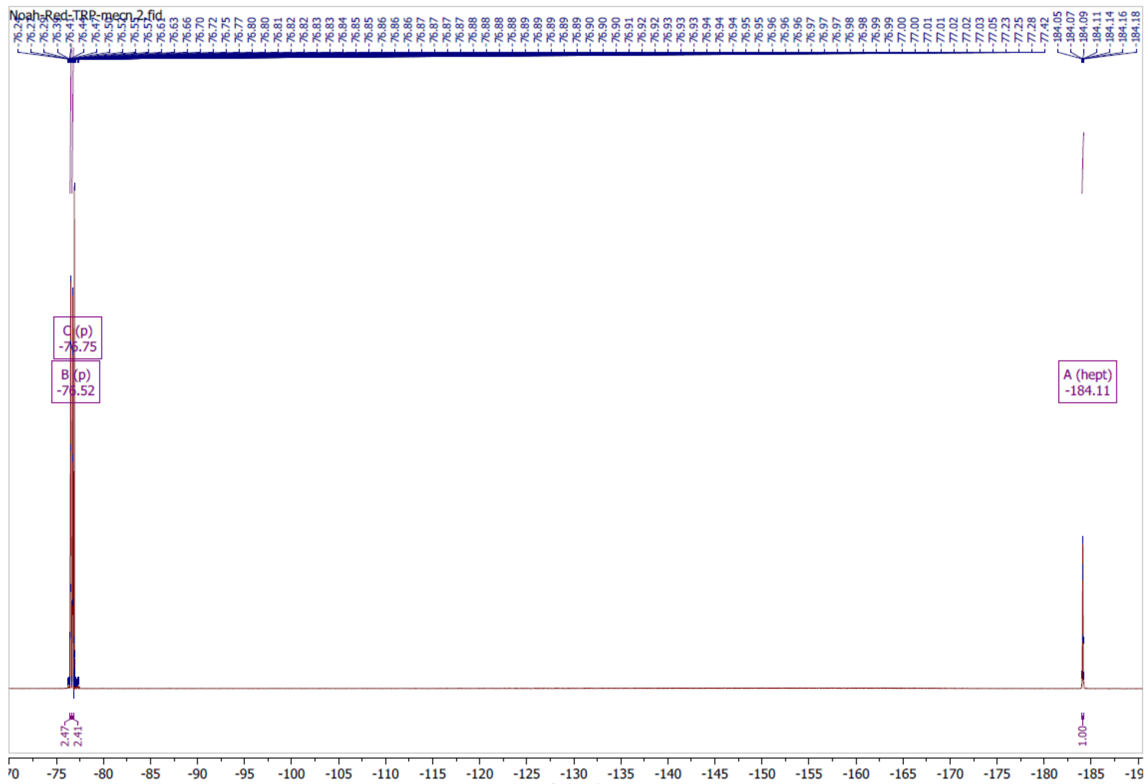
Noah-Red-TRP-mecn 2.fid
C (p)
-76.75
B (p)
-76.52
A (hept)
-184.11
2.47
2.41
1.00

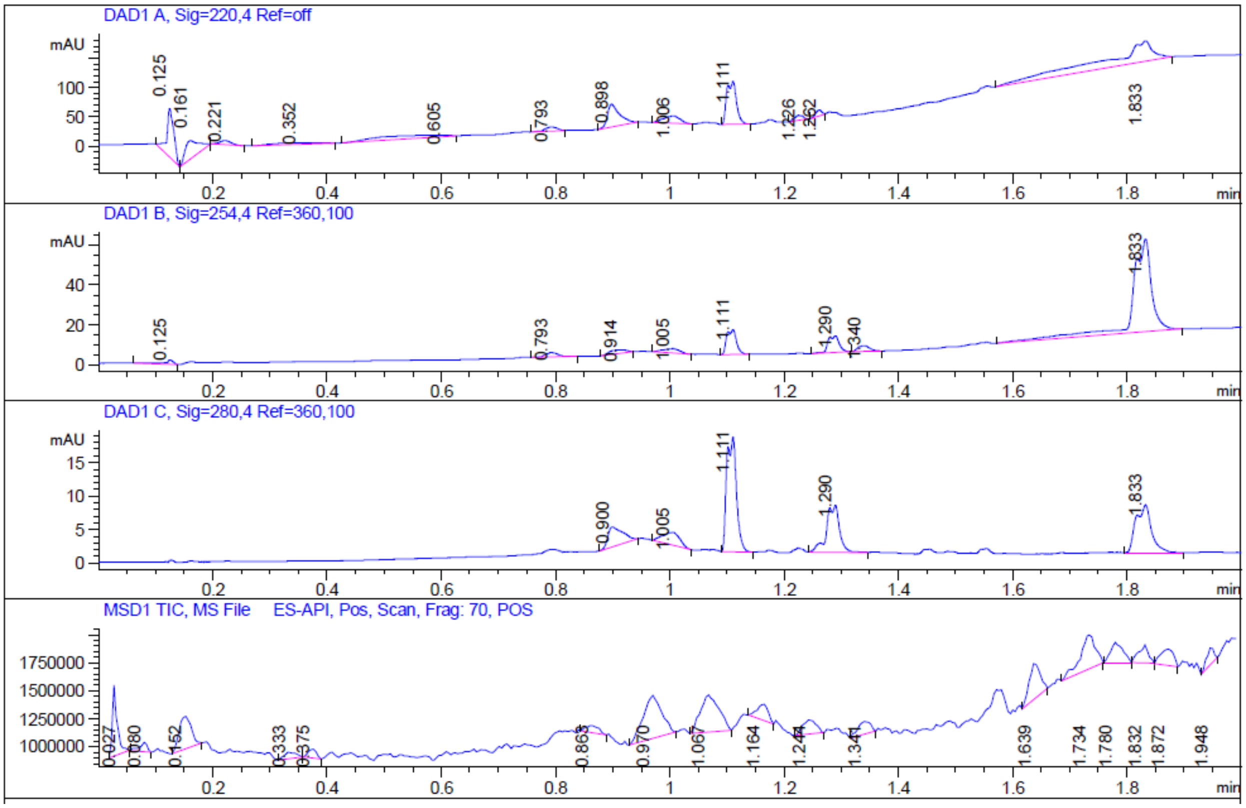
DAD1 A, Sig=220,4 Ref=off
mAU
DAD1 B, Sig=254,4 Ref=360,100
DAD1 C, Sig=280,4 Ref=360,100
MSD1 TIC, MS File ES-API, Pos, Scan, Frag: 70, POS
min

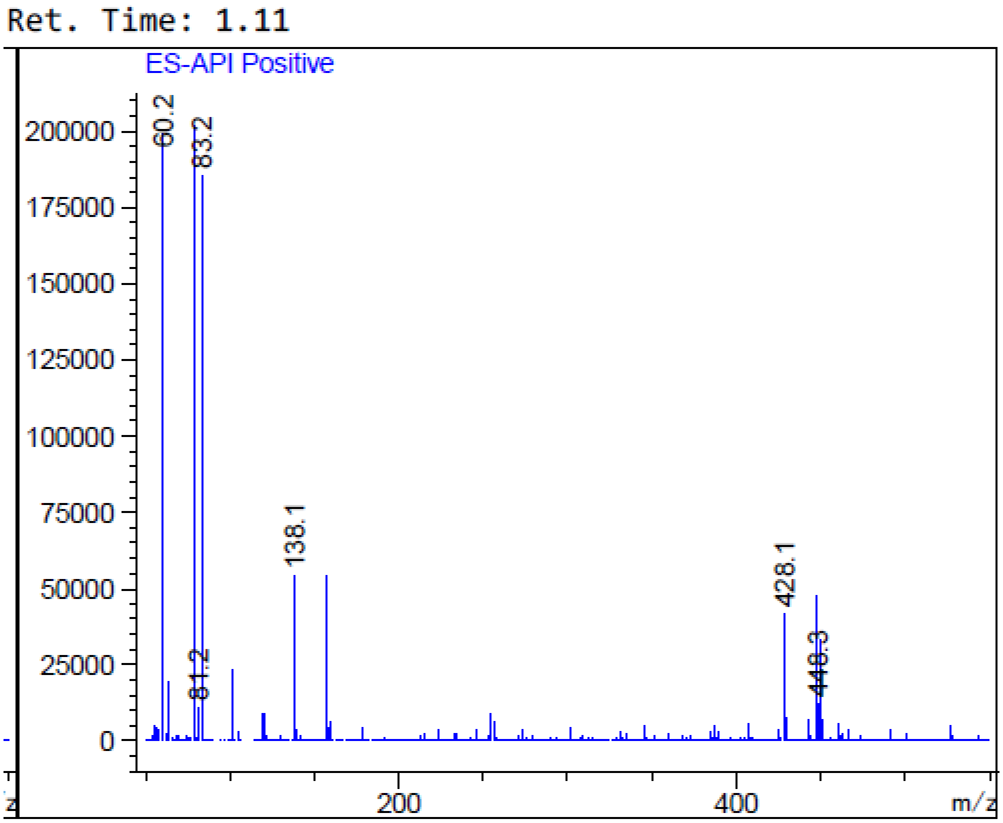


**3-(5-morpholinopyridin-2-yl)oxetane-3-carbonitrile (12)**

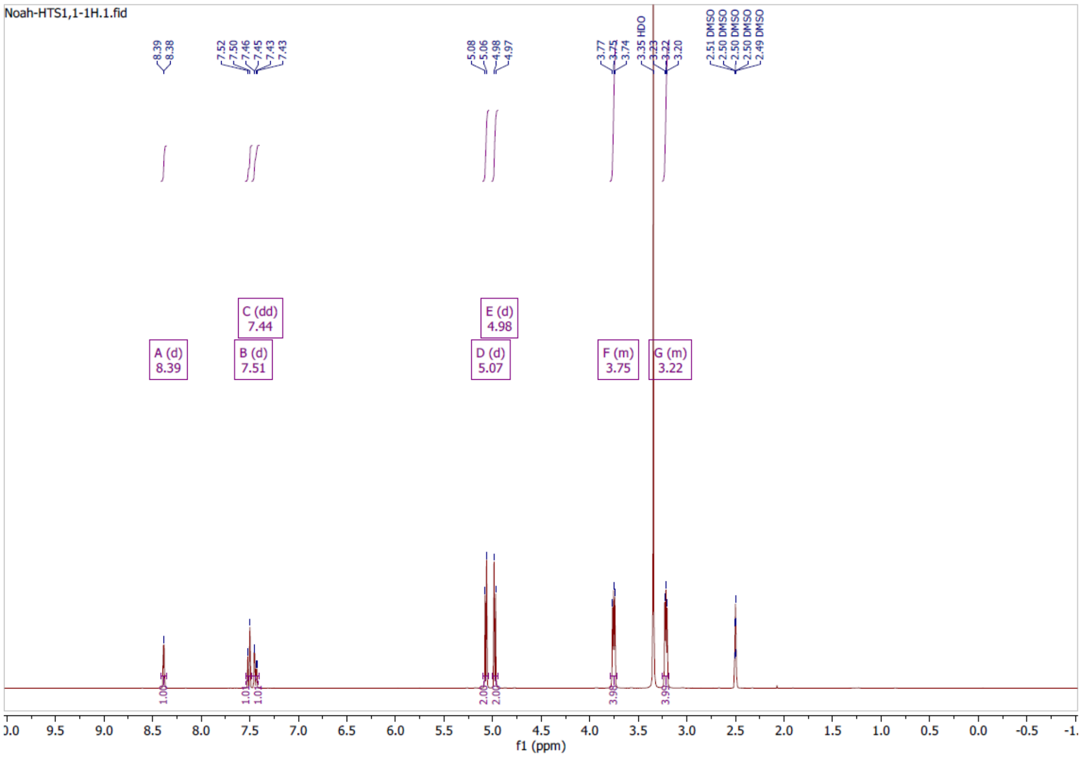

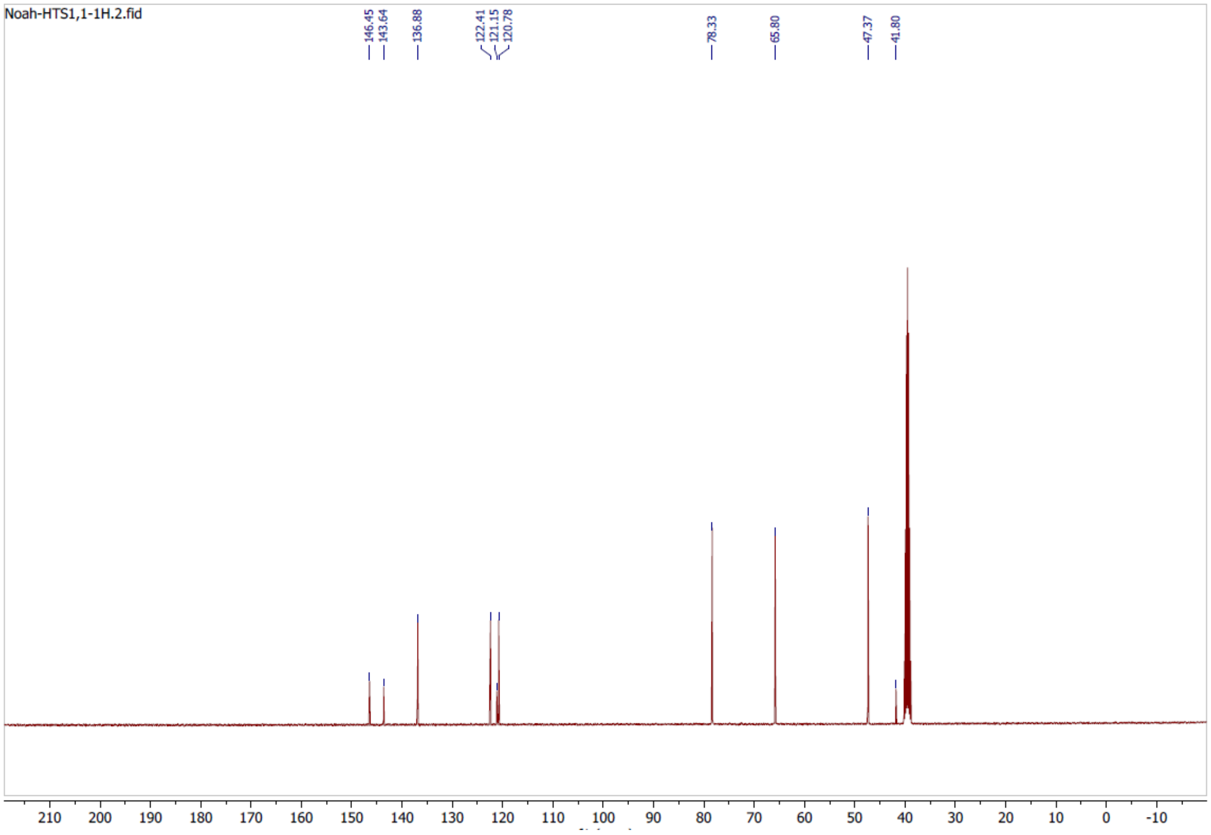
Noah-HTS1,1-1H.2.fid
146.45
143.64
136.88
122.41
121.15
120.78
78.33
65.80
47.37
41.80
210 200 190 180 170 160 150 140 130 120 110 100 90 80 70 60 50 40 30 20 10 0 -10

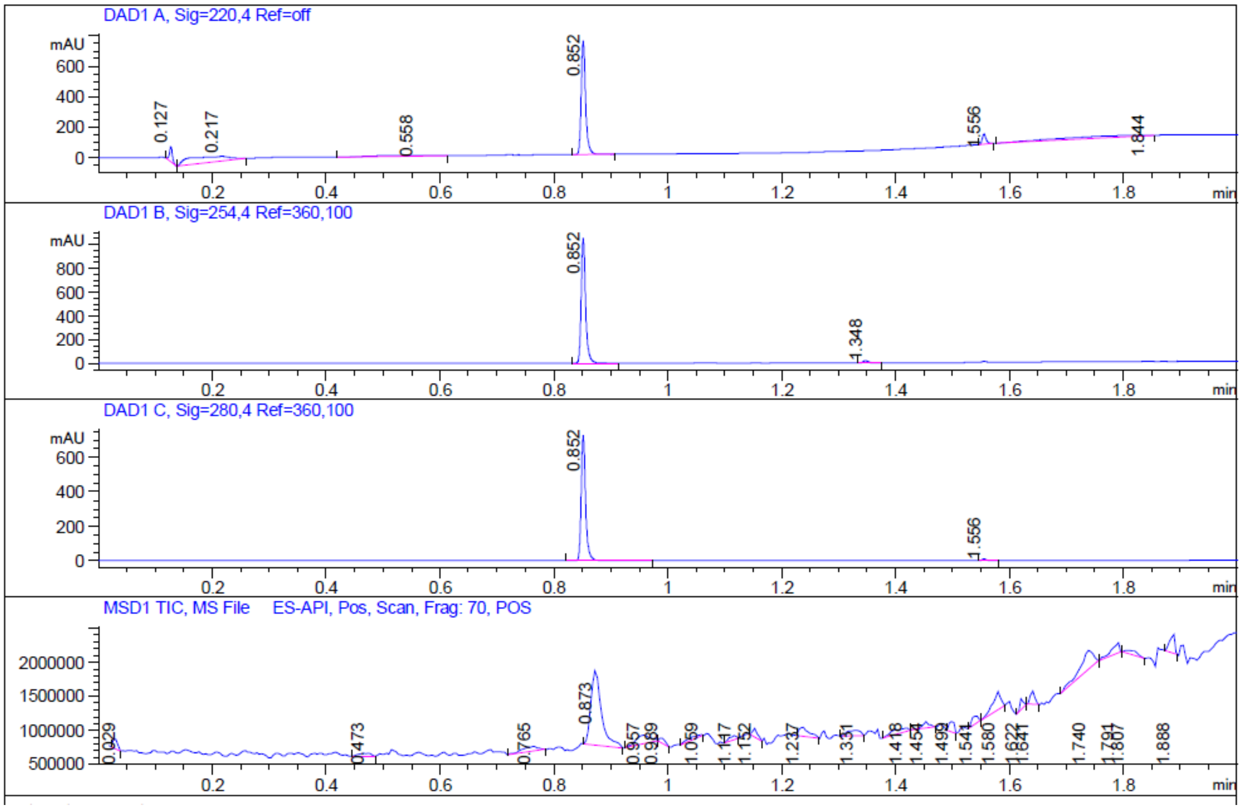
DAD1 A, Sig=220,4 Ref=off
mAU
0.127
0.217
0.558
0.852
1.556
1.844
DAD1 B, Sig=254,4 Ref=360,100
mAU
0.852
1.348
DAD1 C, Sig=280,4 Ref=360,100
mAU
0.852
1.556
MSD1 TIC, MS File ES-API, Pos, Scan, Frag: 70, POS
0.473
0.765
0.873
1.740
1.888
min

Ret. Time: 0.85

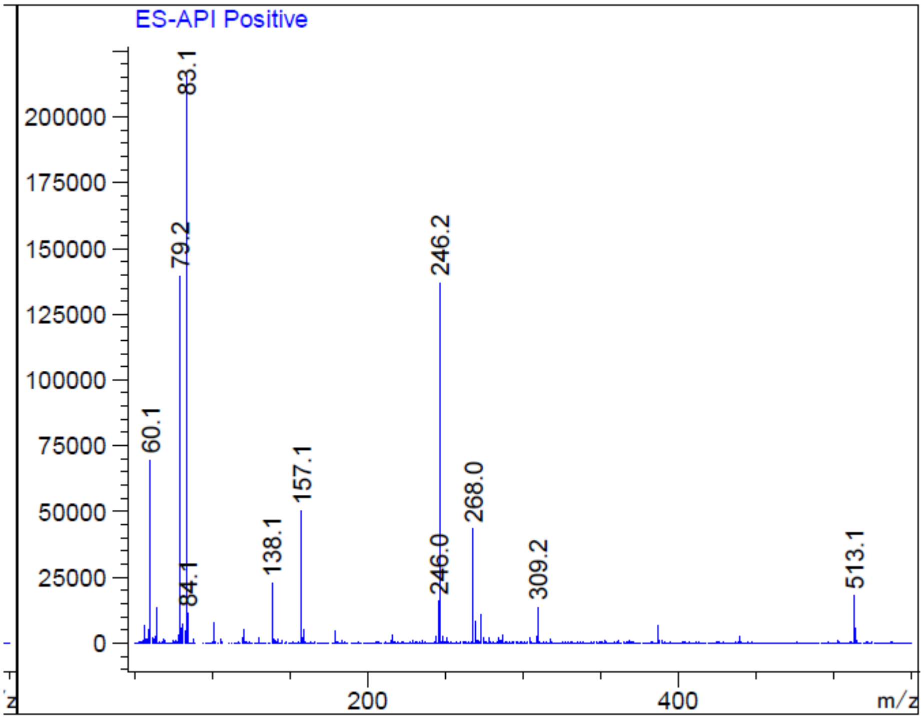


**4-(1-(phenylsulfonyl)-1H-pyrrolo[2,3-b]pyridin-5-yl)morpholine (13)**

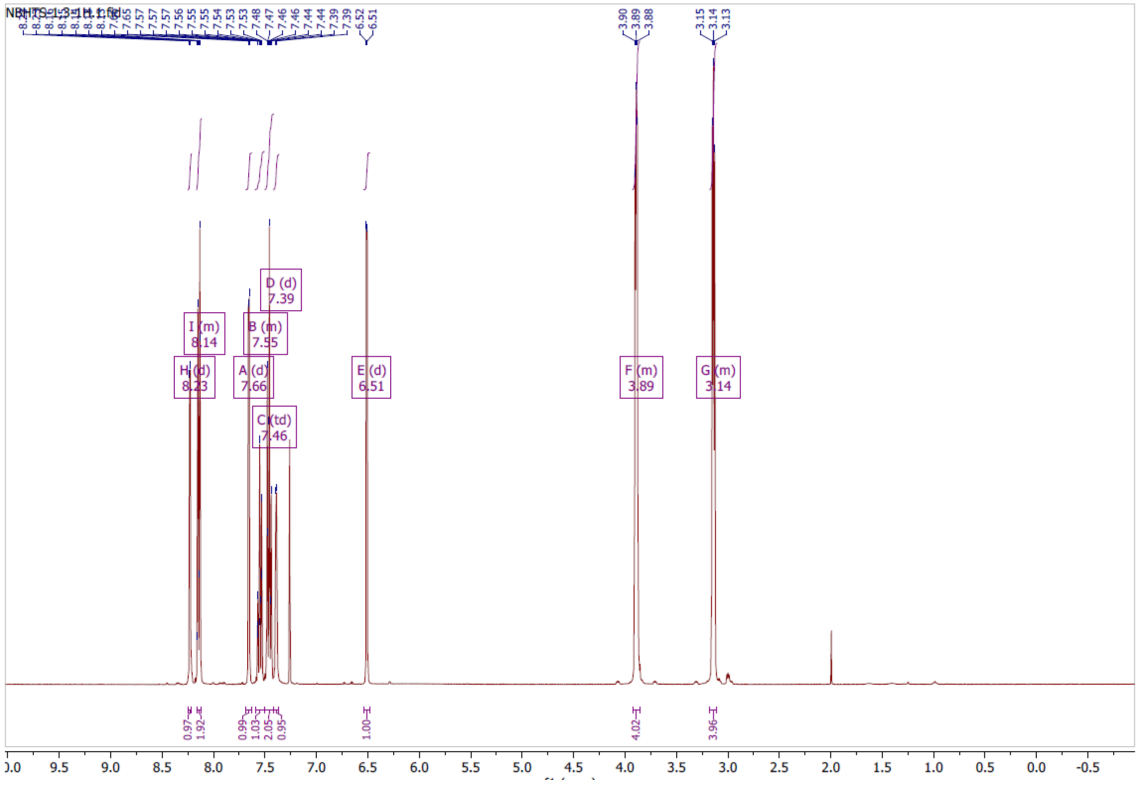

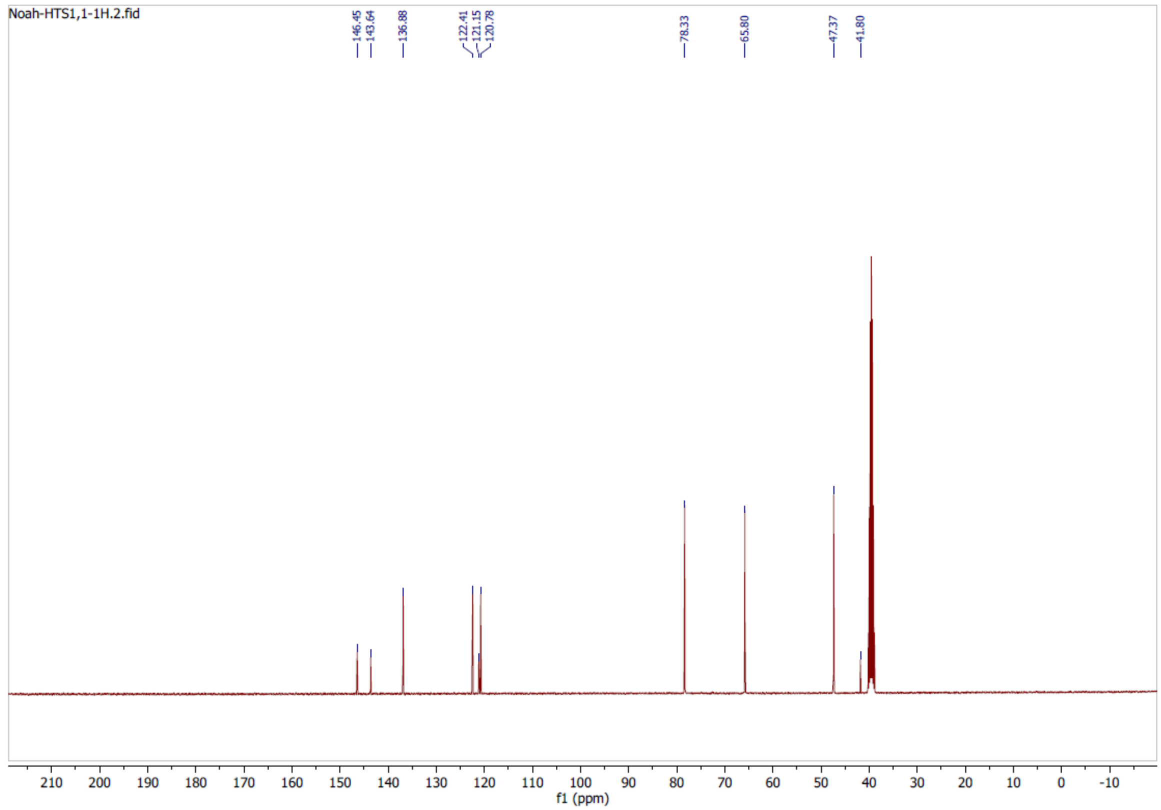
Noah-HTS1,1-1H.2.fid
146.45
143.64
136.88
122.41
121.15
120.78
78.33
65.80
47.37
41.80
f1 (ppm)

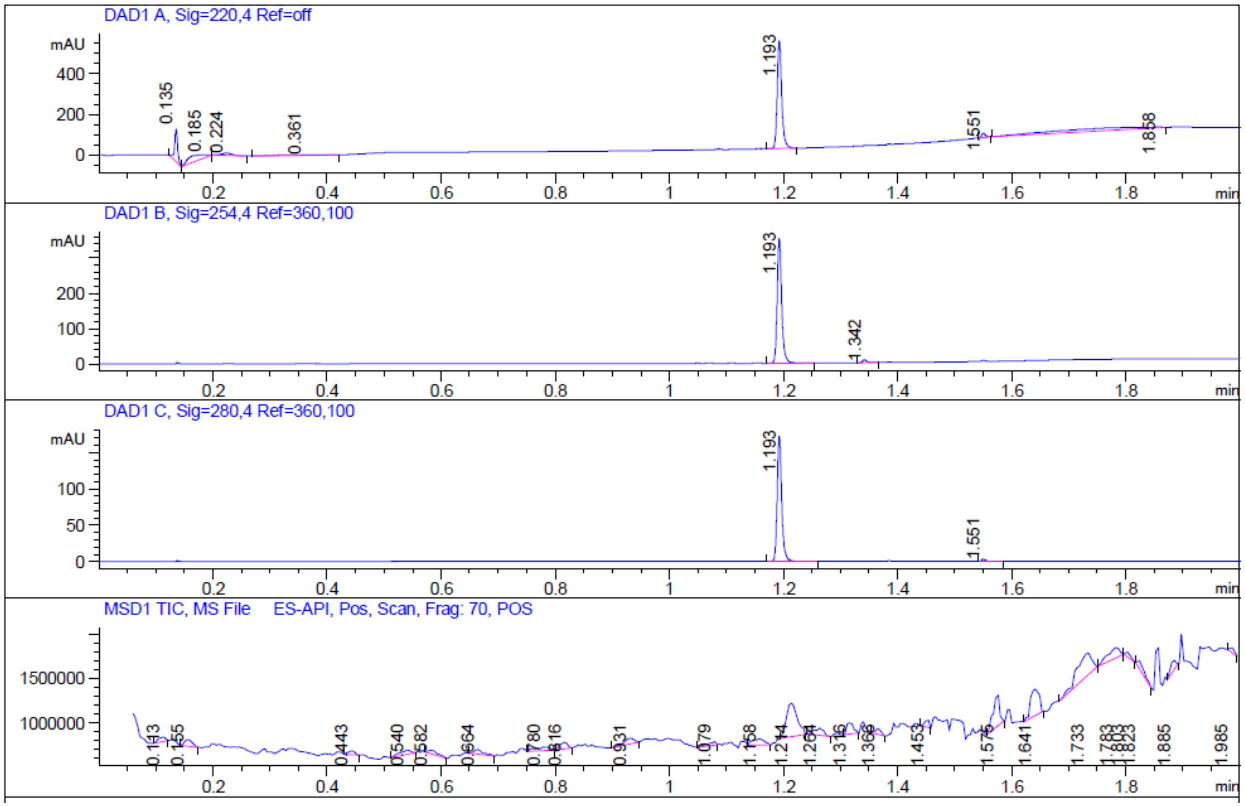
DAD1 A, Sig=220,4 Ref=off
mAU
DAD1 B, Sig=254,4 Ref=360,100
DAD1 C, Sig=280,4 Ref=360,100
MSD1 TIC, MS File ES-API, Pos, Scan, Frag: 70, POS
min

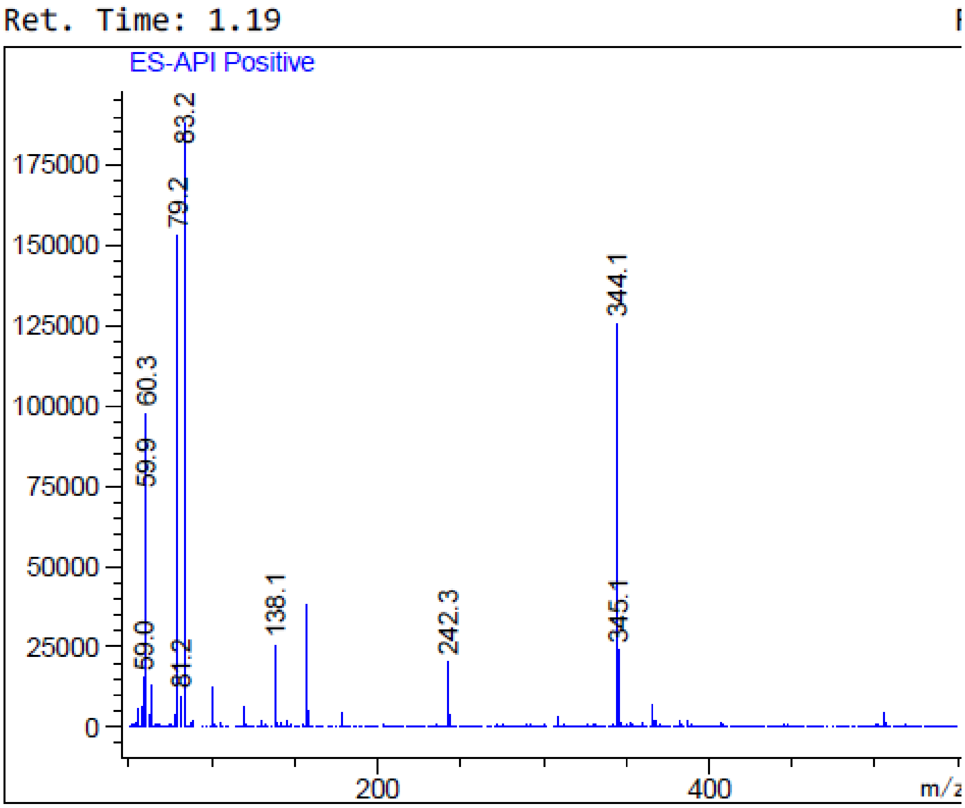


**4-methyl-3-morpholinobenzonitrile (14)**

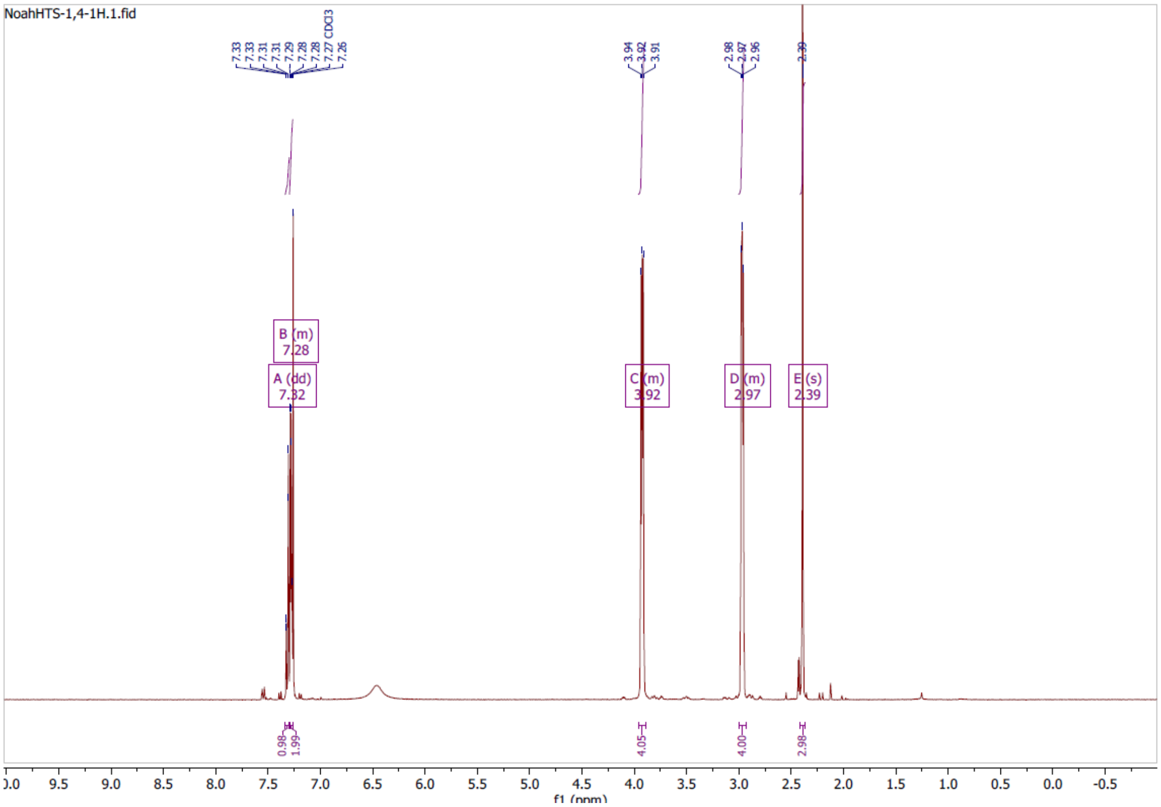

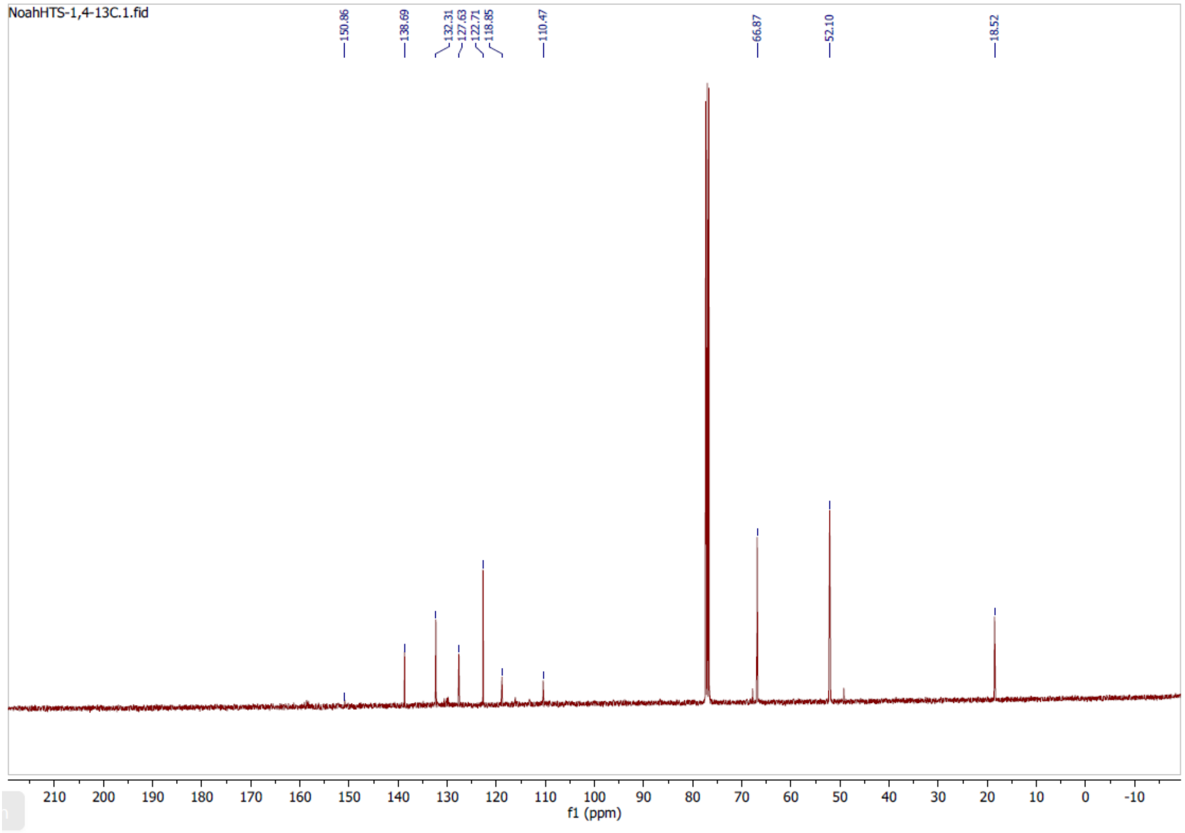
NoahHTS-1,4-13C.1.fid
150.86
138.69
132.31
127.63
122.71
118.85
110.47
66.87
52.10
18.52
f1 (ppm)

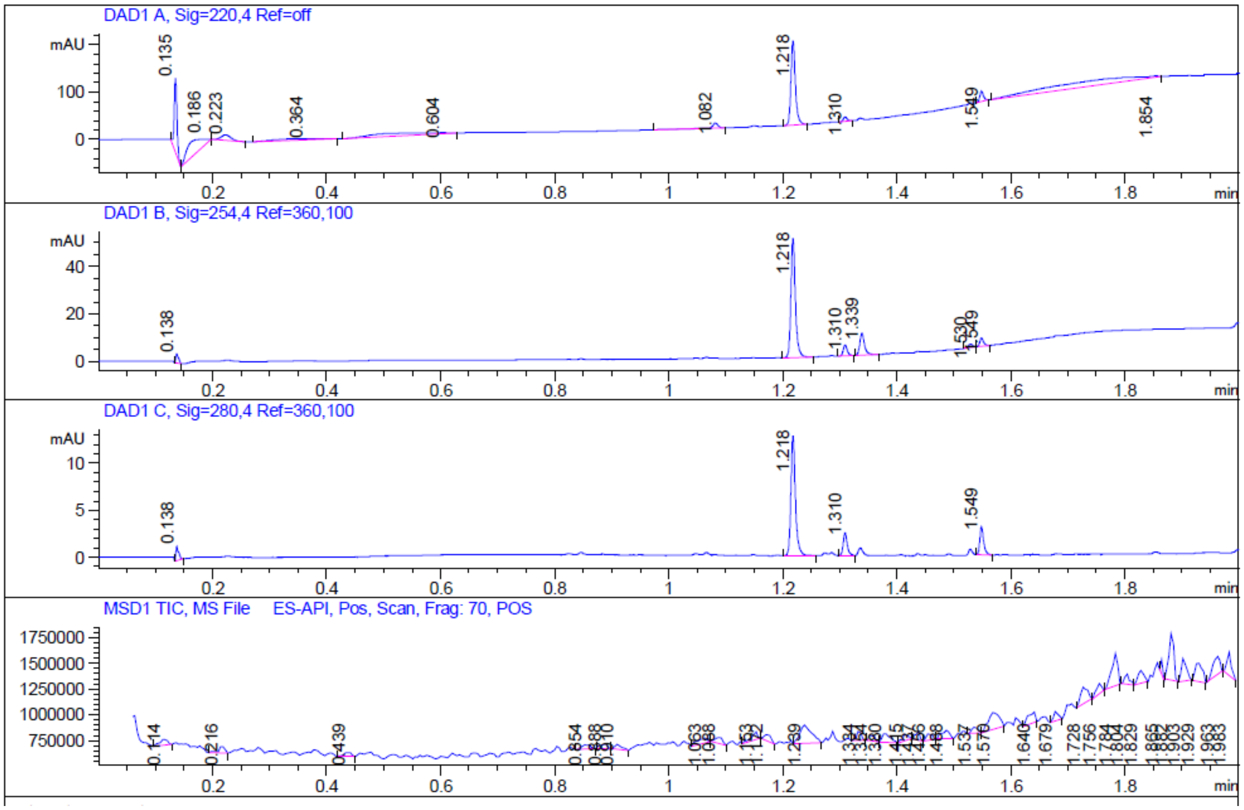
DAD1 A, Sig=220,4 Ref=off
DAD1 B, Sig=254,4 Ref=360,100
DAD1 C, Sig=280,4 Ref=360,100
MSD1 TIC, MS File ES-API, Pos, Scan, Frag: 70, POS
mAU
min

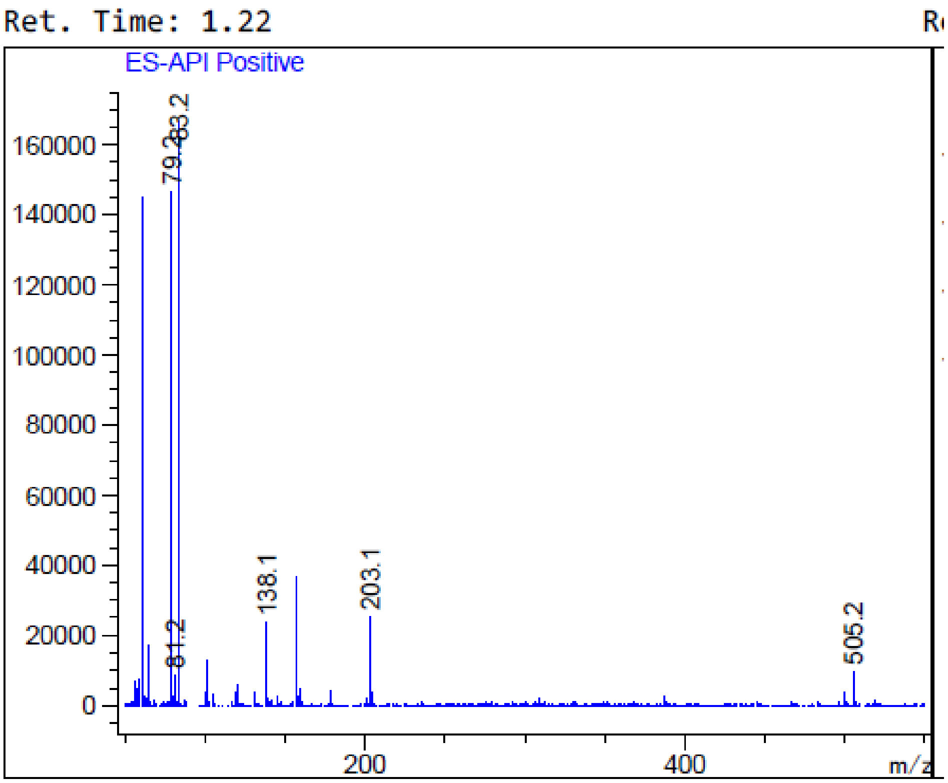


**3-(5-((6-hydroxyspiro[3.3]heptan-2-yl)amino)pyridin-2-yl)oxetane-3-carbonitrile (15)**

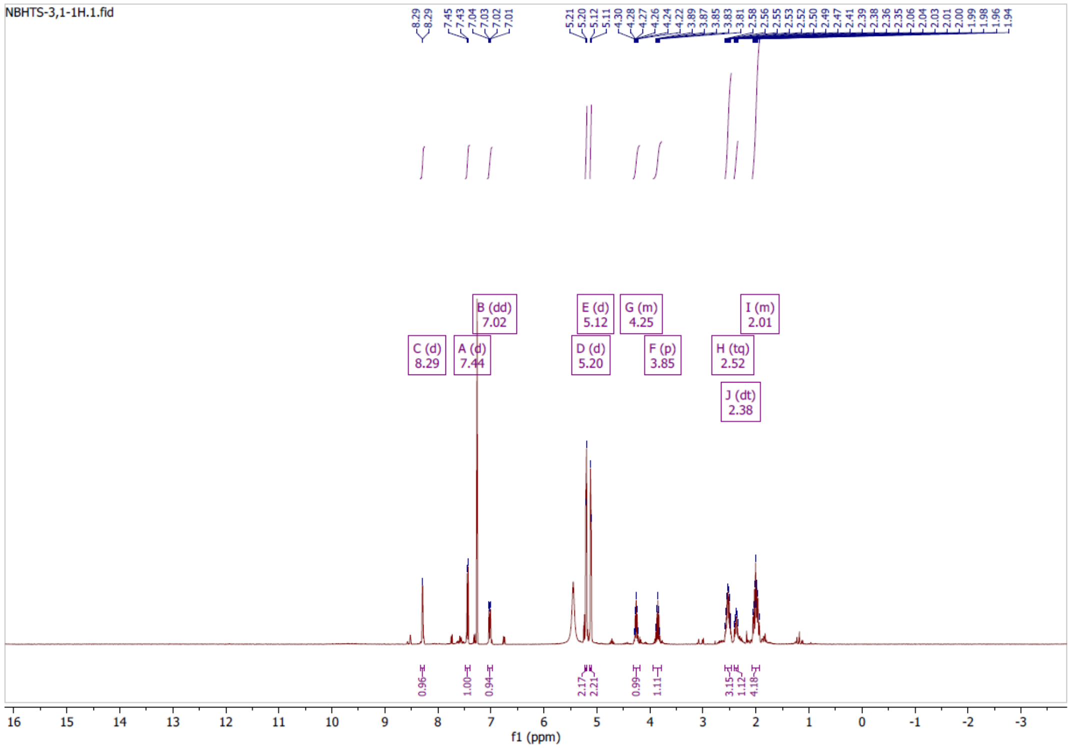

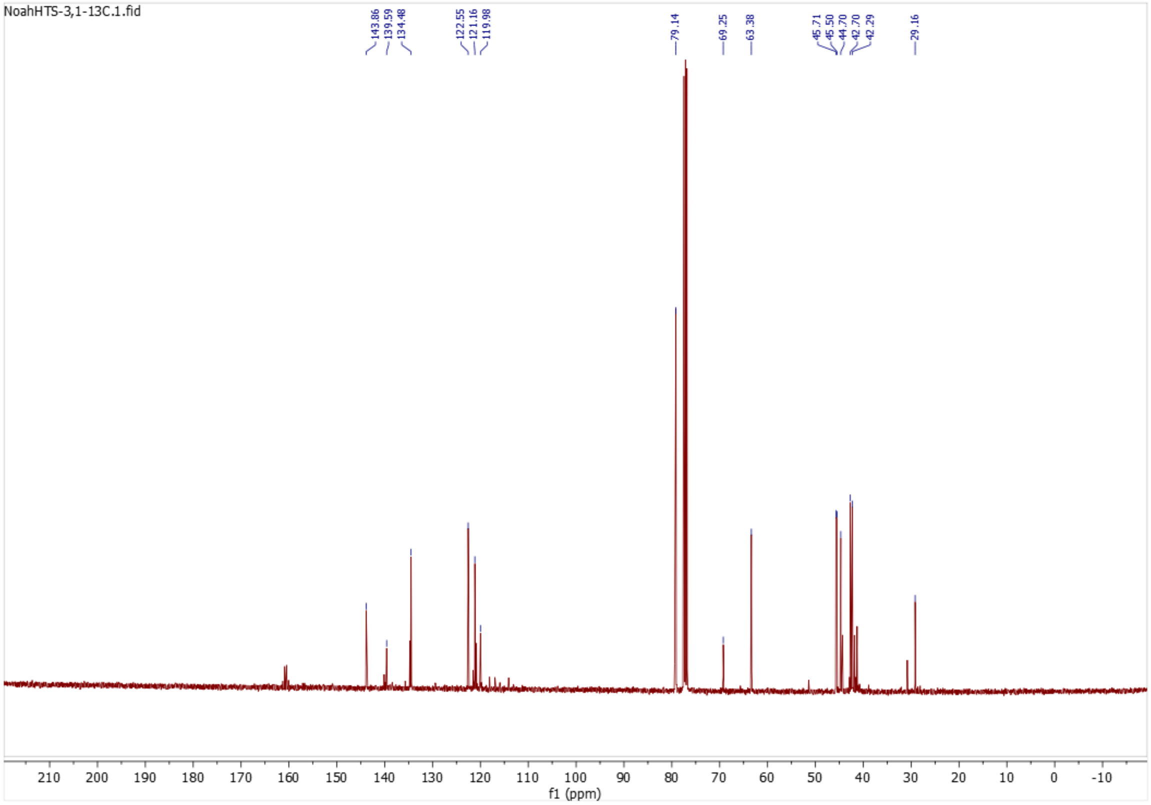
NoahHTS-3,1-13C.1.fid
143.86
139.59
134.48
122.55
121.16
119.98
79.14
69.25
63.38
45.71
45.50
44.70
42.70
42.29
29.16
f1 (ppm)

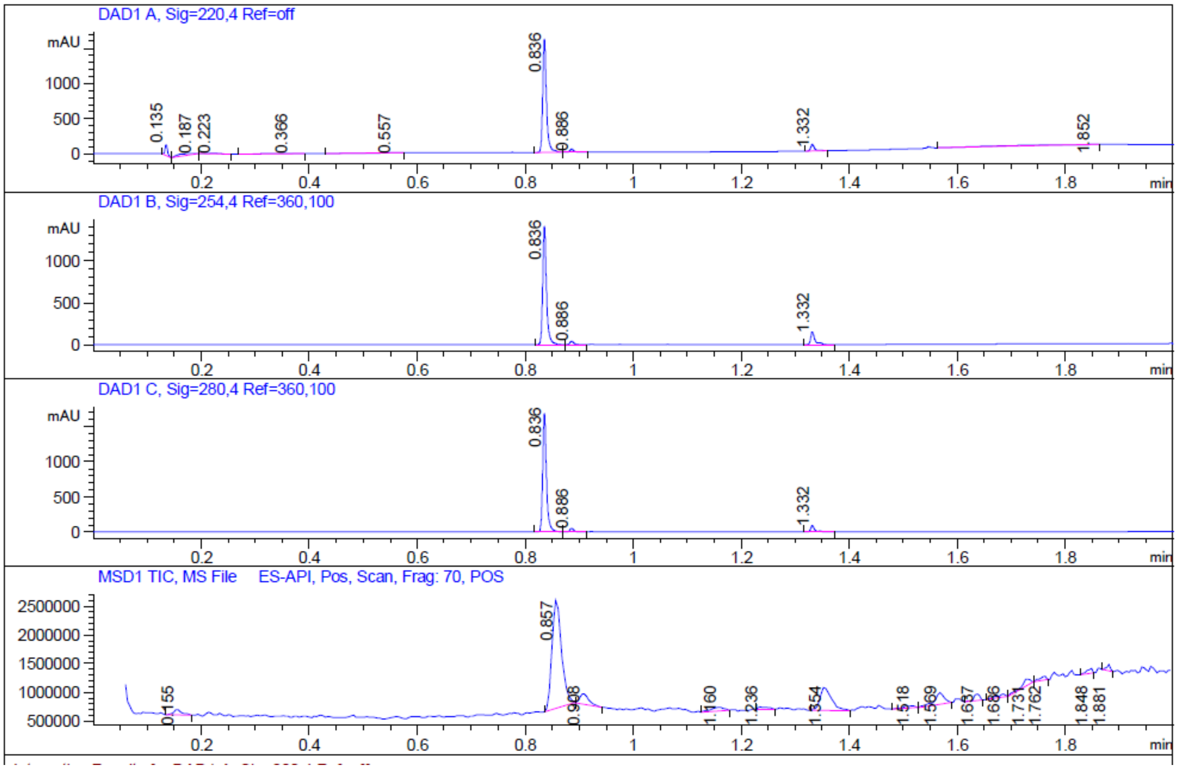
DAD1 A, Sig=220,4 Ref=off
DAD1 B, Sig=254,4 Ref=360,100
DAD1 C, Sig=280,4 Ref=360,100
MSD1 TIC, MS File ES-API, Pos, Scan, Frag: 70, POS
mAU
0.836
0.886
1.332
0.135
0.187
0.223
0.366
0.557
1.852
0.857
min

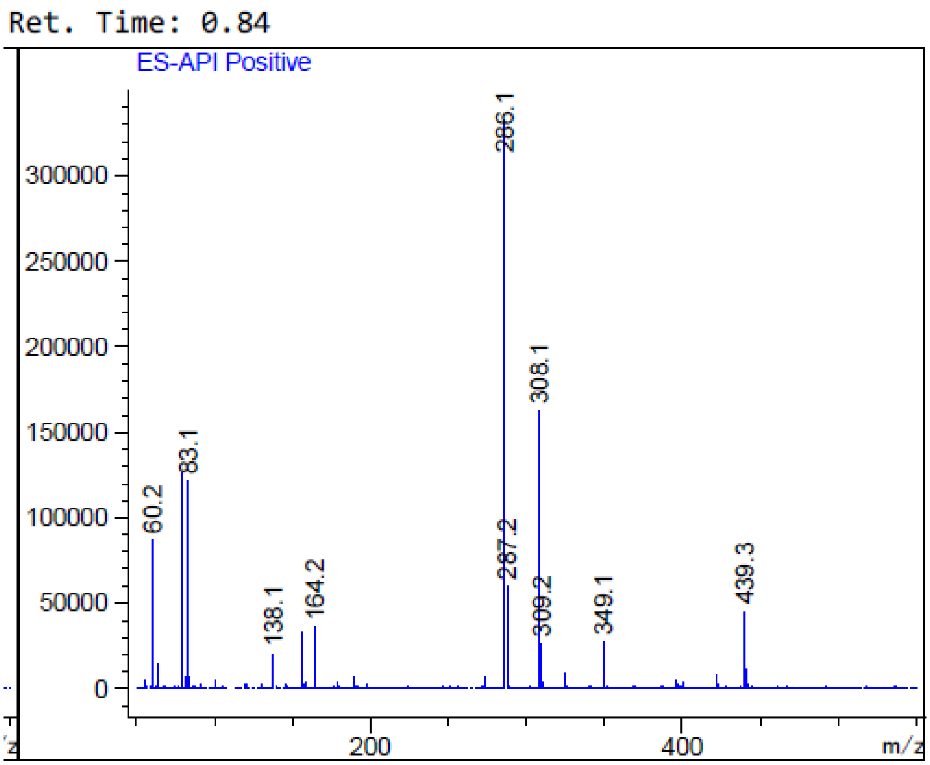


**6-((1-(phenylsulfonyl)-1H-pyrrolo[2,3-b]pyridin-5-yl)amino)spiro[3.3]heptan-2-ol (16)**

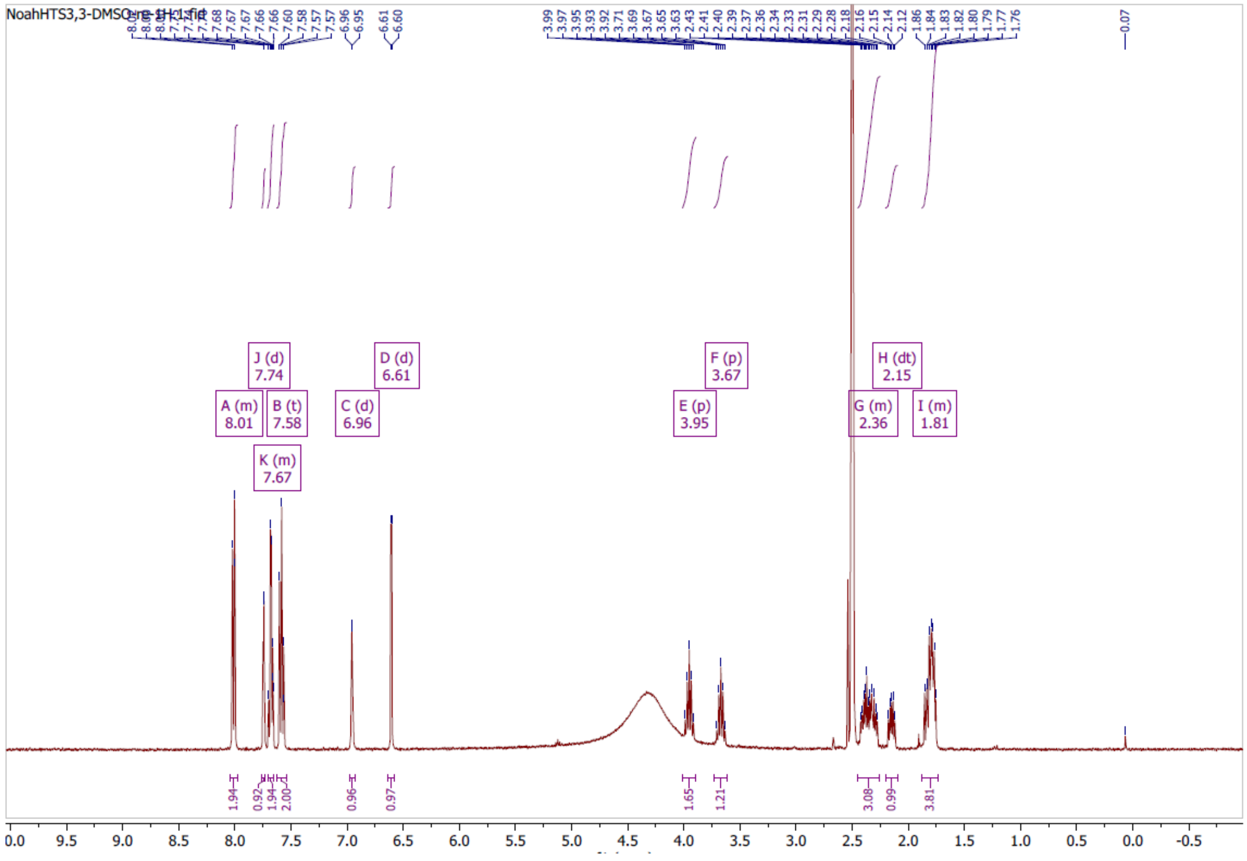

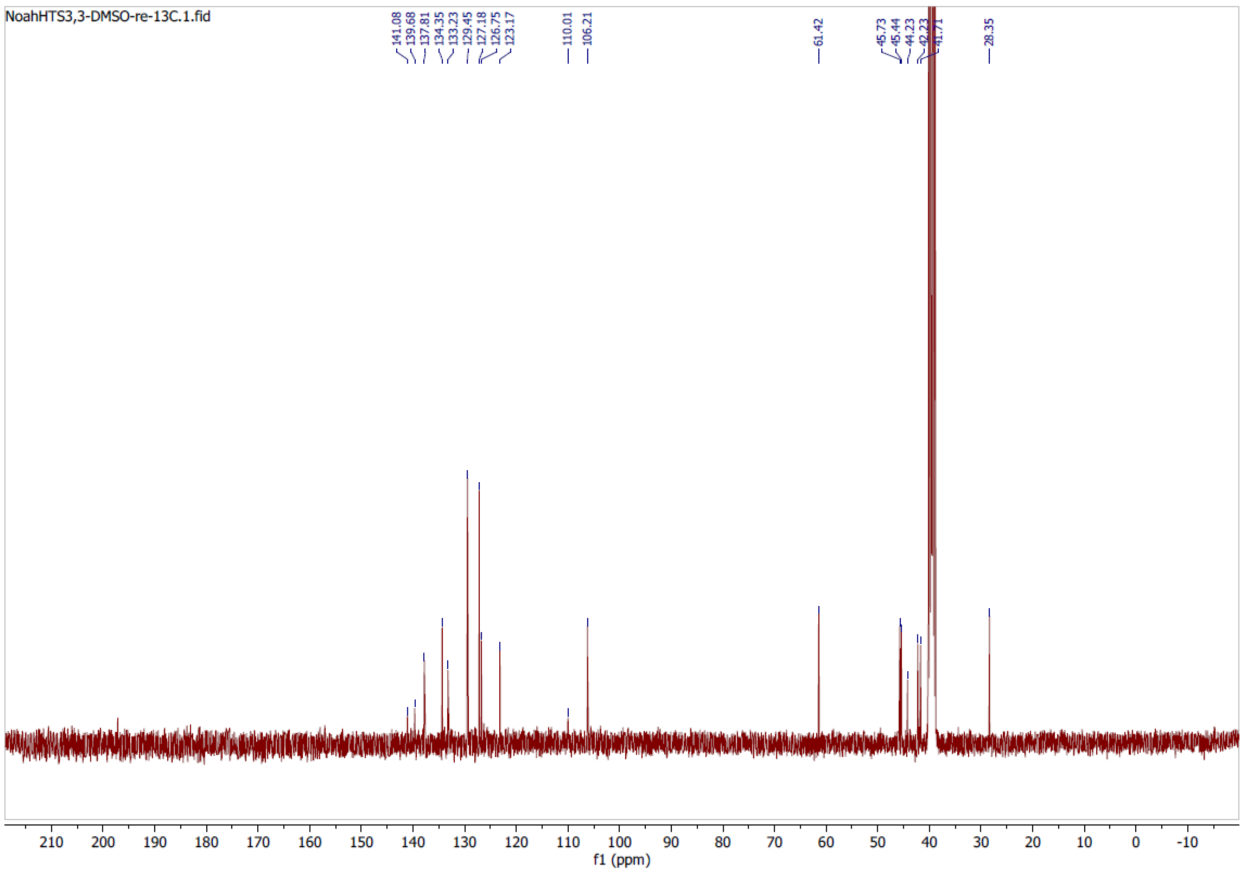
NoahHTS3,3-DMSO-re-13C.1.fid
141.08
139.68
137.81
134.35
133.23
129.45
127.18
126.75
123.17
110.01
106.21
61.42
45.73
45.44
44.23
42.23
41.71
28.35
f1 (ppm)

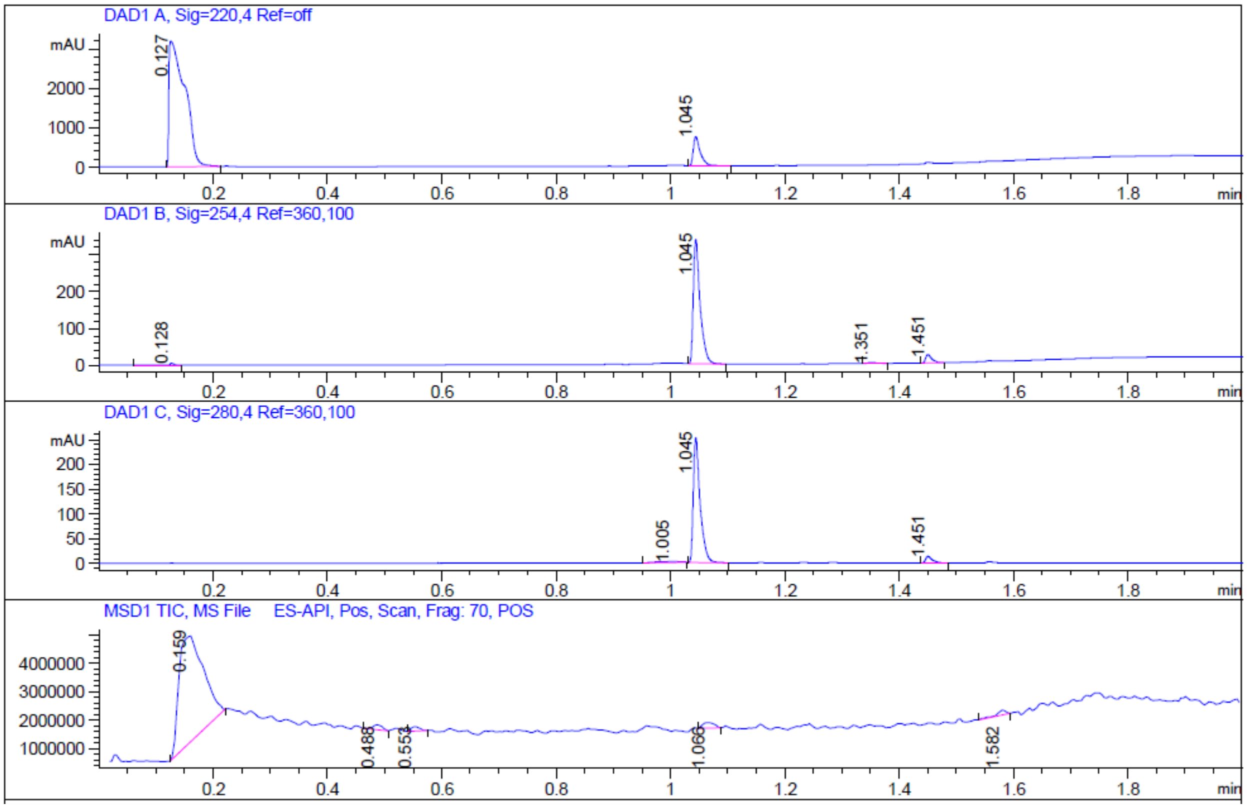
DAD1 A, Sig=220,4 Ref=off
DAD1 B, Sig=254,4 Ref=360,100
DAD1 C, Sig=280,4 Ref=360,100
MSD1 TIC, MS File ES-API, Pos, Scan, Frag: 70, POS

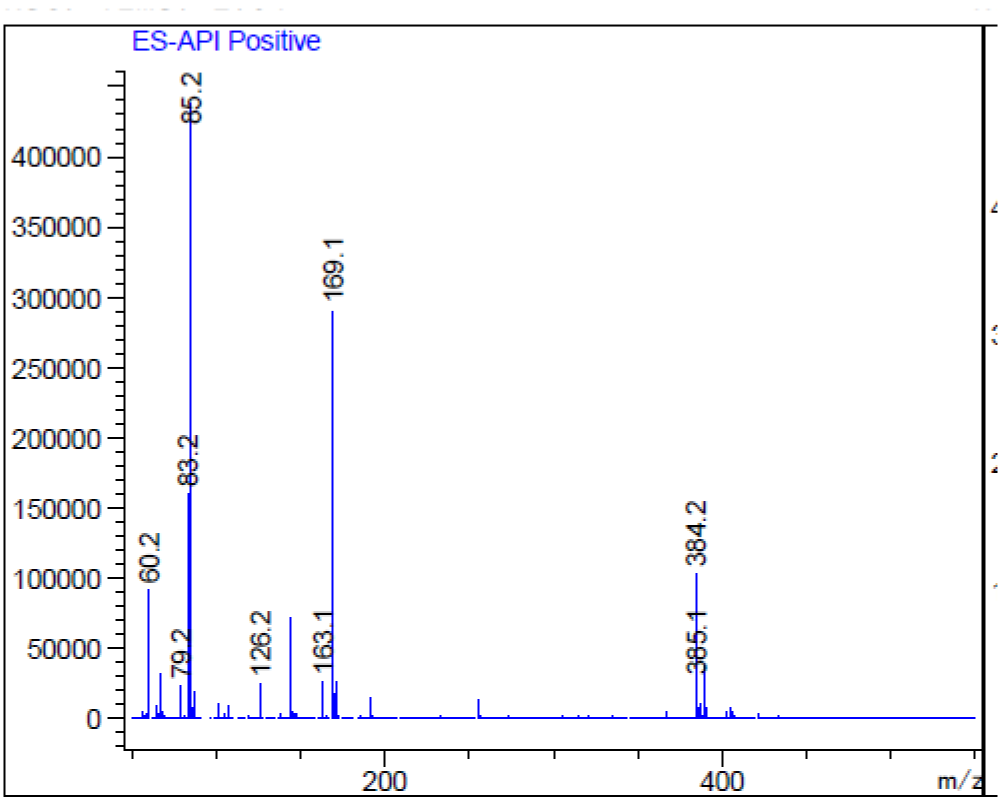


**3-((6-hydroxyspiro[3.3]heptan-2-yl)amino)-4-methylbenzonitrile (17)**

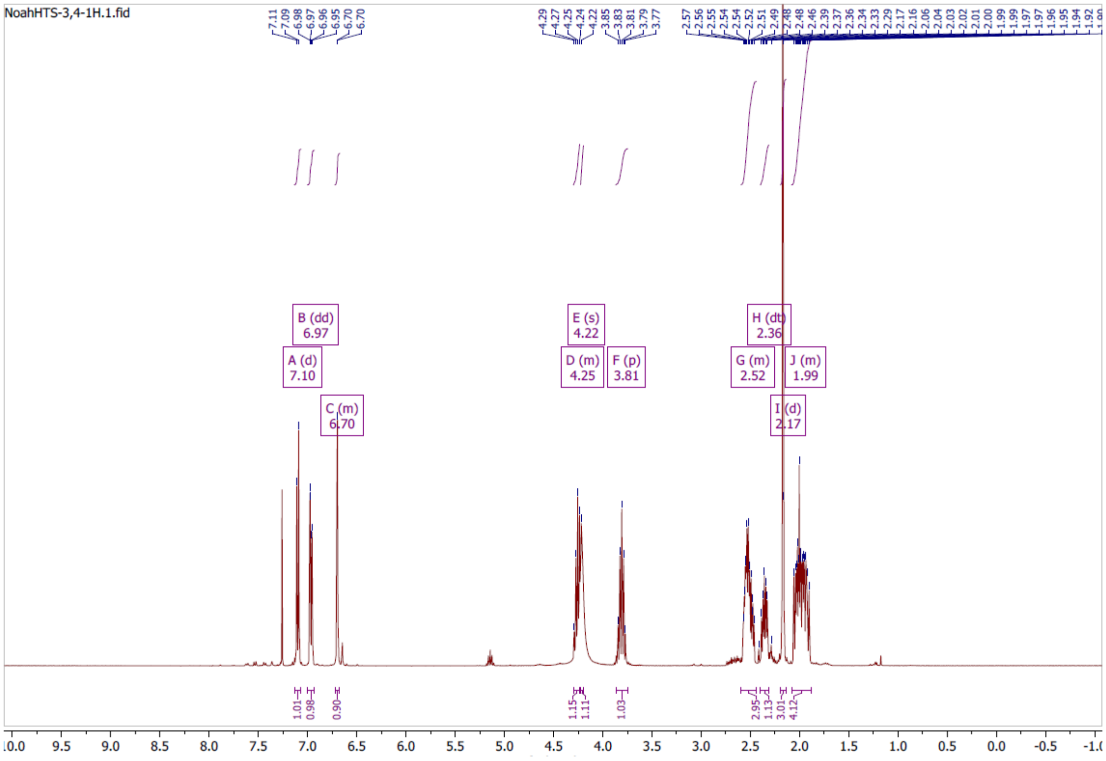

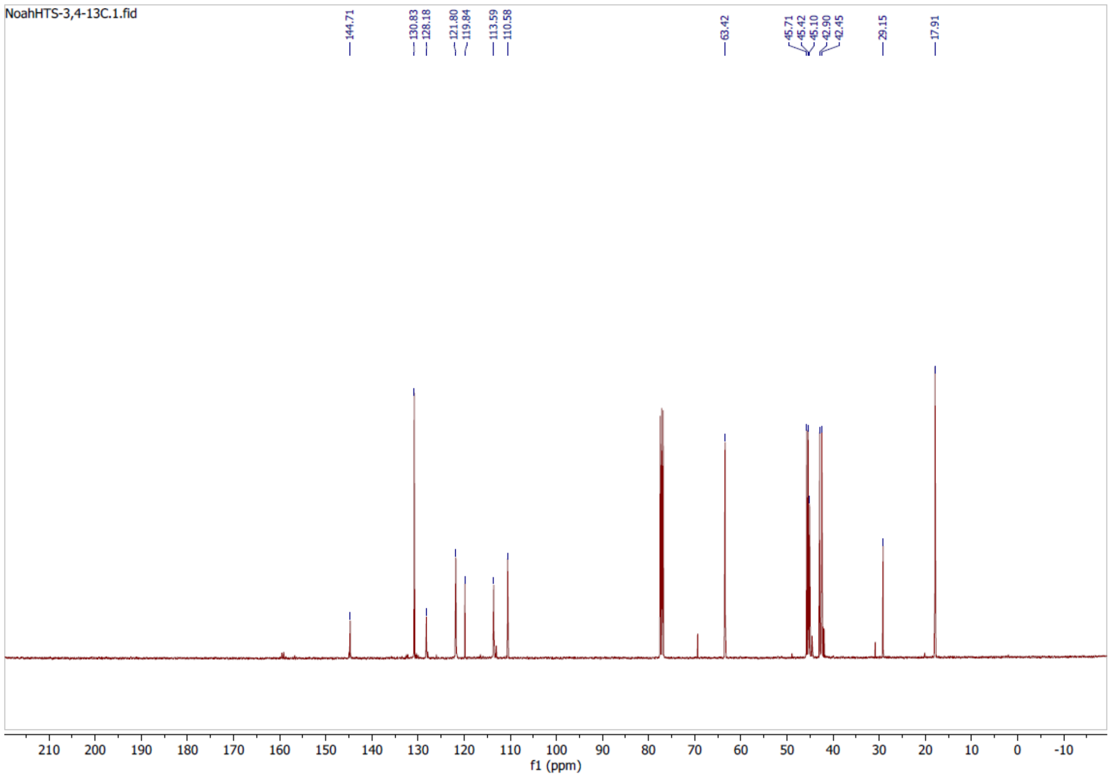
NoahHTS-3,4-13C.1.fid
144.71
130.83
128.18
121.80
119.84
113.59
110.58
63.42
45.71
45.42
45.10
42.90
42.45
29.15
17.91
210 200 190 180 170 160 150 140 130 120 110 100 90 80 70 60 50 40 30 20 10 0 -10
f1 (ppm)

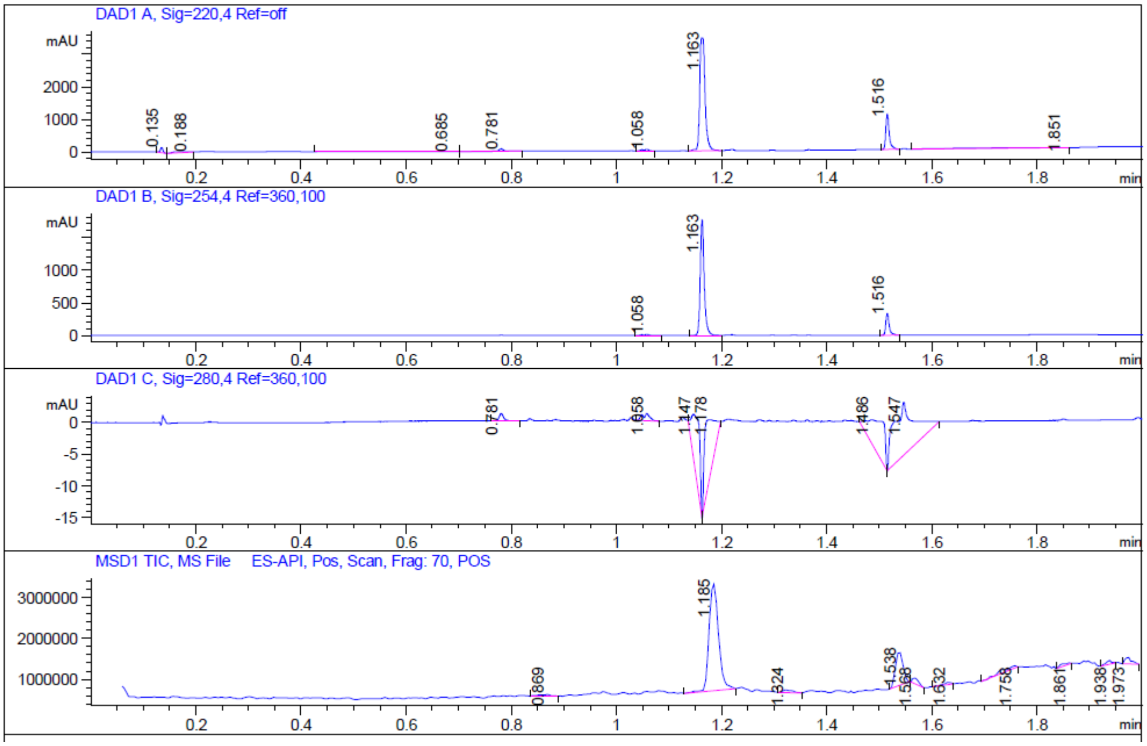
DAD1 A, Sig=220,4 Ref=off
mAU
1.163
1.516
0.135
0.188
0.685
0.781
1.058
1.851
DAD1 B, Sig=254,4 Ref=360,100
mAU
1.163
1.058
1.516
DAD1 C, Sig=280,4 Ref=360,100
mAU
MSD1 TIC, MS File ES-API, Pos, Scan, Frag: 70, POS
1.185
0.869
1.324
1.538
1.632
1.758
1.861
1.938
1.973
min

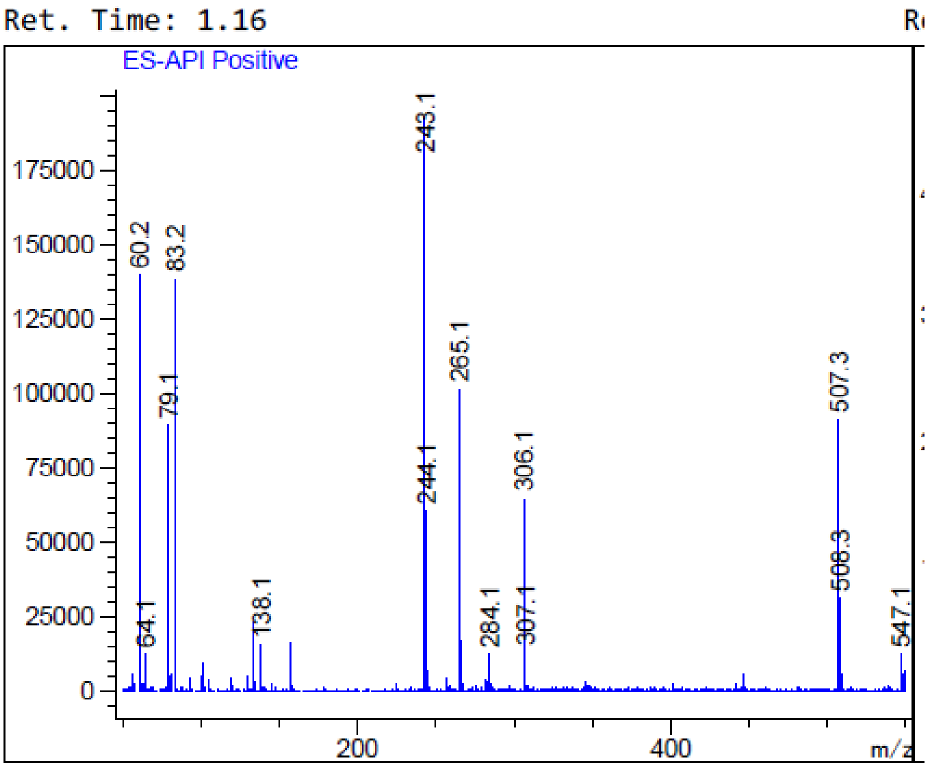


**3-(5-(2-oxa-6-azaspiro[3.3]heptan-6-yl)pyridin-2-yl)oxetane-3-carbonitrile (18)**

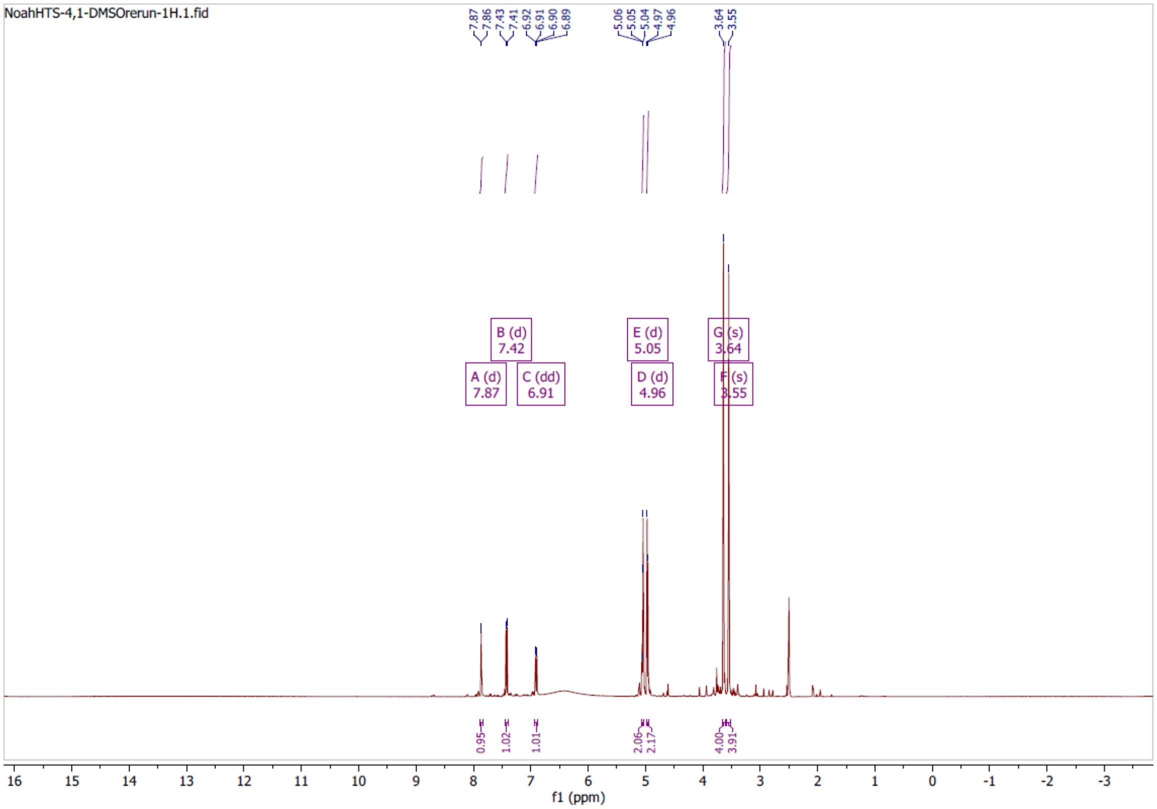

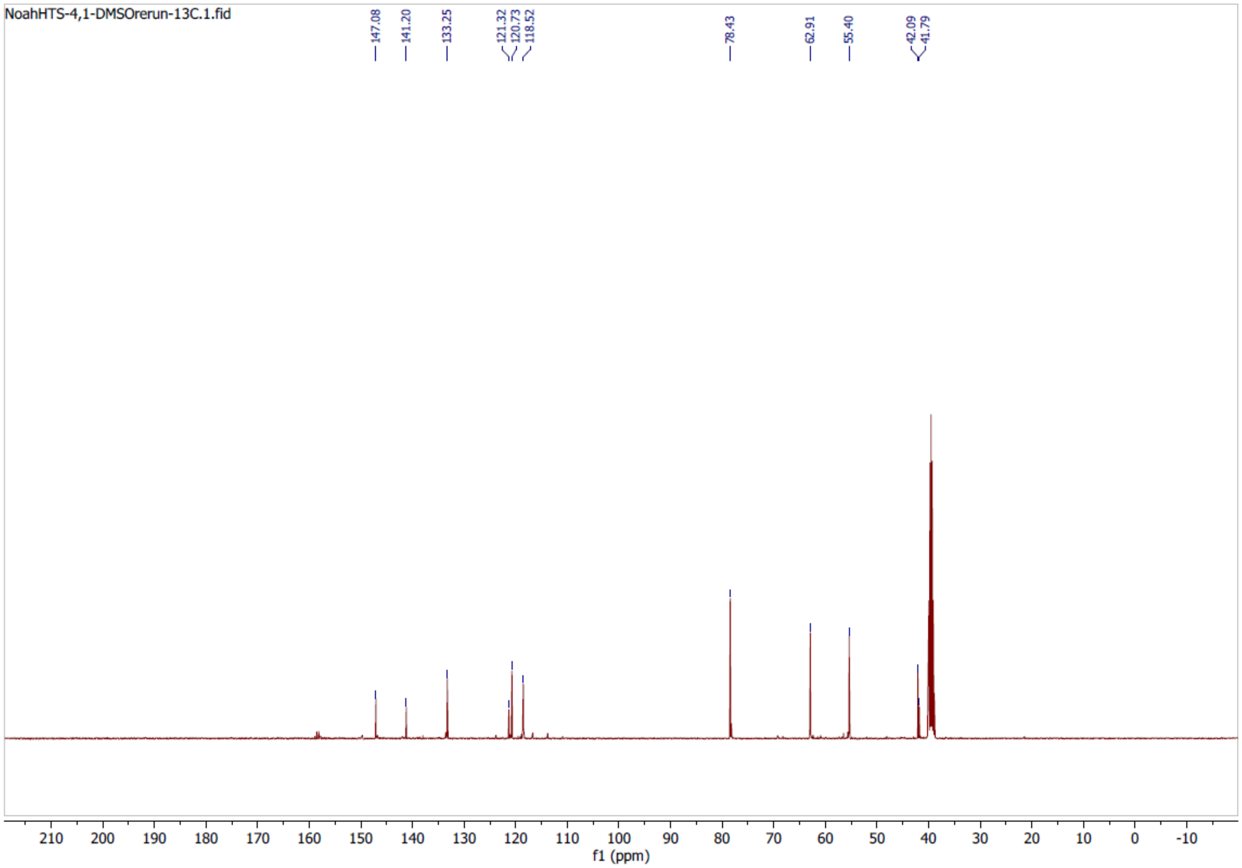
NoahHTS-4,1-DMSOrerun-13C.1.fid
147.08
141.20
133.25
121.32
120.73
118.52
78.43
62.91
55.40
42.09
41.79
f1 (ppm)

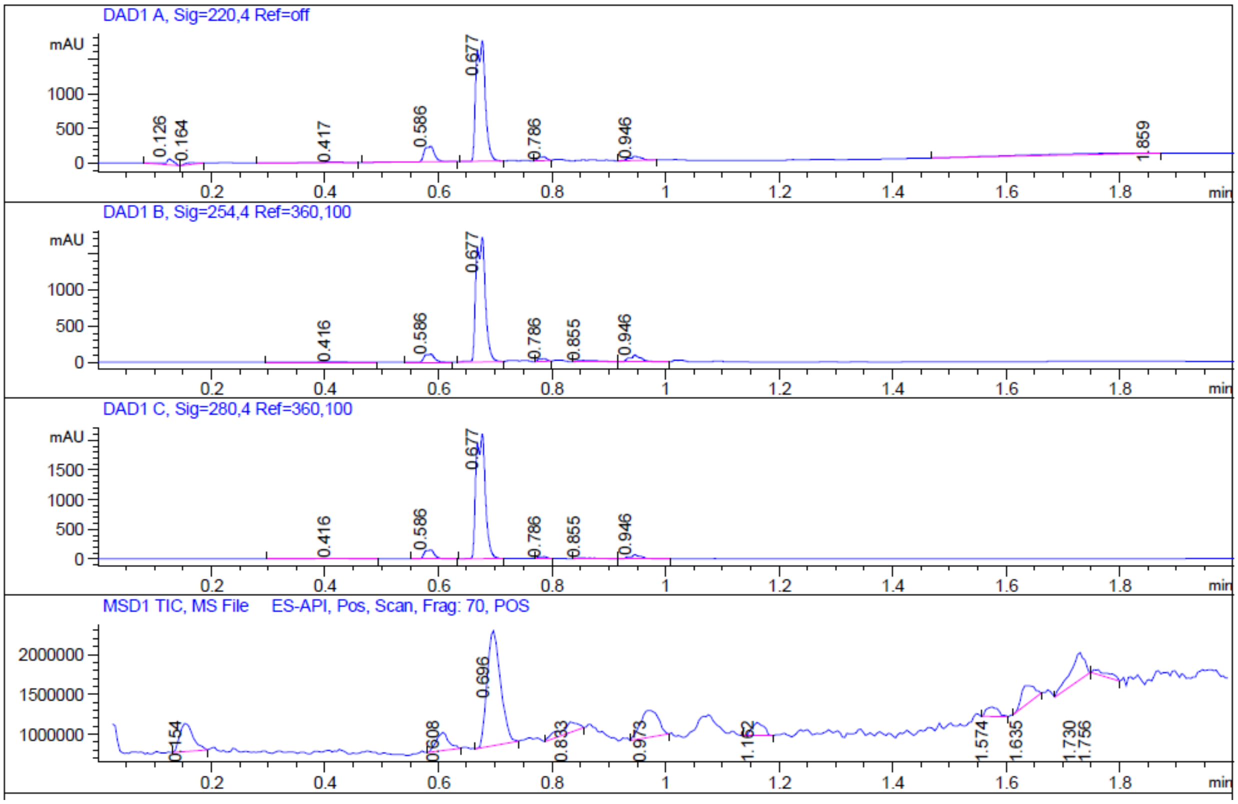
DAD1 A, Sig=220,4 Ref=off
DAD1 B, Sig=254,4 Ref=360,100
DAD1 C, Sig=280,4 Ref=360,100
MSD1 TIC, MS File ES-API, Pos, Scan, Frag: 70, POS
mAU
min

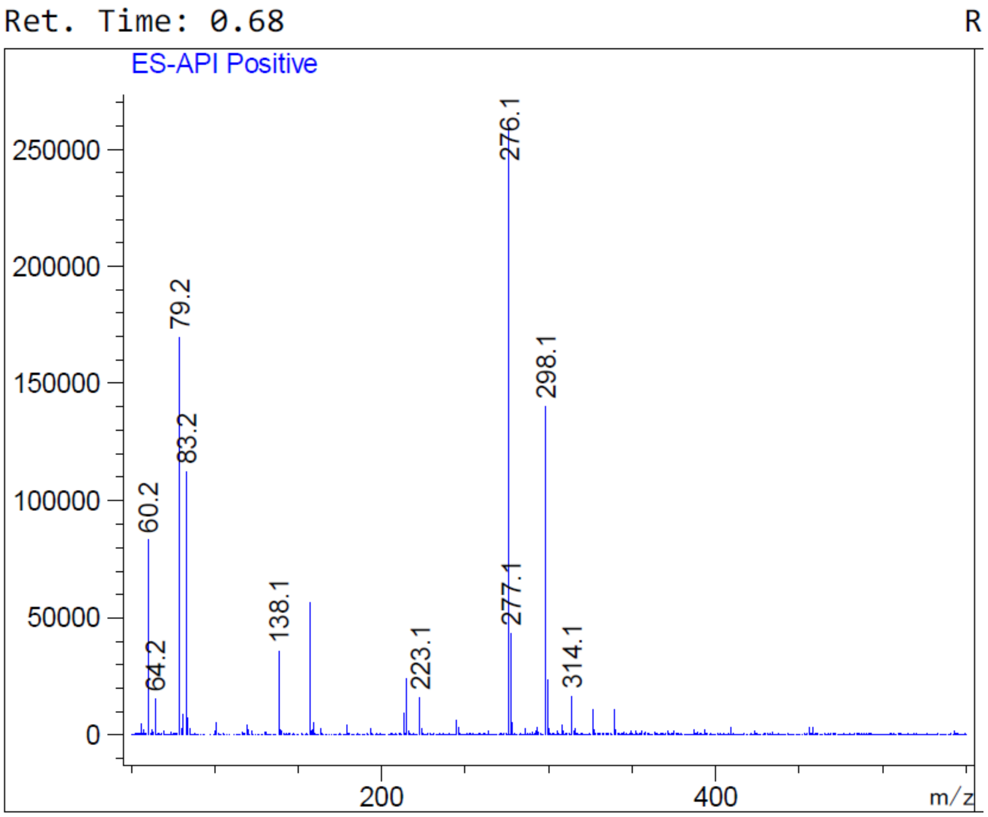


4-methyl-3-(2-oxa-6-azaspiro[3.3]heptan-6-yl)benzonitrile (19)

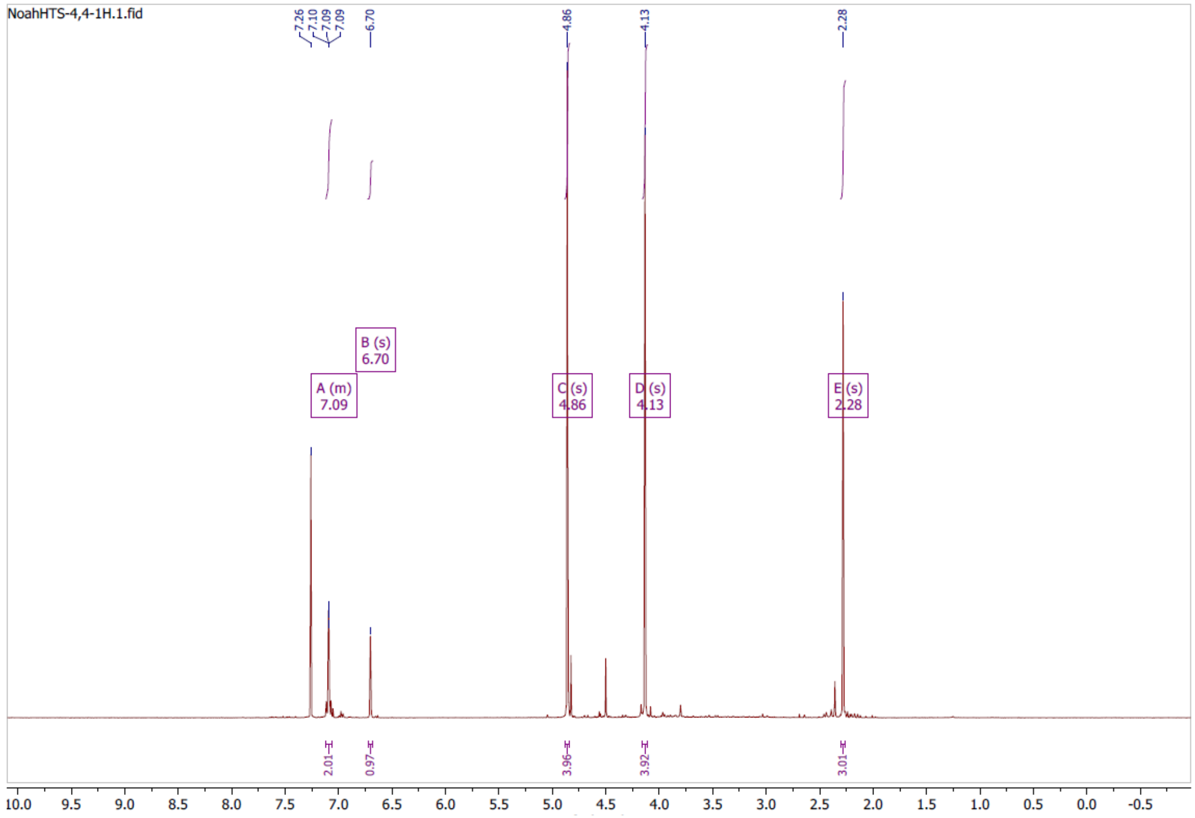

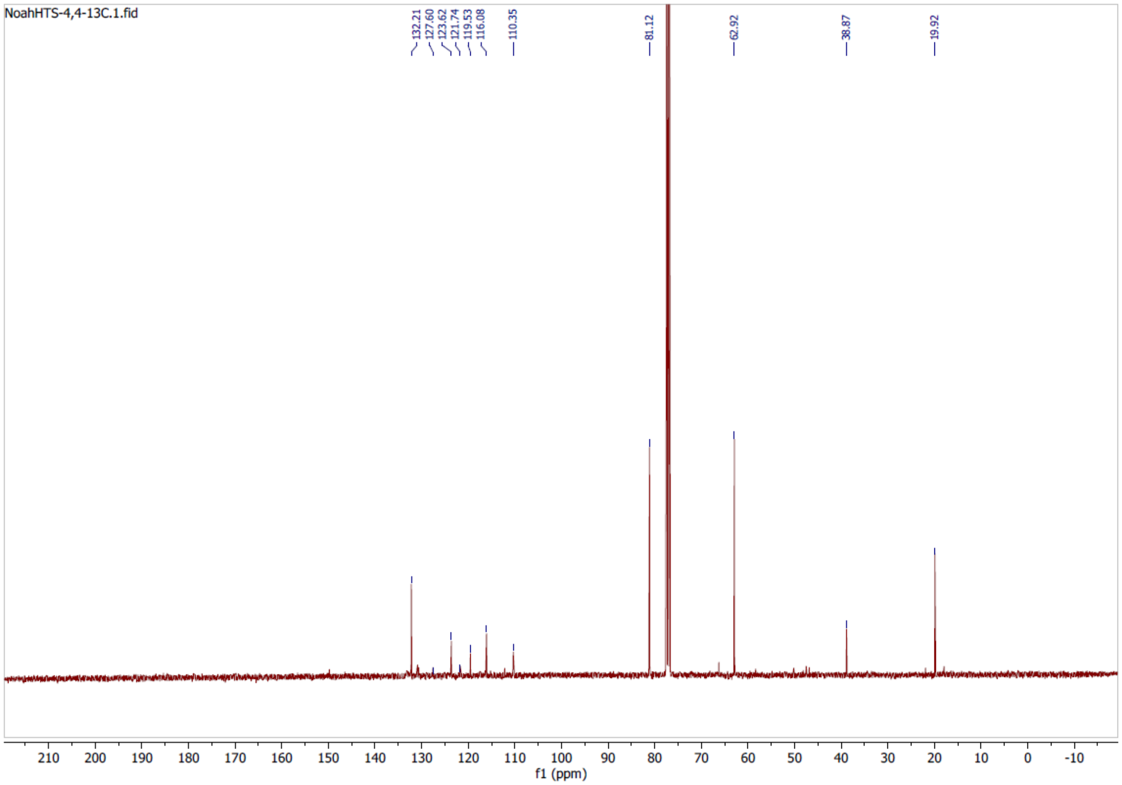
NoahHTS-4,4-13C.1.fid
132.21
127.60
123.62
121.74
119.53
116.08
110.35
81.12
62.92
38.87
19.92
f1 (ppm)

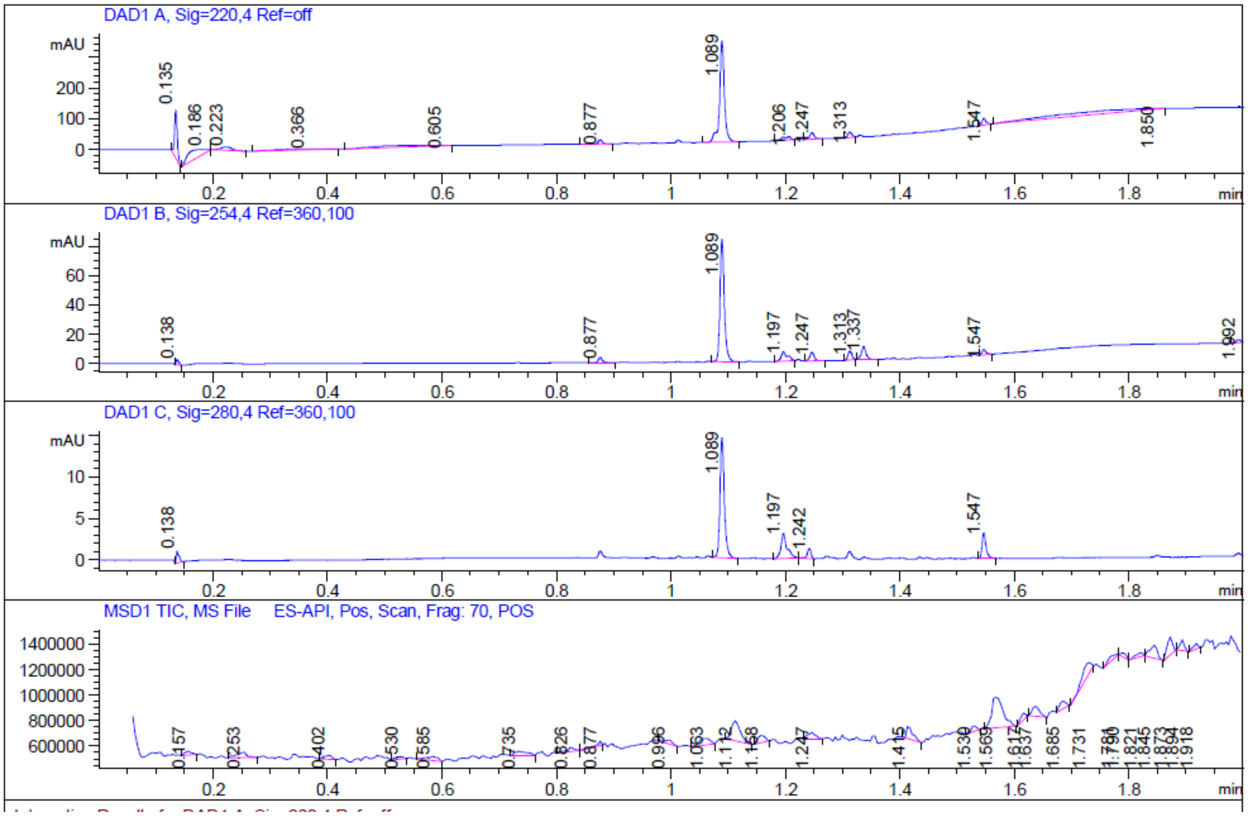
DAD1 A, Sig=220,4 Ref=off
mAU
DAD1 B, Sig=254,4 Ref=360,100
mAU
DAD1 C, Sig=280,4 Ref=360,100
mAU
MSD1 TIC, MS File ES-API, Pos, Scan, Frag: 70, POS
min

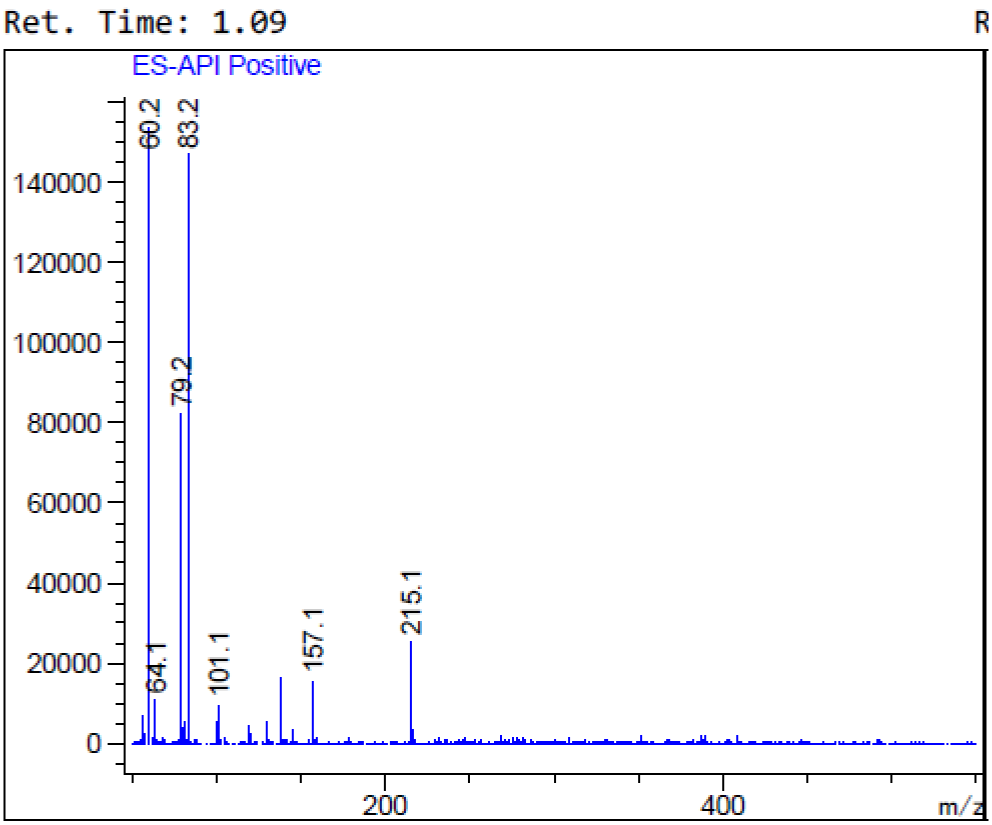


## References


(1) Geri, J. B.; Oakley, J. V; Reyes-Robles, T.; Wang, T.; McCarver, S. J.; White, C. H.; Rodriguez-Rivera, F. P.; Parker, D. L.; Hett, E. C.; Fadeyi, O. O.; et al. Microenvironment Mapping via Dexter Energy Transfer on Immune Cells. *Science (80-. ).* **2020**, *367* (6482), 1091–1097. https://doi.org/10.1126/science.aaz5074.

(2) Corcoran, E. B.; Pirnot, M. T.; Lin, S.; Dreher, S. D.; Dirocco, D. A.; Davies, I. W.; Buchwald, S. L.; Macmillan, D. W. C. Aryl Amination Using Ligand-Free Ni(II) Salts and Photoredox Catalysis. *Science (80-. ).* **2016**, *353* (6296), 279–283. https://doi.org/10.1126/science.aag0209.

(3) Neumann, M.; Füldner, S.; König, B.; Zeitler, K. Metal-Free, Cooperative Asymmetric Organophotoredox Catalysis with Visible Light. *Angew. Chemie - Int. Ed.* **2011**, *50* (4), 951–954. https://doi.org/10.1002/anie.201002992.

(4) Yuan, Y.; Shi, X.; Liu, W. Transition-Metal-Free, Chemoselective Aerobic Oxidations of Sulfides and Alcohols with Potassium Nitrate and Pyridinium Tribromide or Bromine. *Synlett* **2011**, *2011* (4), 559–564. https://doi.org/10.1055/s-0030-1259516.

(5) Yerien, D. E.; Cooke, M. V.; García Vior, M. C.; Barata-Vallejo, S.; Postigo, A. Radical Fluoroalkylation Reactions of (Hetero)Arenes and Sulfides under Red Light Photocatalysis. *Org. Biomol. Chem.* **2019**, *17* (15), 3741–3746. https://doi.org/10.1039/C9OB00486F.
